# 3-(Adenosylthio)benzoic Acid Derivatives as SARS-CoV-2 Nsp14 Methyltransferase Inhibitors

**DOI:** 10.3390/molecules28020768

**Published:** 2023-01-12

**Authors:** Olga Bobileva, Raitis Bobrovs, Evelina Elva Sirma, Iveta Kanepe, Anna L. Bula, Liene Patetko, Anna Ramata-Stunda, Solveiga Grinberga, Aigars Jirgensons, Kristaps Jaudzems

**Affiliations:** 1 Latvian Institute of Organic Synthesis, LV-1006 Riga, Latvia; 2 Faculty of Biology, University of Latvia, LV-1004 Riga, Latvia

**Keywords:** SARS-CoV-2, nsp14, nsp16, methyltransferase inhibitors

## Abstract

SARS-CoV-2 nsp14 guanine-*N*7-methyltransferase plays an important role in the viral RNA translation process by catalyzing the transfer of a methyl group from *S*-adenosyl-methionine (SAM) to viral mRNA cap. We report a structure-guided design and synthesis of 3-(adenosylthio)benzoic acid derivatives as nsp14 methyltransferase inhibitors resulting in compound **5p** with subnanomolar inhibitory activity and improved cell membrane permeability in comparison with the parent inhibitor. Compound **5p** acts as a bisubstrate inhibitor targeting both SAM and mRNA-binding pockets of nsp14. While the selectivity of 3-(adenosylthio)benzoic acid derivatives against human glycine *N*-methyltransferase was not improved, the discovery of phenyl-substituted analogs **5p,t** may contribute to further development of SARS-CoV-2 nsp14 bisubstrate inhibitors.

## 1. Introduction

Methyltransferases (MTases) are a large class of enzymes that play a vital role in various physiological processes and diseases by methylation of DNA, RNA, proteins, and carbohydrates. Viral MTase inhibitors have attracted attention in antiviral drug discovery for various pathogens including flaviviruses [1,2,3,4], alphaviruses [5], and coronaviruses.

Coronavirus MTases are highly conserved self-encoded nonstructural proteins nsp14 and nsp16 responsible for the methylation of viral RNA 5′-end cap [6]. A cap structure is *N*7-methylguanosine connected to the RNA through a triphosphate bridge and methylated 2′-*O*-group of the nucleotide [7]. Nsp14 is a guanine-*N*7-MTase and nsp16 in complex with nsp10 acts as a ribose 2′-*O*-MTase. RNA cap methylation is essential for virus vitality, mRNA guanine *N*-7-methylation is required for viral RNA translation into proteins and 2′*O*-methylation of the nucleotide protects the viral RNA from the cell immune system [8]. SARS-CoV-2 nsp14 is also a translation inhibitor, which suppresses host protein synthesis, including the production of antiviral proteins [9].

Efforts to target coronavirus MTases were triggered by SARS-CoV-1 and Middle East respiratory syndrome coronavirus emergencies in 2003 and 2012, respectively. Nevertheless, the studies led to the discovery of only a few MTase inhibitors. Sinefungin, a natural antifungal antibiotic isolated from *Streptomyces*, was found as an inhibitor for several viral MTases including SARS-CoV-1 [10], Zika virus [11], and Chikungunya virus [5]. Other non-selective MTase inhibiting small molecules are aurintricarboxylic acid and *S*-adenosyl-homocysteine (SAH) [12]. After the start of the COVID-19 pandemic, the development of coronavirus antivirals resumed. Several new nsp14 inhibitors were discovered via drug-repurposing studies and high-throughput screening both in silico and in vitro [13,14,15]. Although newly discovered compounds clearly inhibit MTase activity, their binding modes to the protein remain largely unknown. Another approach for the discovery of SARS-CoV-2 MTase inhibitors is a substrate-based drug design. Nsp14 and nsp16 use S-adenosyl-methionine (SAM) as a methyl group donor to transfer it to the RNA cap converting SAM to SAH.

Numerous SAM analogs have been developed as DNA and protein MTase inhibitors [16], as well as viral MTase inhibitors, but only a few modifications of SAM were explored specifically for coronavirus MTases. Importantly, SAM-dependent MTases are highly abundant proteins in the human organism. Therefore, selectivity is one of the main challenges in SAM-based drug design. Other difficulties are associated with poor drug-like properties of SAM analogs because nucleoside-derived structures often are polar compounds with low solubility and cell membrane permeability.

**Figure 1:**
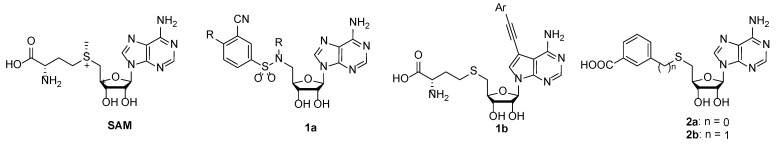
Structure of methyltransferase substrate *S*-adenosyl-methionine (SAM) and SARS-CoV-2 nsp14 inhibitors **1a**, **1b**, **2a**, and **2b**.

SAM structure consists of amino acid moiety and adenosine. A rational design of nsp14 bisubstrate inhibitors was reported, where methionine was substituted with various *N*-alkyl-benzenesulfonamides to target both SAM and RNA substrate-binding pockets (**1a**, Figure 1) [17,18,19]. Other research groups focused on substituted 7-deazaadenosine derivatives resulting in nanomolar nsp14 inhibitors **1b** [20,21]. Recently we reported the development of coronavirus nsp14 and nsp16/nsp10 inhibitors, by bioisosteric replacement of methionine which resulted in 3-(adenosylthio)benzoic acid (**2a**) and 3-(adenosylthio)methylbenzoic acid (**2b**) (Figure 1) with nanomolar potencies [22]. Here we report a structure–activity relationship (SAR) study to explore the chemical space of benzoic acid substructures of inhibitors **2a,b**. Our strategy includes the bioisosteric replacement of carboxylic acid and the introduction of additional substituents at the benzene ring to improve interactions with nsp14 SAM-binding pocket and to increase the cell membrane permeability. The biological activity of synthesized compounds was tested using a homogeneous time-resolved fluorescence (HTRF) assay [23] on nsp14. HTRF assay on human glycine N-methyltransferase (hGNMT) and nsp16/nsp10 was performed to determine the selectivity profile of the prepared compounds.

## 2. Results and Discussion

### 2.1. Chemistry

**Scheme 1:**
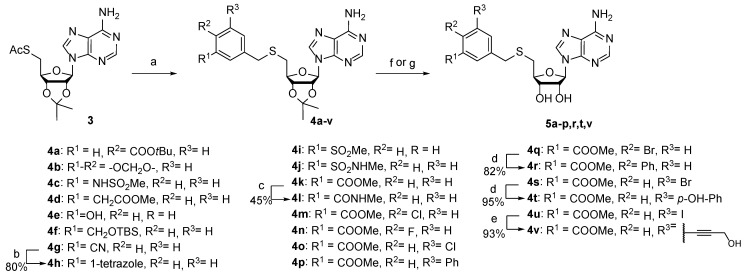
Synthesis of 3-(adenosylthio)methylbenzoic acid derivatives **5a–p,r,t,v**. Reagents, conditions, and yields: (**a**) ArCH_2_Br, NaOMe, MeOH, −30 °C to rt, 53–95%; (**b**) NH_4_Cl, NaN_3_, DMF, 110 °C; (**c**) i 2N NaOH, THF, 50–70 °C; ii MeNH_2_ (2 M in THF), HBTU, TEA, DMF, rt; (**d**) appropriate boronic acid, Pd(PPh_3_)_4_, K_2_CO_3_, dioxane/water, 100 °C, 82–95%; (**e**) propargyl alcohol, Pd(PPh_3_)_4_, CuI, TEA, MeCN, 35 °C; (**f**) 50% HCOOH, 37–88%; (**g**) i LiOH, THF, MeOH, water; ii 50% HCOOH, 24–71%.

**Scheme 2:**
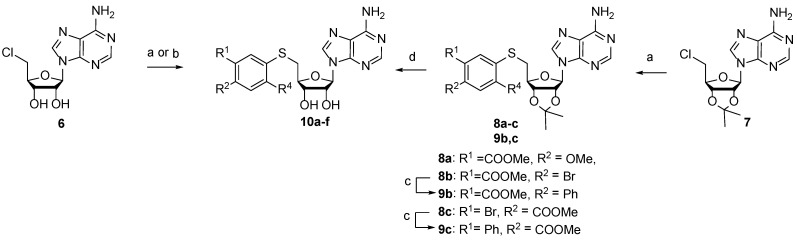
Synthesis of 3-(adenosylthio)benzoic acid derivatives **10a–f**. Reagents, conditions, and yields: (**a**) ArSH, KO*t*Bu, DMF, 0 °C to rt, 48–82%; (**b**) ArSH, NaOMe, DMF, −30 °C to rt, 30–38%; (**c**) phenylboronic acid, Pd(PPh_3_)_4_, K_2_CO_3_, dioxane/water, 100 °C, 59–84%; (**d**) *i* LiOH, THF, MeOH, water; *ii* 50% HCOOH, 35–72%.

The synthesis of the target compounds was accomplished as depicted in Scheme 1 and Scheme 2. *S*-benzyl-5′-thioadenosines **4a–g,i–k,m–q,s,u** were prepared from *S*-acetyl-5′-thioadenosine (**3**) in one-pot deacetylation and subsequent thiolate reaction with the corresponding benzylbromides. Tetrazole derivative **4h** was synthesized from arylnitrile **4g** and sodium azide in the presence of ammonium chloride. Biphenyls **4r,t** were obtained from arylbromides **4q,s** using Suzuki–Miyaura coupling. Alcohol **4v** was prepared by Sonogashira reaction of aryliodide **4u** with propargyl alcohol. Intermediates **4a–p,r,t,v** were subjected to the cleavage of protecting groups to afford corresponding SAM analogs **5a–p,r,t,v**.

*S*-aryl-5′-thioadenosines were prepared from corresponding thiophenols using alkylation reactions with 5′-chloro-5′-deoxyadenosine (**6**) or acetonide-protected analog **7** (Scheme 2). The target compounds **10d–f** were obtained after methyl ester hydrolysis and acetonide deprotection.

### 2.2. SARS-CoV-2 Nsp14 Inhibitory Activity and Structure–Activity Relationships

**Figure 2:**
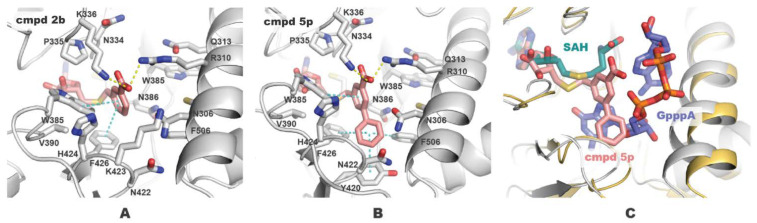
Docked poses of compounds **2b** (**A**) and **5p** (**B**) in the complex with nsp14. Inhibitors and residue side chains within 6 Å from the inhibitors are shown as sticks. Hydrogen bond and π-stacking interactions are shown with yellow and cyan dashed lines. (**C**) Docked pose of compound **5p** overlaid with SARS-CoV-1 nsp14-SAH-GpppA complex (PDB ID: 5C8S, 100% residue identity in the active site). The figure was generated using PyMOL.

A series of benzoic acid **2b** analogs **5a–l** were obtained where the position of the carboxylic acid group at the benzene ring was changed, or carboxylic acid was substituted with other possible hydrogen bond donors or acceptors. Tetrazole, sulfonamide, ester, amide, and other groups can form interactions with the polar amino acids Arg (R310), Asn (N334), and Lys (K336) in the SAM methionine subpocket of nsp14 (Figure 2A). The bioisosteric replacement of carboxylic acid can modify the physicochemical properties of the compound and can improve cell membrane permeability.

Evaluation of benzoic acid analogs **5a–l** biological activity showed a considerable drop in potency compared with the parent compound **2b** (Table 1). Neither of the compounds with carboxylic acid replacements exhibited activity similar to inhibitor **2b**. These results indicate that the interactions of negatively charged carboxylate with polar Arg (R310) and Lys (K336) in the SAM methionine-binding subpocket are essential to the inhibitor activity (Figure 2A). Furthermore, *meta*-position of carboxylate at the benzene ring is optimal for hydrogen bonding and ionic interactions, since *para*-benzoic acid analog **5a** lost its inhibitory activity 60-fold compared with compound **2b**.

Phenylacetic acid derivative **5d**, carboxylate substitution with a hydroxyl group (**5e,f**), esterification (**5k**) or amidation (**5l**) were not tolerated either. The most active compounds in the series are methanesulfonamide **5c**, nitrile **5g**, tetrazole **5h**, and methylsulfone **5i** with inhibitory activity in a range of 270–350 nM.

One of the most active compounds in this series methanesulfonamide **5c** and methyl ester **5k** as a potential pro-drug of carboxylic acid **2b** were tested in cell permeability assays using A549 cell line (Table 2). Compounds **5c** and **5k** showed some improvement in cell permeability compared with benzoic ester **2b** as was expected since the molecules contain more lipophilic substituents instead of carboxylic acid.

Next, we continued with the exploration of structure–activity relationships of substituted benzoic acids **10a–f**. Additional substituents at the benzene ring can form auxiliary interactions with the hydrophobic walls of the mRNA cap cavity. The enzyme inhibition potency data in Table 3 shows that simple modifications of benzoic acid **2a** can modulate the activity of aryl 5′-thioadenosines **10a–f**. The introduction of chlorine at benzoic acid 2-position resulted in a compound **10a** with 8-fold increase in potency compared with **2a**, whereas 4-chloro derivative **10b** showed an 8-fold reduction in potency. Bromine, methoxy group, or phenyl group at the 2-position of benzene ring (compounds **10c**–**e**) were well-tolerated and showed similar potency as 2-chloro derivative **10a**. According to the docking studies, the activity increase for the compounds **10a,c–e** is due to additional Van der Waals interactions, as the substituent is filling the SAM methionine-binding subpocket that otherwise would be partially solvated. An attempt to swap the positions of carboxylic acid and phenyl substituent led to a decrease in the activity of compound **10f** consistent with the results observed for compound **5a**.

We applied the benzene decoration strategy to generate analogs of inhibitor **2b.** The addition of 2-chloro substituent (**5m**) did not affect activity compared with the parent compound **2b**, whereas introducing more electronegative fluorine (**5n**) decreased activity twice, and phenyl substituent (**5r**) decreased activity 5-fold. Docking studies showed that the 2-phenyl group at the benzene ring of compound **5r** is solvent exposed and is too bulky for the methionine-binding pocket. 3-Chloro derivative **5o** showed a 3-fold reduction in activity compared with the parent compound **2b**. However, 3-phenyl group in **5p** was beneficial leading to subnanomolar inhibition potency. Such a result can be explained by additional hydrophobic interactions of the inhibitor with Tyr (Y420), Phe (F426), and Phe (F506) in the mRNA-binding subsite (Figure 2B). Structure **5p** binds to both SAM and mRNA cap-binding pockets (Figure 2C) acting as a bisubstrate nsp14 inhibitor. To target polar groups of Asn (N422) and Tyr (Y420) at the bottom of the hydrophobic mRNA cavity, we added 4-hydroxyl group at biphenyl analog **5t**, but it provided only a twofold increase in activity compared with the parent inhibitor **2b**. Hydroxypropynyl derivative **5v** slightly decreased activity showing possibly that the triple bond is not sufficient to form interactions in the mRNA subpocket.

Compounds **5p,r** and **10e** containing hydrophobic phenyl groups were also evaluated in the cell membrane permeability assay. This modification led to a slight improvement in cell permeability compared with analog **2b** (Table 2).

**Figure 3:**
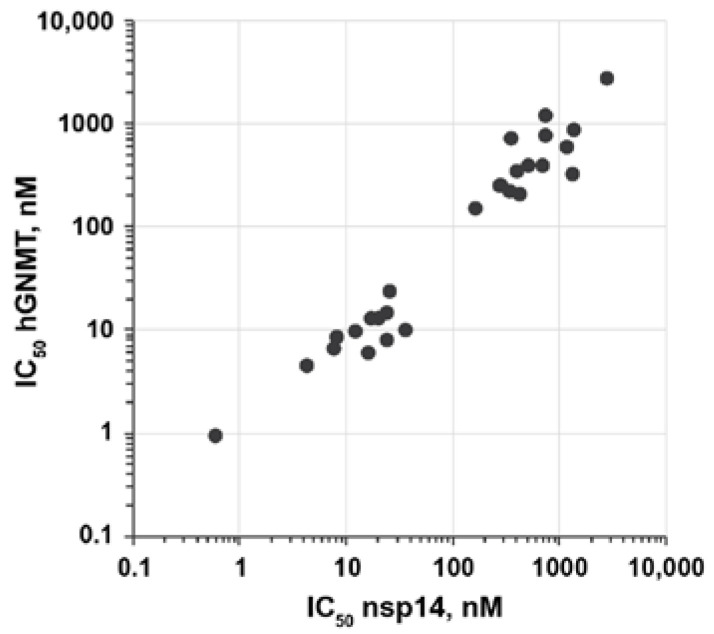
The correlation between inhibition potency (IC_50_) of nsp14 and hGNMT of compounds **5a–p,r,t,v** and **10a–f**. IC_50_ values are shown in logarithmic scale.

Modifications of 3-(adenosylthio)benzoic acids **2a,b** did not result in an improvement of the selectivity of SARS-CoV-2 nsp14 inhibition compared with hGNMT (Figure 3). None of the compounds showed more than twofold higher activity towards nsp14. Compounds **5a–e** and **10a,b** were also tested for their ability to inhibit the nsp16/nsp10 complex. The bioisosteric benzoic acid substitution led to a slight improvement in selectivity toward nsp14 inhibition compared with nsp16/nsp10 for compounds **5a–e**. In contrast, unmodified **2a,b** were 2-4-fold more potent nsp16/nsp10 inhibitors (Table 1).

In summary, bioisosteric replacements of the carboxylic acid group showed that the presence of carboxylate at the *meta*-position of the benzene ring of SAM-based inhibitors is essential for the high nsp14 inhibitory activity of the compound. At the same time, the carboxylic acid group is responsible for the inability of inhibitors to cross the cell membrane since the carboxylate replacement with methyl ester or methanesulfonamide improved the cell permeability of the compounds. The introduction of substituents at the benzene ring of inhibitors can significantly modulate the activity of SAM analogs **2a,b**. The activity of 3-adenosylthiobenzoic acid **2a** was improved 8-9-fold by the introduction of chloro, bromo, methoxy, or phenyl substituent at the 2-position of benzoic acid. The introduction of 3-phenyl group in 3-(adenosylthiomethyl)benzoic acid resulted in compound **5p** with subnanomolar nsp14 inhibitory activity and slightly increased cell membrane permeability. Docking results suggest that 3-phenylbenzoic acid derivatives **5p,t** are nsp14 bisubstrate inhibitors occupying both SAM and mRNA-binding sites. The analogs of inhibitors **2a,b** did not exhibit improvement in selectivity towards SARS-CoV-2 nsp14 compared with hGNMT, which is the challenge to be addressed in the next stage of the development of SAM-based coronavirus methyltransferase inhibitors.

## 3. Materials and Methods

### 3.1. Chemistry

#### 3.1.1. General

Reagents and dry solvents (DMF, acetonitrile, and methanol) were obtained from commercial sources and used without purification. Synthesis of benzyl bromides and mercaptobenzoates is described in supporting information. Dry THF and DCM were prepared on MB-SPS MBraun solvent purifier system. Reaction conditions and yields were not optimized. Normal phase chromatography was performed on Davisil 60 Å 35–70 μm silica and reverse-phase chromatography was performed using KP-C18-HS SNAP Biotage cartridges on a Biotage Isolera One purification system. Reactions were monitored by thin-layer chromatography using Merck F254 Alumina Silica Plates using UV visualization or staining. NMR spectra were recorded on 300 or 400 MHz Bruker spectrometers. Chemical shifts are reported in parts per million and referenced to the residual solvent signal. HRMS (ESI+) was obtained on a Waters Synapt G2-Si Mass Spectrometer. Analytical HPLC data were obtained using Waters Alliance LC systems equipped with 2695 separation module with LiChrospher PR Select 4.0 × 250 mm or Apollo 5 μm C18 4.6 × 150 mm column and Waters 2489 dual absorbance detector. Gradient 0–100% over 15 min; solvent A: 5% acetonitrile in 0.1% H_3_PO_4_; solvent B: 95% acetonitrile in 0.1% H_3_PO_4_; flow rate: 1 mL/min; column temperature: 40 °C.

#### 3.1.2. General Procedure P1 for Sulfide Synthesis

To a solution of 5′-acetylthio-5′-deoxy-2′,3′-*O*-isopropylideneadenosine [22] (**3**) (1 eq) and appropriate benzyl bromide (1.1 eq) in dry MeOH (5 mL/mmol) sodium methoxide solution in MeOH (5.4 M, 2.2 eq) was added dropwise under argon atmosphere at −30 °C. The reaction mixture was stirred at −30 °C for 10 min, then allowed to warm to room temperature and stirred for 1–3 h. The reaction mixture was quenched by the addition of saturated NH_4_Cl solution, extracted with EtOAc, combined organic layers were washed with water, dried over anhydrous Na_2_SO_4_, and evaporated under reduced pressure. The residue was purified by chromatography on silica gel on Biotage, eluent EtOAc:EtOH 3:1 in petroleum ether to obtain the title compound.

#### 3.1.3. General Procedure P2 for Suzuki Coupling

To a mixture of aryl bromide (1 eq), appropriate boronic acid (1.1–1.2 eq), Pd(PPh_3_)_4_ (0.05 eq), and K_2_CO_3_ (3 eq) in a vial under argon was added a degassed mixture of dioxane and water (4:1, 13 mL/mmol), the vial was sealed and the mixture was stirred for 13–15 h at 100 °C. The reaction mixture was filtered through a pad of Celite, washing with EtOAc, the filtrate was concentrated. The residue was chromatographed on silica gel on Biotage, eluent EtOAc:EtOH 3:1 in petroleum ether to obtain the title compound.

#### 3.1.4. General Procedure P3 for Acetonide Deprotection

A solution of acetonide-protected compound in 50% HCOOH solution in water (5 mL/mmol) was stirred at room temperature for 22 h. The solvent was evaporated under reduced pressure, then co-evaporated with EtOH. The residue was purified by reverse-phase chromatography on Biotage, eluent MeCN in 0.1% HCOOH to obtain the title compound.

#### 3.1.5. General Procedure P4 for Methyl Ester Hydrolysis and Acetonide Deprotection

To a solution of methyl carboxylate (0.14 mmol, 1 eq) in THF-water mixture (1:1, 7 mL/mmol), several drops of MeOH was added LiOH (3 eq) and the reaction mixture was stirred for 6–22 h at 50 °C. The solvent was evaporated under reduced pressure and the residue was dissolved in 50% HCOOH solution in water (5 mL/mmol). The reaction mixture was stirred at 50 °C for 5–7 h or room temperature for 18–24 h. The solvent was evaporated under reduced pressure and then co-evaporated with EtOH. The residue was purified by reverse-phase chromatography on Biotage, eluent MeCN in 0.1% HCOOH to obtain the title compound.

#### 3.1.6. Synthesis

*Tert*-Butyl 4-(((2′,3′-*O*-isopropylideneadenosyl)thio)methyl)benzoate (**4a**): Synthesized following general procedure P1 from **3** (0.09 g, 0.25 mmol) and *tert*-butyl 4-(bromomethyl)benzoate (74 mg, 0.27 mmol) yielded 0.11 g, 90% as a white foam. See the following: ^1^H NMR (400 MHz, CDCl_3_), δ 8.31 (s, 1H), 7.89 (s, 1H), 7.91–7.85 (m, 2H), 7.28–7.23 (m, 2H), 6.04 (d, *J =* 2.2 Hz, 1H), 5.62 (s, 2H), 5.46 (dd, *J =* 6.4, 2.2 Hz, 1H), 4.98 (dd, *J =* 6.4, 3.4 Hz, 1H), 4.32 (td, *J =* 6.7, 3.4 Hz, 1H), 3.72 (s, 2H), 2.75 (dd, *J =* 13.8, 7.0 Hz, 1H), 2.63 (dd, *J =* 13.8, 6.4 Hz, 1H), 1.60 (s, 3H), 1.58 (s, 9H), and 1.38 (s, 3H); ^13^C NMR (100 MHz, CDCl_3_) δ 165.6, 155.8, 153.3, 149.3, 142.7, 140.1, 131.0, 129.8, 128.8, 120.4, 114.7, 90.8, 86.7, 84.1, 83.9, 81.1, 36.3, 33.4, 28.3, 27.2, and 25.5. HRMS (ESI/TOF-Q) m/z: [M + H]^+^ calcd. for C_25_H_32_N_5_O_5_S, 514.2137; found, 514.2119.

5′-(((Benzo[d][1,3]dioxol-5-ylmethyl)thio)methyl)-5′-deoxy-2′,3′-*O-*isopropylideneadenosine (**4b**): Synthesized following general procedure P1 from **3** (0.09 g, 0.25 mmol) and 5-(bromomethyl)benzo[d][1,3]dioxole (58.0 mg, 0.27 mmol) yielded 82 mg, 73% as a white foam. See the following: ^1^H NMR (400 MHz, CDCl_3_) δ 8.31 (s, 1H), 7.90 (s, 1H), 6.76 (d, *J =* 1.7 Hz, 1H), 6.65 (d, *J =* 7.9 Hz, 1H), 6.60 (dd, *J =* 7.9, 1.7 Hz, 1H), 6.05 (d, *J =* 2.2 Hz, 1H), 5.93 (br s, 2H), 5.92 (s, 2H), 5.46 (dd, *J =* 6.4, 2.2 Hz, 1H), 5.00 (dd, *J =* 6.4, 3.3 Hz, 1H), 4.38–4.30 (m, 1H), 3.61 (s, 2H), 2.75 (dd, *J =* 13.7, 7.2 Hz, 1H), 2.65 (dd, *J =* 13.7, 6.3 Hz, 1H), 1.60 (s, 3H), and 1.38 (s, 3H); ^13^C NMR (100 MHz, CDCl_3_) δ 155.8, 153.3, 149.4, 148.0, 146.8, 140.1, 131.6, 122.2, 120.5, 114.6, 109.3, 108.1, 101.2, 90.9, 86.8, 84.1, 83.9, 36.5, 33.2, 27.2, and 25.5. HRMS (ESI/TOF-Q) m/z: [M + H]^+^ calcd. for C_21_H_24_N_5_O_5_S, 458.1500; found, 458.1493.

*N*-(3-(((2′,3′-*O*-isopropylideneadenosyl)thio)methyl)phenyl)methanesulfonamide (**4c**): Synthesized following general procedure P1 from **3** (0.09 g, 0.25 mmol) and *N*-(3-(bromomethyl)phenyl)methanesulfonamide (72.0 mg, 0.27 mmol) yielded 109 mg, 87% as a white foam. See the following: ^1^H NMR (400 MHz, CDCl_3_) δ 8.89 (s, 1H), 8.27 (s, 1H), 8.17 (s, 1H), 7.26–7.20 (m, 2H), 7.04 (d, *J =* 2.1 Hz, 1H), 6.99 (dt, *J =* 6.4, 1.9 Hz, 1H), 6.22 (s, 2H), 6.14 (d, *J =* 1.8 Hz, 1H), 5.42 (dd, *J =* 6.3, 1.8 Hz, 1H), 4.88 (dd, *J =* 6.3, 2.7 Hz, 1H), 4.49 (ddd, *J =* 8.3, 5.4, 2.7 Hz, 1H), 3.75–3.66 (m, 2H), 3.02 (s, 3H), 2.72 (dd, *J =* 13.0, 5.4 Hz, 1H), 2.64 (dd, *J =* 13.0, 8.3 Hz, 1H), 1.59 (s, 3H), and 1.37 (s, 3H); ^13^C NMR (100 MHz, CDCl_3_) δ 155.8, 153.5, 149.2, 140.0, 139.5, 137.6, 130.0, 125.6, 121.0, 120.0, 120.0, 114.5, 91.8, 87.3, 84.7, 84.2, 39.7, 36.7, 34.9, 27.1, and 25.4. HRMS (ESI/TOF-Q) m/z: [M + H]^+^ calcd. for C_21_H_27_N_6_O_5_S_2_, 507.1479; found, 507.1494.

Methyl 2-(3-(((2′,3′-*O*-isopropylideneadenosyl)thio)methyl)phenyl)acetate (**4d**): Synthesized following general procedure P1 from **3** (0.09 g, 0.25 mmol) and methyl 2-(3-(bromomethyl)phenyl)acetate (67.0 mg, 0.28 mmol) yielded 81 mg, 66% as a white foam. See the following: ^1^H NMR (300 MHz, CDCl_3_) δ 8.30 (s, 1H), 7.91 (s, 1H), 7.25–7.18 (m, 1H), 7.18–7.08 (m, 3H), 6.06 (d, *J =* 2.3 Hz, 1H), 5.65 (s, 2H), 5.46 (dd, *J =* 6.4, 2.3 Hz, 1H), 4.98 (dd, *J =* 6.4, 3.3 Hz, 1H), 4.35 (td, *J =* 6.8, 3.3 Hz, 1H), 3.69 (s, 2H), 3.67 (s, 3H), 3.59 (s, 2H), 2.76 (dd, *J =* 13.7, 7.2 Hz, 1H), 2.66 (dd, *J =* 13.7, 6.3 Hz, 1H), 1.60 (s, 3H), and 1.38 (s, 3H); ^13^C NMR (100 MHz, CDCl_3_) δ 172.0, 155.7, 153.1, 149.3, 140.1, 138.2, 134.4, 129.9, 128.9, 128.2, 127.8, 120.4, 114.6, 90.8, 86.6, 84.1, 83.9, 52.2, 41.1, 36.5, 33.5, 27.2, and 25.5. HRMS (ESI/TOF-Q) m/z: [M + H]^+^ calcd. for C_23_H_28_N_5_O_5_S, 486.1806; found, 486.1812.

3-(((2′,3′-*O*-Isopropylideneadenosyl)thio)methyl)phenol (**4e**): Synthesized following general procedure P1 from **3** (0.10 g, 0.27 mmol) and (3-(bromomethyl)phenoxy)(*tert*-butyl)dimethylsilane (0.09 g, 0.30 mmol) yielded 62 mg, 53% as a white foam. See the following: ^1^H NMR (400 MHz, CDCl_3_) δ 8.33 (s, 1H), 8.04 (s, 1H), 7.12 (t, *J =* 7.8 Hz, 1H), 6.75 (ddd, *J =* 7.6, 1.6, 0.9 Hz, 1H), 6.71 (ddd, *J =* 8.1, 2.5, 0.9 Hz, 1H), 6.68–6.64 (m, 1H), 6.39 (s, 1H), 6.09 (d, *J =* 2.0 Hz, 1H), 5.59 (s, 2H), 5.51 (dd, *J =* 6.4, 2.0 Hz, 1H), 4.93 (dd, *J =* 6.4, 3.0 Hz, 1H), 4.42 (td, *J =* 6.8, 3.0 Hz, 1H), 3.63 (s, 2H), 2.70 (dd, *J =* 13.5, 6.4 Hz, 1H), 2.60 (dd, *J =* 13.5, 7.3 Hz, 1H), 1.60 (s, 4H), and 1.39 (s, 3H); ^13^C NMR (100 MHz, CDCl_3_) δ 156.6, 155.5, 153.3, 149.3, 140.2, 139.6, 129.9, 120.9, 120.0, 116.0, 114.8, 114.6, 91.3, 87.1, 84.3, 84.0, 36.6, 33.7, 27.2, and 25.5. HRMS (ESI/TOF-Q) m/z: [M + H]^+^ calcd. for C_20_H_24_N_5_O_4_S, 430.1544; found, 430.1543.

5′-((3-(((*tert*-Butyldimethylsilyl)oxy)methyl)benzyl)thio)-5′-deoxy-2′,3′-*O*-isopropylideneadenosine (**4f**): Synthesized following general procedure P1 from **3** (0.10 g, 0.27 mmol) and ((3-(bromomethyl)benzyl)oxy)(*tert*-butyl)dimethylsilane (0.10 g, 0.32 mmol), yielded 0.11 g, 73% as a colorless oil. See the following: ^1^H NMR (400 MHz, CDCl_3_) δ 8.30 (s, 1H), 7.90 (s, 1H), 7.26–7.16 (m, 3H), 7.11–7.05 (m, 1H), 6.05 (d, *J =* 2.3 Hz, 1H), 5.84 (s, 2H), 5.45 (dd, *J =* 6.5, 2.3 Hz, 1H), 4.98 (dd, *J =* 6.5, 3.3 Hz, 1H), 4.70 (s, 2H), 4.35 (ddd, *J =* 7.3, 6.1, 3.3 Hz, 1H), 3.70 (s, 2H), 2.76 (dd, *J =* 13.7, 7.3 Hz, 1H), 2.65 (dd, *J =* 13.7, 6.1 Hz, 1H), 1.60 (s, 3H), 1.38 (s, 3H), 0.92 (s, 9H), and 0.08 (s, 6H); ^13^C NMR (100 MHz, CDCl_3_) δ 155.7, 153.3, 149.4, 142.0, 140.1, 137.8, 128.5, 127.6, 126.6, 125.1, 120.5, 114.6, 90.9, 86.6, 84.2, 83.9, 64.9, 36.6, 33.4, 27.3, 26.1, 25.5, 18.5, and 5.1. HRMS (ESI/TOF-Q) m/z: [M + H]^+^ calcd. for C_27_H_40_N_5_O_4_Ssi, 558.2565; found, 558.2578.

3-(((2′,3′-*O*-Isopropylideneadenosyl)thio)methyl)benzonitrile (**4g**): Synthesized following general procedure P1 from **3** (0.07 g, 0.36 mmol) and methyl 3-(bromomethyl)benzonitrile (0.13 g, 0.36 mmol), yielded 90 mg, 60% as a white foam. See the following: ^1^H NMR (300 MHz, CDCl_3_) δ 8.29 (s, 1H), 7.88 (s, 1H), 7.55–7.53 (m, 1H), 7.50 (dt, *J =* 7.5, 1.6 Hz, 1H), 7.41 (dt, *J =* 7.8, 1.6 Hz, 1H), 7.37–7.30 (m, 1H), 6.05 (d, *J =* 2.2 Hz, 1H), 5.58 (s, 2H), 5.47 (dd, *J =* 6.4, 2.2 Hz, 1H), 5.04 (dd, *J =* 6.4, 3.5 Hz, 1H), 4.33 (td, *J =* 6.7, 3.5 Hz, 1H), 3.69 (s, 2H), 2.78 (dd, *J =* 13.8, 6.6 Hz, 1H), 2.67 (dd, *J =* 13.9, 6.8 Hz, 1H), 1.61 (s, 3H), and 1.43–1.33 (m, 3H); ^13^C NMR (100 MHz, CDCl_3_) δ 155.6, 153.1, 149.2, 140.9, 140.1, 139.9, 134.0, 129.5, 127.6, 126.1, 120.3, 114.7, 90.7, 86.9, 84.0, 83.8, 44.5, 36.1, 33.7, 27.1, and 25.4. HRMS (ESI/TOF-Q) m/z: [M + H]^+^ calcd. for C_21_H_23_N_6_O_3_S, 439.1547; found, 439.1561.

5′-((3-(Methylsulfonyl)benzyl)thio)-5′-deoxy-2′,3′-*O*-isopropylideneadenosine (**4i**): Synthesized following general procedure P1 from **3** (0.10 g, 0.27 mmol) and 1-(bromomethyl)-3-(methylsulfonyl)benzene (purity 75%, 0.09 g, 0.27 mmol), yielded 84 mg, 66% as a white foam. See the following: ^1^H NMR (300 MHz, CDCl_3_) δ 8.30 (s, 1H), 7.90 (s, 1H), 7.86–7.83 (m, 1H), 7.80 (dt, *J =* 6.8, 2.0 Hz, 1H), 7.51–7.41 (m, 2H), 6.06 (d, *J =* 2.2 Hz, 1H), 5.61 (s, 2H), 5.48 (dd, *J =* 6.4, 2.2 Hz, 1H), 5.04 (dd, *J =* 6.4, 3.5 Hz, 1H), 4.35 (td, *J =* 6.7, 3.5 Hz, 1H), 3.76 (s, 2H), 3.04 (s, 3H), 2.80 (dd, *J =* 13.8, 6.7 Hz, 1H), 2.69 (dd, *J =* 13.8, 6.7 Hz, 1H), 1.61 (s, 3H), and 1.39 (s, 3H); ^13^C NMR (100 MHz, CDCl_3_) δ 155.6, 153.1, 149.2, 140.9, 140.1, 139.9, 134.0, 129.5, 127.6, 126.1, 120.3, 114.6, 90.7, 86.9, 84.0, 83.8, 44.5, 36.1, 33.7, 27.1, and 25.4. HRMS (ESI/TOF-Q) m/z: [M + H]^+^ calcd. for C_21_H_26_N_5_O_5_S_2_, 492.1370; found, 492.1378.

3-(((2′,3′-*O*-Isopropylideneadenosyl)thio)methyl)-*N*-methylbenzenesulfonamide (**4j**): Synthesized following general procedure P1 from **3** (0.11 g, 0.30 mmol) and 3-(bromomethyl)-*N*-methylbenzenesulfonamide (95 mg, 0.36 mmol), yielded 73 mg, 48% as a white foam. See the following: ^1^H NMR (400 MHz, CDCl_3_) δ 8.30 (s, 1H), 7.97 (s, 1H), 7.74 (dt, *J =* 7.0, 1.9 Hz, 1H), 7.61 (t, *J =* 1.6 Hz, 1H), 7.46–7.39 (m, 2H), 6.12 (d, *J =* 2.1 Hz, 1H), 5.83 (q, *J =* 5.3 Hz, 1H), 5.73 (s, 2H), 5.48 (dd, *J =* 6.4, 2.1 Hz, 1H), 4.98 (dd, *J =* 6.4, 3.4 Hz, 1H), 4.41 (td, *J =* 6.2, 3.4 Hz, 1H), 3.70 (d, *J =* 1.7 Hz, 2H), 2.77 (dd, *J =* 13.8, 6.0 Hz, 1H), 2.71 (dd, *J =* 13.8, 6.4 Hz, 1H), 2.67 (d, *J =* 5.3 Hz, 3H), 1.65–1.58 (m, 3H), and 1.39 (s, 3H); ^13^C NMR (100 MHz, CDCl_3_) δ 155.9, 153.3, 149.3, 140.0, 139.6, 139.4, 133.0, 129.3, 127.3, 126.2, 120.1, 114.8, 90.7, 86.8, 84.1, 83.7, 36.6, 34.1, 29.4, 27.2, and 25.5. HRMS (ESI/TOF-Q) m/z: [M + H]^+^ calcd. for C_21_H_27_N_6_O_5_S_2_, 507.1479; found, 507.1496.

Methyl 5-(((2′,3′-*O*-isopropylideneadenosyl)thio)methyl)benzoate (**4k**): Synthesized following general procedure P1 from **3** (0.44 g, 1.20 mmol) and methyl 3-(bromomethyl)-benzoate (0.31 g, 1.34 mmol), yielded 0.51 g, 91% as a white foam. See the following: ^1^H NMR (400 MHz, CDCl_3_) δ 8.28 (s, 1H), 7.93 (dt, *J =* 1.8, 0.6 Hz, 1H), 7.90 (s, 1H), 7.89 (ddd, *J =* 7.6, 1.8, 1.4 Hz, 1H), 7.41 (ddd, *J =* 7.6, 1.9, 1.4 Hz, 1H), 7.33 (td, *J =* 7.6, 0.6 Hz, 1H), 6.05 (d, *J =* 2.2 Hz, 1H), 5.76 (s, 2H), 5.45 (dd, *J =* 6.4, 2.2 Hz, 1H), 5.00 (dd, *J =* 6.4, 3.4 Hz, 1H), 4.34 (td, *J =* 6.7, 3.4 Hz, 1H), 3.92 (s, 3H), 3.74 (s, 2H), 2.77 (dd, *J =* 13.7, 7.0 Hz, 1H), 2.66 (dd, *J =* 13.7, 6.4 Hz, 1H), 1.60 (s, 3H), and 1.38 (s, 3H); ^13^C NMR (101 MHz, CDCl_3_) δ 166.9, 155.7, 153.2, 149.3, 140.1, 138.4, 133.4, 130.6, 130.0, 128.7, 128.5, 120.4, 114.7, 90.8, 86.8, 84.1, 83.9, 52.3, 36.3, 33.5, 27.2, and 25.5. HRMS (ESI/TOF-Q) m/z: [M + H]^+^ calcd. for C_22_H_26_N_5_O_5_S 472.1649, found 472.1675.

Methyl 5-(((2′,3′-*O*-isopropylideneadenosyl)thio)methyl)-2-chlorobenzoate (**4m**): Synthesized following general procedure P1 from **3** (0.08 g, 0.22 mmol) and methyl 5-(bromomethyl)-2-chlorobenzoate (90% purity, 71.0 mg, 0.24 mmol), yielded 90 mg, 81% as a white foam. See the following: ^1^H NMR (400 MHz, CDCl_3_) δ 8.28 (s, 1H), 7.88 (s, 1H), 7.71 (d, *J =* 2.3 Hz, 1H), 7.31 (d, *J =* 8.2 Hz, 1H), 7.23 (dd, *J =* 8.2, 2.3 Hz, 1H), 6.04 (d, *J =* 2.2 Hz, 1H), 5.96 (s, 2H), 5.46 (dd, *J =* 6.4, 2.2 Hz, 1H), 5.01 (dd, *J =* 6.4, 3.5 Hz, 1H), 4.32 (td, *J =* 6.7, 3.5 Hz, 1H), 3.91 (s, 3H), 3.67 (s, 2H), 2.76 (dd, *J =* 13.8, 6.8 Hz, 1H), 2.66 (dd, *J =* 13.8, 6.6 Hz, 1H), 1.59 (s, 3H), and 1.37(s, 3H); ^13^C NMR (100 MHz, CDCl_3_) δ 166.0, 155.8, 153.2, 149.3, 140.1, 136.9, 133.0, 132.5, 131.8, 131.3, 130.1, 120.4, 114.7, 90.8, 86.9, 84.1, 83.9, 52.6, 35.7, 33.6, 27.2, and 25.5. HRMS (ESI/TOF-Q) m/z: [M + H]^+^ calcd. for C_22_H_25_ClN_5_O_5_S, 506.1259; found, 506.1274.

Methyl 5-(((2′,3′-*O*-isopropylideneadenosyl)thio)methyl)-2-fluorobenzoate (**4n**): Synthesized following general procedure P1 from **3** (0.08 g, 0.22 mmol) and methyl 5-(bromomethyl)-2-fluorobenzoate (63.0 mg, 0.24 mmol), yielded 90 mg, 81% as a white foam. See the following: ^1^H NMR (300 MHz, CDCl_3_) δ 8.30 (s, 1H), 7.89 (s, 1H), 7.80 (dd, *J =* 6.8, 2.5 Hz, 1H), 7.35 (ddd, *J =* 8.5, 4.5, 2.5 Hz, 1H), 7.01 (dd, *J =* 10.4, 8.5 Hz, 1H), 6.05 (d, *J =* 2.2 Hz, 1H), 5.62 (s, 2H), 5.47 (dd, *J =* 6.4, 2.2 Hz, 1H), 5.02 (dd, *J =* 6.4, 3.4 Hz, 1H), 4.34 (td, *J =* 6.7, 3.4 Hz, 1H), 3.92 (s, 3H), 3.68 (s, 2H), 2.77 (dd, *J =* 13.8, 6.8 Hz, 1H), 2.66 (dd, *J =* 13.8, 6.6 Hz, 1H), 1.60 (s, 3H), and 1.38 (s, 3H); ^13^C NMR (100 MHz, CDCl_3_) δ 164.7 (*J_CF_* = 4.0 Hz), 161.0 (*J_CF_* = 266.0 Hz), 155.8, 153.2, 149.3, 140.1, 134.8 (*J_CF_* = 9.0 Hz), 134.0 (*J_CF_* = 3.8 Hz), 132.4, 120.4, 118.6 (*J_CF_* = 10.2 Hz), 117.3 (*J_CF_* = 22.7 Hz), 114.7, 90.8, 86.9, 84.1, 83.9, 52.5, 35.6, 33.6, 27.2, and 25.4. HRMS (ESI/TOF-Q) m/z: [M + H]^+^ calcd. for C_22_H_25_FN_5_O_5_S, 490.1555; found, 490.1559.

Methyl 5-(((2′,3′-*O*-isopropylideneadenosyl)thio)methyl)-3-chlorobenzoate (**4o**): Synthesized following general procedure P1 from **3** (0.07 g, 0.19 mmol) and methyl 5-(bromomethyl)-3-chlorobenzoate (60% purity, 93.0 mg, 0.21 mmol), yielded 74 mg, 76% as a white foam. See the following: ^1^H NMR (400 MHz, CDCl_3_) δ 8.31 (s, 1H), 7.88 (s, 1H), 7.86 (dd, *J =* 1.8 Hz, 1H), 7.79 (t, *J =* 1.6 Hz, 1H), 7.44 (t, *J =* 1.8 Hz, 1H), 6.04 (d, *J =* 2.2 Hz, 1H), 5.56 (s, 2H), 5.46 (dd, *J =* 6.5, 2.2 Hz, 1H), 5.03 (dd, *J =* 6.5, 3.5 Hz, 1H), 4.34 (td, *J =* 6.7, 3.5 Hz, 1H), 3.92 (s, 3H), 3.70 (s, 2H), 2.80 (dd, *J =* 13.8, 6.9 Hz, 1H), 2.70 (dd, *J =* 13.8, 6.6 Hz, 1H), 1.61 (s, 3H), and 1.38 (s, 3H); ^13^C NMR (100 MHz, CDCl_3_) δ 165.7, 155.8, 153.2, 149.2, 140.5, 140.1, 134.7, 133.2, 132.0, 128.6, 128.2, 120.4, 114.8, 90.8, 86.8, 84.1, 83.9, 52.6, 35.9, 33.7, 27.2, and 25.5. HRMS (ESI/TOF-Q) m/z: [M + H]^+^ calcd. for C_22_H_25_ClN_5_O_5_S, 506.1259; found, 506.1270.

Methyl 5-(((2′,3′-*O*-isopropylideneadenosyl)thio)methyl)-[1,1′-biphenyl]-3-carboxylate (**4p**): Synthesized following general procedure P1 from **3** (0.09 g, 0.25 mmol) and methyl 5-(bromomethyl)-[1,1′-biphenyl]-3-carboxylate (77% purity, 0.10 g, 0.25 mmol), yielded 98 mg, 73% as a white foam. See the following: ^1^H NMR (300 MHz, CDCl_3_) δ 8.28 (s, 1H), 8.14 (t, *J =* 1.5 Hz, 1H), 7.94–7.85 (m, 2H), 7.72–7.68 (m, 1H), 7.64–7.55 (m, 2H), 7.51–7.42 (m, 2H), 7.40–7.33 (m, 1H), 6.04 (d, *J =* 2.3 Hz, 1H), 5.59 (s, 2H), 5.46 (ddd, *J =* 6.4, 2.3, 1.0 Hz, 1H), 5.03 (dd, *J =* 6.4, 3.4 Hz, 1H), 4.40–4.32 (m, 1H), 3.94 (s, 3H), 3.81 (s, 2H), 2.83 (dd, *J =* 13.7, 7.1 Hz, 1H), 2.72 (ddd, *J =* 13.7, 6.3 Hz, 1H), 1.59 (s, 3H), and 1.37 (s, 3H); ^13^C NMR (100 MHz, CDCl_3_) δ 166.4, 155.7, 153.2, 149.3, 142.0, 140.1, 139.9, 132.0, 131.5, 131.1, 129.0, 128.8, 128.0, 127.3, 120.4, 114.7, 90.8, 86.6, 86.6, 84.1, 83.9, 61.3, 52.4, 36.5, 33.8, 27.2, 25.4, and 14.5. HRMS (ESI/TOF-Q) m/z: [M + H]^+^ calcd. for C_28_H_30_N_5_O_5_S, 548.1962; found, 548.1971.

Methyl 5-(((2′,3′-*O*-isopropylideneadenosyl)thio)methyl)-2-bromobenzoate (**4q**): Synthesized following general procedure P1 from **3** (0.24 g, 0.65 mmol) and methyl 5-(bromomethyl)-2-bromobenzoate (0.23 g, 0.71 mmol), yielded 0.33 g, 92% as a white foam. See the following: ^1^H NMR (400 MHz, CDCl_3_) δ 8.30 (s, 1H), 7.89 (s, 1H), 7.68 (d, *J =* 2.3 Hz, 1H), 7.52 (d, *J =* 8.2 Hz, 1H), 7.15 (dd, *J =* 8.2, 2.3 Hz, 1H), 6.04 (d, *J =* 2.2 Hz, 1H), 5.71 (s, 2H), 5.46 (dd, *J =* 6.4, 2.2 Hz, 1H), 5.01 (dd, *J =* 6.4, 3.4 Hz, 1H), 4.33 (td, *J =* 6.7, 3.4 Hz, 1H), 3.92 (s, 3H), 3.66 (s, 2H), 2.77 (dd, *J =* 13.8, 6.8 Hz, 1H), 2.66 (dd, *J =* 13.8, 6.6 Hz, 1H), 1.60 (s, 3H), and 1.38 (s, 3H); ^13^C NMR (100 MHz, CDCl_3_) δ 166.4, 155.8, 153.2, 149.3, 140.1, 137.6, 134.5, 133.0, 132.2, 131.8, 120.4, 120.3, 114.7, 90.8, 86.9, 84.1, 83.9, 52.6, 35.7, 33.6, 27.2, and 25.4. HRMS (ESI/TOF-Q) m/z: [M + H]^+^ calcd. for C_22_H_25_BrN_5_O_5_S, 550.0754; found, 550.0769.

Methyl 5-(((2′,3′-*O*-isopropylideneadenosyl)thio)methyl)-3-bromobenzoate (**4s**): Synthesized following general procedure P1 from **3** (0.28 g, 0.77 mmol) and methyl 5-(bromomethyl)-3-bromobenzoate (85% purity, 0.31 g, 0.84 mmol), yielded 0.40 g, 95% as a white foam. See the following: ^1^H NMR (400 MHz, CDCl_3_) δ 8.29 (s, 1H), 8.01 (t, *J =* 1.7 Hz, 1H), 7.88 (s, 1H), 7.82 (t, *J =* 1.6 Hz, 1H), 7.59 (t, *J =* 1.8 Hz, 1H), 6.04 (d, *J =* 2.2 Hz, 1H), 5.94 (s, 2H), 5.45 (dd, *J =* 6.4, 2.2 Hz, 1H), 5.02 (dd, *J =* 6.4, 3.5 Hz, 1H), 4.33 (td, *J =* 6.7, 3.5 Hz, 1H), 3.90 (s, 3H), 3.68 (s, 2H), 2.79 (dd, *J =* 13.8, 6.8 Hz, 1H), 2.69 (dd, *J =* 13.8, 6.6 Hz, 1H), 1.60 (s, 3H), and 1.38 (s, 3H); ^13^C NMR (100 MHz, CDCl_3_) δ 165.6, 155.8, 153.2, 149.3, 140.7, 140.1, 136.1, 132.2, 131.5, 128.7, 122.7, 120.5, 114.8, 90.8, 86.8, 84.1, 83.9, 52.6, 35.9, 33.7, 27.2, and 25.5. HRMS (ESI/TOF-Q) m/z: [M + H]^+^ calcd. for C_22_H_25_BrN_5_O_5_S, 550.0754; found, 550.0763.

Methyl 3-(((2′,3′-*O*-isopropylideneadenosyl)thio)methyl)-5-iodobenzoate (**4u**): Synthesized following general procedure P1 from **3** (0.28 g, 0.77 mmol) and methyl 5-(bromomethyl)-3-iodobenzoate (0.30 g, 0.84 mmol), yielded 0.43 g, 94% as a white foam. See the following: ^1^H NMR (400 MHz, CDCl_3_) δ 8.31 (s, 1H), 8.22 (t, *J =* 1.6 Hz, 1H), 7.89 (s, 1H), 7.86 (t, *J =* 1.6 Hz, 1H), 7.80 (t, *J =* 1.6 Hz, 1H), 6.04 (d, *J =* 2.2 Hz, 1H), 5.59 (s, 2H), 5.46 (dd, *J =* 6.5, 2.2 Hz, 1H), 5.03 (dd, *J =* 6.5, 3.5 Hz, 1H), 4.34 (td, *J =* 6.7, 3.5 Hz, 1H), 3.91 (s, 3H), 3.67 (s, 2H), 2.80 (dd, *J =* 13.7, 6.9 Hz, 1H), 2.70 (dd, *J =* 13.7, 6.5 Hz, 1H), 1.61 (s, 3H), and 1.39 (s, 3H); 13C NMR (100 MHz, CDCl3) δ 165.4, 155.8, 153.3, 149.3, 142.0, 140.7, 140.1, 137.4, 132.1, 129.3, 120.4, 114.8, 94.0, 90.8, 86.8, 84.1, 83.9, 52.6, 35.8, 33.7, 27.3, and 25.5. HRMS (ESI/TOF-Q) m/z: [M + H]^+^ calcd. for C_22_H_25_IN_5_O_5_S, 598.0627; found, 598.0616.

5′-((3-(Tetrazolyl)benzyl)thio)-5′-deoxy-2′,3′-*O*-isopropylideneadenosine (**4h**): A mixture of benzonitrile **4g** (0.08 g, 0.18 mmol), sodium azide (42 mg, 0.65 mmol), and ammonium chloride (32 mg, 0.60 mmol) in DMF (0.7 mL) was stirred in a closed vial at 110 °C for 16 h. The reaction mixture was cooled to room temperature and evaporated. The residue was filtered through a short pad of silica eluting with MeOH in DCM 10–20%, filtrate was evaporated under reduced pressure. The residue was purified by reverse-phase chromatography with eluent MeCN in water, and gradient 10–70% to obtain the title compound (0.07 g, 80%) as a white solid. See the following: ^1^H NMR (300 MHz, MeOH-*d*_4_) δ 8.26 (s, 1H), 8.15 (s, 1H), 8.03–7.97 (m, 1H), 7.89 (d, *J =* 7.6 Hz, 1H), 7.41 (t, *J =* 7.6 Hz, 1H), 7.37–7.30 (m, 1H), 6.15 (d, *J =* 2.4 Hz, 1H), 5.47 (dd, *J =* 6.4, 2.4 Hz, 1H), 5.00 (dd, *J =* 6.4, 3.2 Hz, 1H), 4.31 (td, *J =* 6.8, 3.2 Hz, 1H), 3.82 (s, 2H), 2.82 (dd, *J =* 13.9, 6.9 Hz, 1H), 2.77 (dd, *J =* 13.9, 6.7 Hz, 1H), 1.56 (s, 3H), and 1.35 (s, 3H); ^13^C NMR (100 MHz, MeOH-*d*_4_) δ 158.9, 157.2, 153.8, 150.1, 141.9, 141.2, 132.1, 130.3, 128.5, 127.4, 126.7, 120.6, 115.5, 91.6, 88.2, 85.1, 85.1, 37.0, 34.7, 27.3, and 25.5. HRMS (ESI/TOF-Q) m/z: [M + H]^+^ calcd. for C_21_H_24_N_9_O_3_S, 482.1717; found, 482.1733.

3-(((2′,3′-*O*-Isopropylideneadenosyl)thio)methyl)-*N*-methylbenzamide (**4l**): Methyl ester **4k** (0.21 g, 0.44 mmol) was stirred in THF (1 mL) and 2N NaOH (0.70 mL, 1.30 mmol) solution at 40 °C for 1 h, then at reflux for 30 min. The reaction mixture was concentrated, diluted with water (2 mL), and acidified with 1 N HCl to pH 3. The resulting precipitate was extracted with EtOAc (3 × 30 mL), washed with brine, dried over Na_2_SO_4_, and filtered and concentrated to obtain 3-(((2′,3′-*O*-isopropylideneadenosyl)thio)methyl) benzoic acid [22] (0.17 g, 87%) as a white solid. To a solution of 3-(((2′,3′-*O*-isopropylideneadenosyl)thio)methyl)benzoic acid (0.17 g, 0.37 mmol) in DMF (2 mL) was added HBTU (0.17 g, 0.44 mmol) and TEA (61 μl, 0.44 mmol) and the mixture was stirred for 1 h at room temperature. A solution of methylamine in THF (2 M, 0.22 mL, 0.44 mmol) was added to the reaction mixture and stirring continued for 18 h. The reaction mixture was evaporated under reduced pressure. The residue was supplemented with EtOAc (30 mL), washed with water, brine, dried over Na_2_SO_4_, and evaporated. The residue was purified by chromatography on silica gel on Biotage, eluent EtOAc:EtOH 3:1 in petroleum ether, gradient 40–100% to obtain the title compound (90 mg, 52%) as a white solid. See the following: ^1^H NMR (400 MHz, CDCl_3_) δ 8.25 (s, 1H), 7.91 (s, 1H), 7.64–7.56 (m, 2H), 7.34–7.28 (m, 2H), 6.43 (q, *J =* 4.8 Hz, 1H), 6.05 (d, *J =* 2.1 Hz, 1H), 5.94 (s, 2H), 5.44 (dd, *J =* 6.4, 2.1 Hz, 1H), 4.99 (dd, *J =* 6.4, 3.3 Hz, 1H), 4.33 (td, *J =* 6.7, 3.3 Hz, 1H), 3.70 (s, 2H), 2.99 (d, *J =* 4.8 Hz, 3H), 2.74 (dd, *J =* 13.7, 6.8 Hz, 1H), 2.64 (dd, *J =* 13.7, 6.6 Hz, 1H), 1.59 (s, 3H), and 1.41–1.35 (m, 3H); ^13^C NMR (100 MHz, CDCl_3_) δ 168.1, 155.6, 153.1, 149.2, 140.1, 138.4, 135.1, 131.7, 128.8, 127.2, 125.8, 120.2, 114.5, 90.7, 86.8, 84.1, 83.8, 36.4, 33.6, 27.1, 26.9, and 25.4. HRMS (ESI/TOF-Q) m/z: [M + H]^+^ calcd. for C_22_H_27_N_6_O_4_S, 471.1809; found, 471.1812.

Methyl 4-(((2′,3′-*O*-isopropylideneadenosyl)thio)methyl)-[1,1′-biphenyl]-2-carbo- xylate (**4r**): Synthesized following general procedure P2 from aryl bromide **4q** (0.10 g, 0.18 mmol) and phenylboronic acid (24 mg, 0.20 mmol), yielded 94 mg, 95%, white solid. See the following: ^1^H NMR (400 MHz, CDCl_3_) δ 8.33 (s, 1H), 7.92 (s, 1H), 7.72 (d, *J =* 1.9 Hz, 1H), 7.42–7.32 (m, 4H), 7.31–7.24 (m, 3H), 6.07 (d, *J =* 2.3 Hz, 1H), 5.57 (s, 2H), 5.48 (dd, *J =* 6.4, 2.3 Hz, 1H), 5.04 (dd, *J =* 6.5, 3.4 Hz, 1H), 4.39 (td, *J =* 6.7, 3.4 Hz, 1H), 3.76 (s, 2H), 3.63 (s, 3H), 2.82 (dd, *J =* 13.7, 7.0 Hz, 1H), 2.72 (dd, *J =* 13.7, 6.4 Hz, 1H), 1.61 (s, 3H), and 1.39 (s, 3H); ^13^C NMR (100 MHz, CDCl_3_) δ 168.9, 155.8, 153.3, 149.3, 141.5, 140.9, 140.1, 137.2, 131.7, 131.1, 131.1, 130.3, 128.4, 128.2, 127.4, 120.4, 114.7, 90.8, 86.8, 84.1, 83.9, 52.1, 36.0, 33.6, 27.2, and 25.5. HRMS (ESI/TOF-Q) m/z: [M + H]^+^ calcd. for C_28_H_30_N_5_O_5_S, 548.1962; found, 548.1971.

Methyl 5-(((2′,3′-*O*-isopropylideneadenosyl)thio)methyl)-4′-hydroxy-[1,1′-biphenyl]- 3-carboxylate (**4t**): Synthesized following general procedure P2 from aryl bromide **4s** (0.10 g, 0.18 mmol) and 4-hydroxyphenylboronic acid (28 mg, 0.20 mmol) yielded 84 mg, 82%, white solid. See the following: ^1^H NMR (400 MHz, CDCl_3_) δ 8.26 (s, 1H), 8.08 (t, *J =* 1.6 Hz, 1H), 7.91 (s, 1H), 7.85 (t, *J =* 1.6 Hz, 1H), 7.63 (t, *J =* 1.6 Hz, 1H), 7.50–7.43 (m, 2H), 6.94–6.86 (m, 2H), 6.56 (s, 1H), 6.04 (d, *J =* 2.3 Hz, 1H), 5.69 (s, 2H), 5.44 (dd, *J =* 6.5, 2.3 Hz, 1H), 5.01 (dd, *J =* 6.5, 3.4 Hz, 1H), 4.36 (td, *J =* 6.7, 3.4 Hz, 1H), 3.93 (s, 3H), 3.79 (s, 2H), 2.82 (dd, *J =* 13.7, 7.0 Hz, 1H), 2.72 (dd, *J =* 13.7, 6.4 Hz, 1H), 1.59 (s, 3H), and 1.37 (s, 3H); ^13^C NMR (100 MHz, CDCl_3_) δ 167.1, 156.7, 155.6, 153.1, 149.2, 141.6, 140.1, 138.9, 131.8, 131.5, 131.0, 128.5, 128.1, 126.8, 120.0, 116.2, 114.8, 90.9, 86.7, 84.1, 83.8, 52.4, 36.5, 33.6, 27.2, and 25.4. HRMS (ESI/TOF-Q) m/z: [M + H]^+^ calcd. for C_28_H_30_N_5_O_6_S, 564.1911; found, 564.1921.

Methyl 3-(((2′,3′-*O*-isopropylideneadenosyl)thio)methyl)-5-(3-hydroxyprop-1-yn-1- yl)benzoate (**4v**): A solution of aryl iodide **4u** (0.11 g, 0.18 mmol) and TEA (52 μl, 0.37 mmol) in acetonitrile (1 mL) was degassed by bubbling argon for 10 min, then propargyl alcohol (17 μL, 0.29 mmol), Pd(PPh_3_)_4_ (11 mg, 0.01 mmol), and CuI (5 mg, 0.03 mmol) were added under argon, and the vial was sealed. The reaction mixture was stirred at 35 °C for 16 h. The reaction mixture was evaporated under reduced pressure. The residue was purified by chromatography on silica gel on Biotage with eluent EtOAc:EtOH 3:1 in petroleum ether, and gradient 25–50% to obtain the title compound (90 mg, 93%) as a yellow solid. See the following: ^1^H NMR (400 MHz, CDCl_3_) δ 8.33 (s, 1H), 7.93 (t, *J =* 1.6 Hz, 1H), 7.91 (s, 1H), 7.89 (t, *J =* 1.6 Hz, 1H), 7.09 (t, *J =* 1.6 Hz, 1H), 6.08 (d, *J =* 2.1 Hz, 1H), 5.88 (s, 2H), 5.68 (s, 1H), 5.33 (dd, *J =* 6.4, 2.1 Hz, 1H), 4.94 (dd, *J =* 6.4, 3.9 Hz, 1H), 4.51 (s, 2H), 4.39 (ddd, *J =* 7.5, 5.6, 3.9 Hz, 1H), 3.90 (s, 3H), 3.71 (d, *J =* 14.1 Hz, 1H), 3.63 (d, *J =* 14.1 Hz, 1H), 2.73 (dd, *J =* 13.5, 5.6 Hz, 1H), 2.66 (dd, *J =* 13.5, 7.5 Hz, 1H), 1.60 (s, 3H), and 1.36 (s, 3H); ^13^C NMR (100 MHz, CDCl_3_) δ 166.2, 155.6, 153.3, 149.1, 139.9, 139.2, 136.0, 131.3, 131.0, 130.0, 123.1, 120.0, 114.8, 90.7, 89.3, 88.3, 88.4, 84.3, 84.0, 52.5, 51.0, 36.1, 33.3, 27.2, and 25.5. HRMS (ESI/TOF-Q) m/z: [M + H]^+^ calcd. for C_25_H_28_N_5_O_6_S, 526.1755; found, 526.1746.

4-(((Adenosyl)thio)methyl)benzoic acid (**5a**): Synthesized following general procedure P3 from **4a** (0.11 g, 0.21 mmol), yielded 36 mg, 40%. HPLC purity 91%. See the following: ^1^H NMR (400 MHz, DMSO-*d*_6_) δ 12.84 (s, 1H), 8.33 (s, 1H), 8.13 (s, 1H), 7.87–7.82 (m, 2H), 7.37–7.32 (m, 2H), 7.29 (s, 2H), 5.88 (d, *J =* 5.4 Hz, 1H), 5.49 (d, *J =* 5.9 Hz, 1H), 5.29 (d, *J =* 5.3 Hz, 1H), 4.72 (q, *J =* 5.5 Hz, 1H), 4.16 (q, *J =* 4.9 Hz, 1H), 4.00 (td, *J =* 7.0, 5.6, 4.1 Hz, 1H), 3.80 (s, 2H), 2.83 (dd, *J =* 13.9, 5.6 Hz, 1H), and 2.69 (dd, *J =* 13.9, 7.0 Hz, 1H); ^13^C NMR (100 MHz, DMSO-*d*_6_) δ 167.1, 156.1, 152.7, 149.4, 143.8, 140.0, 129.4, 129.3, 129.0, 119.2, 87.6, 83.7, 72.6, 72.5, 35.3, 33.3. HRMS (ESI/TOF-Q) m/z: [M + H]^+^ calcd. for C_18_H_20_N_5_O_5_S, 418.1180; found, 418.1194

5′-(((Benzo[d][1,3]dioxol-5-ylmethyl)thio)methyl)-5′-deoxyadenosine (**5b**): Synthesized following general procedure P3 from **4b** (82 mg, 0.18 mmol) yielded 64 mg, 86%. HPLC purity 99%. See the following: ^1^H NMR (400 MHz, DMSO-*d*_6_) δ 8.33 (s, 1H), 8.14 (s, 1H), 7.28 (s, 2H), 6.83 (d, *J =* 1.7 Hz, 1H), 6.76 (d, *J =* 7.9 Hz, 1H), 6.66 (dd, *J =* 7.9, 1.7 Hz, 1H), 5.97 (br s, 2H), 5.87 (d, *J =* 5.6 Hz, 1H), 5.48 (d, *J =* 6.0 Hz, 1H), 5.29 (d, *J =* 5.2 Hz, 1H), 4.73 (q, *J =* 5.5 Hz, 1H), 4.15 (q, *J =* 4.8 Hz, 1H), 4.02–3.97 (m, 1H), 3.65 (s, 2H), 2.82 (dd, *J =* 13.9, 5.9 Hz, 1H), and 2.67 (dd, *J =* 13.9, 6.8 Hz, 1H); ^13^C NMR (100 MHz, DMSO-*d*_6_) δ 156.1, 152.7, 149.4, 147.3, 146.1, 140.0, 132.2, 122.1, 119.2, 109.1, 107.9, 100.9, 87.6, 83.7, 72.6, 72.6, 35.5, and 33.2. HRMS (ESI/TOF-Q) m/z: [M + H]^+^ calcd. for C_18_H_20_N_5_O_5_S, 418.1180; found, 418.1198.

*N*-(3-(((Adenosyl)thio)methyl)phenyl)methanesulfonamide (**5c**): Synthesized following general procedure P3 from **4c** (0.11 g, 0.22 mmol) yielded 50 mg, 50%. HPLC purity 97%. See the following: ^1^H NMR (400 MHz, DMSO-*d*_6_) δ 9.72 (s, 1H), 8.33 (s, 1H), 8.13 (s, 1H), 7.28 (br s, 2H), 7.22 (t, *J =* 7.9 Hz, 1H), 7.15 (t, *J =* 1.7 Hz, 1H), 7.07 (ddd, *J =* 8.0, 2.1, 1.0 Hz, 1H), 6.96 (dt, *J =* 7.7, 1.4 Hz, 1H), 5.87 (d, *J =* 5.6 Hz, 1H), 5.49 (d, *J =* 6.0 Hz, 1H), 5.28 (d, *J =* 5.0 Hz, 1H), 4.73 (q, *J =* 5.6 Hz, 1H), 4.14 (td, *J =* 5.2, 3.8 Hz, 1H), 3.99 (td, *J =* 6.3, 3.8 Hz, 1H), 3.72 (s, 2H), 2.95 (s, 3H), 2.85 (dd, *J =* 13.8, 5.9 Hz, 1H), and 2.71 (dd, *J =* 13.8, 6.8 Hz, 1H); ^13^C NMR (100 MHz, DMSO-*d*_6_) δ 156.1, 152.7, 149.4, 139.9, 139.8, 138.5, 129.2, 124.4, 119.9, 119.2, 118.3, 87.5, 83.6, 72.6, 72.5, 39.2, 35.6, and 33.5. HRMS (ESI/TOF-Q) m/z: [M + H]^+^ calcd. for C_18_H_23_N_6_O_5_S_2_, 467.1166; found, 467.1188.

2-(3-(((Adenosyl)thio)methyl)phenyl)acetic acid (**5d**): Synthesized following general procedure P4 from **4d** (80 mg, 0.16 mmol), yielded 27 mg, 38%. HPLC purity 95%. See the following: ^1^H NMR (400 MHz, DMSO-*d*_6_) δ 12.25 (br s, 1H), 8.34 (s, 1H), 8.14 (s, 1H), 7.28 (br s, 2H), 7.21 (t, *J =* 7.5 Hz, 1H), 7.15 (t, *J =* 1.7 Hz, 1H), 7.14–7.07 (m, 2H), 5.88 (d, *J =* 5.5 Hz, 1H), 5.49 (br s, 1H), 5.30 (br s, 1H), 4.73 (t, *J =* 5.4 Hz, 1H), 4.16 (dd, *J =* 5.2, 4.0 Hz, 1H), 4.02 (ddd, *J =* 6.9, 5.8, 4.0 Hz, 1H), 3.72 (s, 2H), 3.52 (s, 2H), 2.84 (dd, *J =* 13.9, 5.8 Hz, 1H), and 2.71 (dd, *J =* 13.9, 6.9 Hz, 1H); ^13^C NMR (100 MHz, DMSO-*d*_6_) δ 172.7, 156.1, 152.7, 149.5, 139.9, 138.4, 135.2, 129.8, 128.2, 127.9, 127.2, 119.2, 87.6, 83.7, 72.6, 72.6, 40.6, 35.6, and 33.5. HRMS (ESI/TOF-Q) m/z: [M + H]^+^ calcd. for C_19_H_22_N_5_O_5_S, 432.1336; found, 432.1347.

5′-(((3-Hydroxy)benzyl)thio)-5′-deoxyadenosine (**5e**): Synthesized following general procedure P3 from **4e** (60 mg, 0.16 mmol), yielded 20 mg, 37%. HPLC purity 96%. See the following: ^1^H NMR (400 MHz, DMSO-*d*_6_) δ 9.34 (s, 1H), 8.33 (s, 1H), 8.14 (s, 1H), 7.28 (br s, 2H), 7.04 (t, *J =* 7.8 Hz, 1H), 6.69 (t, *J =* 1.9 Hz, 1H), 6.67–6.57 (m, 2H), 5.88 (d, *J =* 5.6 Hz, 1H), 5.48 (d, *J =* 6.0 Hz, 1H), 5.29 (d, *J =* 5.1 Hz, 1H), 4.74 (q, *J =* 5.6 Hz, 1H), 4.18–4.12 (m, 1H), 4.01 (td, *J =* 6.4, 3.8 Hz, 1H), 3.65 (s, 2H), 2.83 (dd, *J =* 13.8, 6.1 Hz, 1H), and 2.69 (dd, *J =* 13.8, 6.7 Hz, 1H); ^13^C NMR (100 MHz, DMSO-*d*_6_) δ 157.3, 156.1, 152.7, 149.5, 139.9, 139.7, 129.2, 119.6, 119.2, 115.7, 113.9, 87.5, 83.6, 72.6, 72.5, 35.7, and 33.4. HRMS (ESI/TOF-Q) m/z: [M + H]^+^ calcd. for C_17_H_20_N_5_O_4_S, 390.1231; found, 390.1240.

5′-(((3-Hydroxymethyl)benzyl)thio)-5′-deoxyadenosine (**5f**): Synthesized following general procedure P3 from **4f** (0.13 g, 0.23 mmol) yielded 55 mg, 60%. HPLC purity 98%. See the following: ^1^H NMR (400 MHz, DMSO-*d*_6_) 8.34 (s, 1H), 8.14 (s, 1H), 7.29 (br s, 2H), 7.24–7.19 (m, 1H), 7.16 (dt, *J =* 7.6, 1.4 Hz, 1H), 7.08 (dt, *J =* 7.3, 1.4 Hz, 1H), 5.88 (d, *J =* 5.5 Hz, 1H), 5.49 (d, *J =* 6.0 Hz, 1H), 5.30 (d, *J =* 5.2 Hz, 1H), 5.16 (t, *J =* 5.3 Hz, 1H), 4.73 (q, *J =* 5.5 Hz, 1H), 4.45 (d, *J =* 5.3 Hz, 2H), 4.18–4.14 (m, 1H), 4.02 (td, *J =* 6.3, 3.9 Hz, 1H), 3.73 (s, 2H), 2.84 (dd, *J =* 13.9, 5.8 Hz, 1H), and 2.70 (dd, *J =* 13.9, 6.8 Hz, 1H); ^13^C NMR (100 MHz, DMSO-*d*_6_) δ 156.1, 152.7, 149.5, 142.7, 140.0, 138.2, 128.1, 127.2, 126.9, 125.0, 119.2, 87.6, 83.7, 72.6, 72.6, 62.8, 35.8, and 33.5. HRMS (ESI/TOF-Q) m/z: [M + H]^+^ calcd. for C_18_H_22_N_5_O_4_S, 404.1387; found, 404.1396.

3-(((Adenosyl)thio)methyl)benzonitrile (**5g**): Synthesized following general procedure P3 from **4g** (96 mg, 0.22 mmol), yielded 73 mg, 84%. HPLC purity 95%. See the following: ^1^H NMR (300 MHz, DMSO-*d*_6_) δ 8.32 (s, 1H), 8.13 (s, 1H), 7.74 (t, *J =* 1.5 Hz, 1H), 7.69 (dt, *J =* 7.6, 1.5 Hz, 1H), 7.58 (dt, *J =* 7.8, 1.5 Hz, 1H), 7.47 (t, *J =* 7.7 Hz, 1H), 7.28 (s, 2H), 5.87 (d, *J =* 5.4 Hz, 1H), 5.49 (d, *J =* 5.9 Hz, 1H), 5.29 (d, *J =* 5.2 Hz, 1H), 4.72 (q, *J =* 5.4 Hz, 1H), 4.21–4.10 (m, 1H), 4.06–3.93 (m, 1H), 3.80 (s, 2H), 2.84 (dd, *J =* 13.9, 5.7 Hz, 1H), and 2.71 (dd, *J =* 13.9, 7.0 Hz, 1H); 13C NMR (100 MHz, DMSO-*d*_6_) δ 156.1, 152.7, 149.4, 140.6, 140.0, 133.8, 132.3, 130.7, 129.6, 119.2, 118.7, 111.3, 87.7, 83.6, 72.6, 72.5, 34.7, and 33.4. HRMS (ESI/TOF-Q) m/z: [M + H]^+^ calcd. for C_18_H_19_N_6_O_3_S, 399.1234; found, 399.1259.

5′-((3-(Tetrazolyl)benzyl)thio)-5′-deoxyadenosine (**5h**): Synthesized following general procedure P3 from **4h** (70 mg, 0.15 mmol) yielded 38 mg, 59%. HPLC purity 96%. See the following: ^1^H NMR (400 MHz, DMSO-*d*_6_) δ 8.33 (s, 1H), 8.12 (s, 1H), 8.01 (t, *J =* 1.5 Hz, 1H), 7.89 (dt, *J =* 7.7, 1.5 Hz, 1H), 7.49 (t, *J =* 7.7 Hz, 1H), 7.43 (dt, *J =* 7.7, 1.5 Hz, 1H), 7.28 (s, 2H), 5.88 (d, *J =* 5.5 Hz, 1H), 5.49 (d, *J =* 4.7 Hz, 1H), 5.30 (br s, 1H), 4.77–4.71 (m, 1H), 4.17 (t, *J =* 4.8 Hz, 1H), 4.03 (ddd, *J =* 7.0, 5.8, 4.0 Hz, 1H), 3.86 (s, 2H), 2.88 (dd, *J =* 13.8, 5.8 Hz, 1H), and 2.75 (dd, *J =* 13.8, 7.0 Hz, 1H); ^13^C NMR (100 MHz, DMSO-*d*_6_) δ 156.1, 155.5, 152.6, 149.4, 140.0, 140.0, 131.6, 129.4, 127.3, 125.5, 124.6, 119.2, 87.6, 83.6, 72.6, 72.5, 35.3, and 33.5. HRMS (ESI/TOF-Q) m/z: [M + H]^+^ calcd. for C_18_H_20_N_9_O_3_S, 442.1404; found, 442.1411.

5′-((3-(Methylsulfonyl)benzyl)thio)-5′-deoxyadenosine (**5i**): Synthesized following general procedure P3 from **4i** (84 mg, 0.17 mmol) yielded 58 mg, 75%. HPLC purity 97%. See the following: ^1^H NMR (400 MHz, CDCl_3_) 8.33 (s, 1H), 8.13 (s, 1H), 7.86 (s, 1H), 7.78 (d, *J =* 7.2 Hz, 1H), 7.62–7.50 (m, 2H), 7.28 (br s, 2H), 5.87 (d, *J =* 5.3 Hz, 1H), 5.50 (d, *J =* 5.8 Hz, 1H), 5.29 (d, *J =* 5.1 Hz, 1H), 4.72 (q, *J =* 5.3 Hz, 1H), 4.15 (q, *J =* 4.7 Hz, 1H), 4.03–3.97 (m, 1H), 3.87 (s, 2H), 3.19 (s, 3H), 2.86 (dd, *J =* 13.8, 5.6 Hz, 1H), and 2.74 (dd, *J =* 13.8, 6.8 Hz, 1H); ^13^C NMR (100 MHz, DMSO-*d*_6_) δ 156.1, 152.7, 149.4, 141.0, 140.5, 140.0, 133.9, 129.5, 126.9, 125.4, 119.2, 87.6, 83.7, 72.6, 72.5, 43.5, 35.0, and 33.5. HRMS (ESI/TOF-Q) m/z: [M + H]^+^ calcd. for C_18_H_22_N_5_O_5_S_2_, 452.1057; found 452.1060.

3-(((Adenosyl)thio)methyl)-*N*-methylbenzenesulfonamide (**5j**): Synthesized following general procedure P3 from **4j** (0.10 g, 0.20 mmol), yielded 73 mg, 79%. HPLC purity 93%. See the following: ^1^H NMR (400 MHz, DMSO-*d*_6_) δ 8.33 (s, 1H), 8.13 (s, 1H), 7.72 (s, 1H), 7.67–7.58 (m, 1H), 7.54–7.47 (m, 2H), 7.42 (q, *J =* 5.0 Hz, 1H), 7.28 (br s, 2H), 5.87 (d, *J =* 5.5 Hz, 1H), 5.51 (d, *J =* 5.1 Hz, 1H), 5.30 (d, *J =* 4.2 Hz, 1H), 4.73 (q, *J =* 4.9 Hz, 1H), 4.18–4.13 (m, 1H), 4.00 (td, *J =* 6.4, 4.2 Hz, 1H), 3.85 (s, 2H), 2.85 (dd, *J =* 13.9, 5.8 Hz, 1H), 2.72 (dd, *J =* 13.9, 7.0 Hz, 1H), and 2.38 (t, *J =* 5.0 Hz, 3H); ^13^C NMR (100 MHz, DMSO-*d_6_*) δ 156.1, 152.7, 149.4, 140.1, 140.0, 139.4, 132.8, 129.3, 126.9, 125.1, 119.2, 87.6, 83.6, 72.6, 72.5, 35.1, 33.5, and 28.7. HRMS (ESI/TOF-Q) m/z: [M + H]^+^ calcd. for C_18_H_23_N_6_O_5_S, 467.1166; found, 467.1180.

Methyl 3-(((adenosyl)thio)methyl)benzoate (**5k**): Synthesized following general procedure P3 from **4k** (73 mg, 0.15 mmol), yielded 44 mg, 66%. HPLC purity 96%. See the following: ^1^H NMR (400 MHz, DMSO-*d*_6_) δ 8.32 (s, 1H), 8.12 (s, 1H), 7.89 (t, *J =* 1.5 Hz, 1H), 7.81 (dt, *J =* 7.7, 1.5 Hz, 1H), 7.50 (dt, *J =* 7.7, 1.5 Hz, 1H), 7.41 (t, *J =* 7.7 Hz, 1H), 7.28 (s, 2H), 5.87 (d, *J =* 5.5 Hz, 1H), 5.48 (d, *J =* 6.0 Hz, 1H), 5.29 (d, *J =* 5.1 Hz, 1H), 4.72 (q, *J =* 5.5 Hz, 1H), 4.15 (td, *J =* 5.3, 3.9 Hz, 1H), 4.00 (ddd, *J =* 7.0, 5.8, 3.9 Hz, 1H), 3.84 (s, 3H), 3.83 (s, 2H), 2.84 (dd, *J =* 13.8, 5.8 Hz, 1H), and 2.68 (dd, *J =* 13.8, 7.0 Hz, 1H); ^13^C NMR (100 MHz, DMSO-*d*_6_) δ 166.1, 163.2, 156.1, 152.7, 149.4, 140.0, 139.4, 133.7, 129.8, 129.5, 128.8, 127.7, 119.2, 87.6, 83.7, 72.5, 52.2, 35.1, and 33.4. HRMS (ESI/TOF-Q) m/z: [M + H]^+^ calcd. for C_19_H_22_N_5_O_5_S, 432.1336; found, 432.1338.

3-(((Adenosyl)thio)methyl)-*N*-methylbenzamide (**5l**): Synthesized following general procedure P3 from **4l** (90 mg, 0.19 mmol), yielded 24 mg, 29%. HPLC purity 98%. See the following: ^1^H NMR (400 MHz, DMSO-*d*_6_) δ 8.39 (q, *J =* 4.5 Hz, 1H), 8.33 (s, 1H), 8.13 (s, 1H), 7.76 (t, *J =* 1.5 Hz, 1H), 7.67 (dt, *J =* 6.8, 1.8 Hz, 1H), 7.38–7.31 (m, 2H), 7.28 (br s, 2H), 5.88 (d, *J =* 5.5 Hz, 1H), 5.49 (d, *J =* 5.9 Hz, 1H), 5.30 (d, *J =* 5.2 Hz, 1H), 4.73 (q, *J =* 5.5 Hz, 1H), 4.18–4.13 (m, 1H), 4.01 (td, *J =* 6.4, 4.0 Hz, 1H), 3.79 (s, 2H), 2.84 (dd, *J =* 13.9, 5.8 Hz, 1H), 2.77 (d, *J =* 4.5 Hz, 3H), and 2.71 (dd, *J =* 13.9, 7.0 Hz, 1H); ^13^C NMR (100 MHz, DMSO-*d*_6_) δ 166.9, 156.5, 153.1, 149.9, 140.4, 139.2, 135.2, 131.8, 128.7, 128.1, 125.9, 119.6, 88.0, 84.1, 73.1, 73.0, 36.0, 33.9, 26.7, and 26.7. HRMS (ESI/TOF-Q) m/z: [M + H]^+^ calcd. for C_19_H_23_N_6_O_4_S, 431.1496; found, 431.1521.

5-(((Adenosyl)thio)methyl)-2-chlorobenzoic acid (**5m**): Synthesized following general procedure P4 from **4m** (90 mg, 0.18 mmol), yielded 57 mg, 71%. HPLC purity 97%. See the following: ^1^H NMR (400 MHz, DMSO-*d*_6_) δ 13.40 (br s, 1H), 8.33 (s, 1H), 8.13 (s, 1H), 7.72 (d, *J =* 2.2 Hz, 1H), 7.42 (d, *J =* 8.2 Hz, 1H), 7.36 (dd, *J =* 8.2, 2.2 Hz, 1H), 5.87 (d, *J =* 5.5 Hz, 1H), 5.49 (d, *J =* 5.8 Hz, 1H), 5.30 (d, *J =* 4.2 Hz, 1H), 4.72 (q, *J =* 5.4 Hz, 1H), 4.18–4.13 (m, 1H), 4.00 (ddd, *J =* 7.0, 5.8, 4.1 Hz, 1H), 3.78 (s, 2H), 2.84 (dd, *J =* 13.8, 5.8 Hz, 1H), and 2.71 (dd, *J =* 13.8, 7.0 Hz, 1H); ^13^C NMR (100 MHz, DMSO-*d*_6_) δ 166.6, 156.1, 152.7, 149.4, 140.0, 138.1, 132.9, 131.3, 131.1, 130.5, 130.0, 119.2, 87.6, 83.6, 72.6, 72.5, 34.4, and 33.4. HRMS (ESI/TOF-Q) m/z: [M + H]^+^ calcd. for C_18_H_19_ClN_5_O_5_S, 452.0790; found, 452.0800.

5-(((Adenosyl)thio)methyl)-2-fluorobenzoic acid (**5n**): Synthesized following general procedure P4 from **4n** (74 mg, 0.15 mmol), yielded 43 mg, 65%. HPLC purity 96%. See the following: ^1^H NMR (400 MHz, DMSO-*d*_6_) δ 13.22 (br s, 1H), 8.33 (s, 1H), 8.13 (s, 1H), 7.79 (dd, *J =* 7.1, 2.4 Hz, 1H), 7.46 (ddd, *J =* 8.4, 4.6, 2.4 Hz, 1H), 7.28 (s, 2H), 7.18 (dd, *J =* 10.8, 8.4 Hz, 1H), 5.87 (d, *J =* 5.5 Hz, 1H), 5.49 (d, *J =* 4.6 Hz, 1H), 5.30 (br s, 1H), 4.73 (q, *J =* 4.8 Hz, 1H), 4.15 (t, *J =* 4.4 Hz, 1H), 4.00 (ddd, *J =* 6.8, 5.9, 4.0 Hz, 1H), 3.79 (s, 2H), 2.84 (dd, *J =* 13.8, 5.9 Hz, 1H), and 2.70 (dd, *J =* 13.8, 6.8 Hz, 1H); ^13^C NMR (100 MHz, DMSO*-d*_6_) δ 165.0 (d, *J*_CF_ = 3.0 Hz), 160.0 (d, *J*_CF_ = 256.3 Hz), 156.1, 152.7, 149.4, 140.0, 135.0 (d, *J*_CF_ = 3.7 Hz), 134.9 (d, *J*_CF_ = 8.9 Hz), 132.0, 119.3, 119.2, 116.9 (d, *J*_CF_ = 22.7 Hz), 87.6, 83.6, 72.6, 72.5, 34.4, and 33.4. HRMS (ESI/TOF-Q) m/z: [M + H]^+^ calcd. for C_18_H_19_FN_5_O_5_S, 436.1085; found, 436.1094.

3-(((Adenosyl)thio)methyl)-5-chlorobenzoic acid (**5o**): Synthesized following general procedure P4 from **4o** (74 mg, 0.15 mmol), yielded 26 mg, 39%. HPLC purity 93%. See the following: ^1^H NMR (400 MHz, DMSO-*d*_6_) δ 13.30 (br s, 1H), 8.32 (s, 1H), 8.12 (s, 1H), 7.84 (t, *J =* 1.7 Hz, 1H), 7.75 (t, *J =* 1.7 Hz, 1H), 7.58 (t, *J =* 1.7 Hz, 1H), 7.27 (s, 2H), 5.87 (d, *J =* 5.5 Hz, 1H), 5.48 (d, *J =* 5.0 Hz, 1H), 5.30 (s, 1H), 4.73 (q, *J =* 5.0 Hz, 1H), 4.15 (t, *J =* 4.5 Hz, 1H), 4.00 (td, *J =* 6.4, 4.0 Hz, 1H), 3.84 (s, 2H), 2.86 (dd, *J =* 13.8, 5.8 Hz, 1H), and 2.73 (dd, *J =* 13.8, 7.0 Hz, 1H); ^13^C NMR (100 MHz, DMSO-*d*_6_) δ 166.0, 156.1, 152.7, 149.4, 142.0, 140.0, 133.1, 133.1, 132.8, 128.4, 127.3, 119.3, 87.6, 83.5, 72.6, 72.5, 34.6, and 33.4. HRMS (ESI/TOF-Q) m/z: [M + H]^+^ calcd. for C_18_H_19_ClN_5_O_5_S, 452.0790; found, 452.0807.

5-(((Adenosyl)thio)methyl)-[1,1′-biphenyl]-3-carboxylic acid (**5p**): Synthesized following general procedure P4 from **4p** (98 mg, 0.18 mmol), yielded 60 mg, 68%. HPLC purity 94%. See the following: ^1^H NMR (400 MHz, DMSO-*d*_6_) δ 13.10 (br s, 1H), 8.34 (s, 1H), 8.11 (s, 1H), 8.03 (t, *J =* 1.6 Hz, 1H), 7.89 (t, *J =* 1.6 Hz, 1H), 7.78 (t, *J =* 1.6 Hz, 1H), 7.69–7.63 (m, 2H), 7.51–7.44 (m, 2H), 7.43–7.37 (m, 1H), 7.27 (br s, 2H), 5.88 (d, *J =* 5.7 Hz, 1H), 5.51 (br s, 2H), 4.74 (t, *J =* 5.4 Hz, 1H), 4.17 (dd, *J =* 5.1, 4.0 Hz, 1H), 4.04 (td, *J =* 6.3, 4.0 Hz, 1H), 3.90 (s, 2H), 2.90 (dd, *J =* 13.8, 5.9 Hz, 1H), and 2.77 (dd, *J =* 13.8, 6.7 Hz, 1H); ^13^C NMR (100 MHz, DMSO-*d*_6_) δ 167.4, 156.1, 152.7, 149.4, 140.5, 139.9, 139.9, 139.2, 132.7, 131.2, 129.1, 128.7, 127.9, 126.8, 125.9, 119.2, 87.5, 83.4, 72.6, 72.6, 35.3, and 33.6. HRMS (ESI/TOF-Q) m/z: [M + H]^+^ calcd. for C_24_H_24_N_5_O_5_S, 494.1493; found, 494.1499.

4-(((Adenosyl)thio)methyl)-[1,1′-biphenyl]-2-carboxylic acid (**5r**): Synthesized following general procedure P4 from **4r** (90 mg, 0.16 mmol), yielded 25 mg, 31%. HPLC purity 95%. See the following: 1H NMR (400 MHz, DMSO-*d_6_*) 12.78 (br s, 1H), 8.35 (s, 1H), 8.14 (s, 1H), 7.64 (d, *J =* 1.9 Hz, 1H), 7.42–7.30 (m, 6H), 7.28 (br s, 2H), 7.26 (d, *J =* 7.9 Hz, 1H), 5.89 (d, *J =* 5.5 Hz, 1H), 5.51 (br s, 1H), 5.35 (br s, 1H), 4.74 (t, *J =* 5.2 Hz, 1H), 4.19 (dd, *J =* 4.0, 4.9 Hz, 1H), 4.05 (td, *J =* 6.3, 4.0 Hz, 1H), 3.83 (s, 2H), 2.89 (dd, *J =* 13.9, 5.7 Hz, 1H), and 2.75 (dd, *J =* 13.9, 6.9 Hz, 1H); ^13^C NMR (100 MHz, DMSO-*d_6_*) 169.8, 156.2, 152.8, 149.5, 140.6, 140.1, 139.5, 137.9, 132.7, 131.2, 130.6, 129.5, 128.4, 128.2, 127.2, 119.3, 87.7, 83.8, 72.7, 72.7, 35.0, and 33.6. HRMS (ESI/TOF-Q) m/z: [M + H]^+^ calcd. for C_24_H_24_N_5_O_5_S, 494.1493; found, 494.1511.

5-(((Adenosyl)thio)methyl)-4′-hydroxy-[1,1′-biphenyl]-3-carboxylic acid (**5t**): Synthesized following general procedure P4 from **4t** (80 mg, 0.14 mmol), yielded 32 mg, 44%. HPLC purity 91%. See the following: ^1^H NMR (400 MHz, DMSO-*d_6_*) 13.00 (br s, 1H), 9.64 (s, 1H), 8.33 (s, 1H), 8.11 (s, 1H), 7.96 (t, *J =* 1.6 Hz, 1H), 7.80 (d, *J =* 1.6 Hz, 1H), 7.72 (t, *J =* 1.6 Hz, 1H), 7.52–7.46 (m, 2H), 7.26 (br s, 2H), 6.90–6.82 (m, 2H), 5.88 (d, *J =* 5.7 Hz, 1H), 5.49 (d, *J =* 5.4 Hz, 1H), 5.32 (br s, 1H), 4.73 (q, *J =* 5.1 Hz, 1H), 4.16 (t, *J =* 4.4 Hz, 1H), 4.03 (td, *J =* 6.4, 3.8 Hz, 1H), 3.87 (s, 2H), 2.88 (dd, *J =* 13.8, 6.0 Hz, 1H), and 2.75 (dd, *J =* 13.8, 6.8 Hz, 1H); ^13^C NMR (100 MHz, DMSO-*d_6_*) 167.3, 157.6, 156.1, 152.7, 149.5, 140.6, 140.0, 139.8, 131.7, 130.8, 129.8, 127.9, 127.7, 125.2, 119.2, 115.9, 87.6, 83.5, 72.6 (2C), 35.4, and 33.6. HRMS (ESI/TOF-Q) m/z: [M + H]^+^ calcd. for C_24_H_24_N_5_O_6_S, 510.1442; found, 510.1443.

3-(((Adenosyl)thio)methyl)-5-(3-hydroxyprop-1-yn-1-yl)benzoic acid (**5v**): Synthesized following general procedure P4 from **4v** (90 mg, 0.17 mmol), yielded 19 mg, 24%. HPLC purity 99%. See the following: ^1^H NMR (400 MHz, DMSO-*d_6_*) 8.33 (s, 1H), 8.13 (s, 1H), 7.85 (s, 1H), 7.76 (s, 1H), 7.49 (s, 1H), 7.27 (br s, 2H), 5.87 (d, *J =* 5.6 Hz, 1H), 5.43 (br s, 2H), 4.73 (t, *J =* 5.4 Hz, 1H), 4.30 (s, 2H), 4.14 (dd, *J =* 3.8, 5.1 Hz, 1H), 4.00 (td, *J =* 6.4, 3.8 Hz, 1H), 3.80 (s, 2H), 2.84 (dd, *J =* 13.7, 6.0 Hz, 1H), and 2.70 (dd, *J =* 13.7, 6.8 Hz, 1H); ^13^C NMR (100 MHz, DMSO-*d_6_*) 167.0, 156.1, 152.7, 149.5, 140.0, 139.8, 134.8, 133.6, 130.4, 129.8, 122.6, 119.3, 90.6, 87.6, 83.5, 82.9, 72.6 (2 C), 49.5, 34.9, and 33.5. HRMS (ESI/TOF-Q) m/z: [M + H]^+^ calcd. for C_21_H_22_N_5_O_6_S, 471.1285; found, 471.1301.

2-Chloro-5-((adenosyl)thio)benzoic acid (**10a**): Sodium methoxide (5.4 M in MeOH, 0.11 mL, 0.60 mmol) was added dropwise to a solution of 2-chloro-5-mercaptobenzoic acid (58 mg, 0.31 mmol) in DMF (0.7 mL) under argon at −30 °C, the reaction mixture was allowed to warm up to 0 °C while stirring (30 min). The reaction mixture was cooled to −30 °C and a solution of 5′-chloro-5′-deoxyadenosine (**6**) [24] (80 mg, 0.28 mmol) in DMF (0.5 mL) was added dropwise. The mixture was allowed to warm to room temperature, stirred for 72 h, and evaporated. The residue was treated with water (2 mL), cooled in the ice bath and neutralized to pH 4 with 1 M HCl. The precipitate was filtered through a nylon filter, washed with water (2 × 2 mL), and dried in vacuo. The residue was dissolved in DMF (0.2 mL) and purified by reverse-phase chromatography with eluent MeCN in 0.1% HCOOH, and gradient 10–50% to obtain the title compound (37 mg, 30%) as a white solid. HPLC purity 96%. See the following: ^1^H NMR (400 MHz, DMSO-*d*_6_) δ 8.34 (s, 1H), 8.14 (s, 1H), 7.67 (d, *J =* 2.4 Hz, 1H), 7.47 (dd, *J =* 8.5, 2.4 Hz, 1H), 7.42 (d, *J =* 8.5 Hz, 1H), 7.29 (br s, 2H), 5.89 (d, *J =* 5.8 Hz, 1H), 5.53 (d, *J =* 4.8 Hz, 1H), 5.40 (s, 1H), 4.84–4.79 (m, 1H), 4.19 (t, *J =* 4.1 Hz, 1H), 4.00 (ddd, *J =* 7.1, 5.7, 3.6 Hz, 1H), 3.47 (dd, *J =* 13.7, 5.6 Hz, 1H), and 3.38 (d, overlapped with water, 1H); ^13^C NMR (100 MHz, DMSO-*d*_6_) δ 166.3, 156.1, 152.7, 149.5, 140.0, 135.6, 132.3, 131.6, 131.0, 129.5, 128.7, 119.2, 87.6, 82.6, 72.7, 72.6, and 35.1. HRMS (ESI/TOF-Q) m/z: [M + H]^+^ calcd. for C_17_H_17_ClN_5_O_5_S, 438.0633; found, 438.0648.

4-Chloro-5-((adenosyl)thio)benzoic acid (**10b**): Synthesized following the procedure for **10a** from 4-chloro-3-mercaptobenzoic acid (63 mg, 0.33 mmol) and 5′-chloro-5′-deoxyadenosine (**6**), yielded 46 mg, 38%. HPLC purity 99%. See the following: ^1^H NMR (400 MHz, DMSO-*d*_6_) δ 13.29 (br s, 1H), 8.34 (s, 1H), 8.14 (s, 1H), 7.92 (d, *J =* 1.9 Hz, 1H), 7.70 (dd, *J =* 8.3, 1.9 Hz, 1H), 7.58 (d, *J =* 8.3 Hz, 1H), 7.29 (br s, 2H), 5.92 (d, *J =* 5.8 Hz, 1H), 5.54 (d, *J =* 6.0 Hz, 1H), 5.45 (d, *J =* 4.9 Hz, 1H), 4.85 (q, *J =* 5.4 Hz, 1H), 4.29–4.25 (m, 1H), 4.08 (ddd, *J =* 7.3, 5.7, 3.5 Hz, 1H), 3.54 (dd, *J =* 13.2, 5.7 Hz, 1H), and 3.47 (dd, *J =* 13.2, 7.3 Hz, 1H); ^13^C NMR (100 MHz, DMSO-*d*_6_) δ 166.3, 156.0, 152.6, 149.4, 140.0, 136.3, 135.5, 130.4, 129.8, 127.5, 127.1, 119.2, 87.7, 82.2, 72.9, 72.6, and 34.0. HRMS (ESI/TOF-Q) m/z: [M + H]^+^ calcd. for C_17_H_17_ClN_5_O_5_S, 438.0633; found, 438.0632.

2-Bromo-5-((adenosyl)thio)benzoic acid (**10c**): Potassium *terc*-butoxide (1 M solution in THF, 0.77 mL, 0.77 mmol) was added dropwise to an ice-cold solution of 2-bromo-5-mercaptobenzoic acid (0.09 g, 0.39 mmol) in DMF (1 mL) under argon atmosphere, then 5′-chloro-5′-deoxyadenosine (**6**) (0.10 g, 0.35 mmol) solution in DMF (1 mL) was added dropwise. The reaction mixture was allowed to warm to room temperature and stirred for 19 h. The reaction mixture was evaporated under reduced pressure. The residue was diluted with water (5 mL) and acidified with 1 M HCl to pH 3, then evaporated under reduced pressure. The residue was purified by reverse-phase chromatography with eluent MeCN in 0.1% HCOOH, and gradient 0–50% to obtain the title compound (0.10 g, 60%) as a white solid. HPLC purity 99%. See the following: ^1^H NMR (400 MHz, DMSO-*d*_6_) δ 13.45 (br s, 1H), 8.34 (s, 1H), 8.15 (s, 1H), 7.64 (d, *J =* 2.4 Hz, 1H), 7.59 (d, *J =* 8.5 Hz, 1H), 7.39 (dd, *J =* 8.5, 2.4 Hz, 1H), 7.30 (br s, 2H), 5.89 (d, *J =* 5.9 Hz, 1H), 5.53 (d, *J =* 6.1 Hz, 1H), 5.39 (d, *J =* 4.8 Hz, 1H), 4.81 (q, *J =* 5.5 Hz, 1H), 4.22–4.17 (m, 1H), 4.00 (ddd, *J =* 7.2, 5.8, 3.4 Hz, 1H), 3.47 (dd, *J =* 13.8, 5.8 Hz, 1H), and 3.47 (dd, *J =* 13.8, 7.2 Hz, 1H); ^13^C NMR (100 MHz, DMSO-*d*_6_) δ 166.9, 156.1, 152.6, 149.4, 140.0, 136.3, 134.4, 134.1, 131.4, 129.2, 119.2, 116.7, 87.5, 82.6, 72.7, 72.6, and 34.9. HRMS (ESI/TOF-Q) m/z: [M + H]^+^ calcd. for C_17_H_17_BrN_5_O_5_S, 482.0128; found, 482.0139.

Methyl 2-methoxy-5-((2′,3′-O-isopropylideneadenosyl)thio)benzoate (**8a**): To a solution of methyl 5-mercapto-2-methoxybenzoate (0.05 g, 0.25 mmol) in dry THF (2 mL) under argon cooling in the ice bath was added potassium *terc*-butoxide solution in THF (1 M, 0.28 mL, 0.28 mmol), then after 5 min 5′-chloro-5′-deoxy-2′,3′-*O*-isopropylidenadenosine (**7**) [25] (0.07 g, 0.21 mmol) was added in one portion, then to the resulting suspension was added DMF (dry, 0.1 mL). The reaction mixture was stirred at room temperature for 15 h. The reaction mixture was quenched by saturated NH_4_Cl solution, extracted with EtOAc (2 × 15 mL), combined organic layers were washed with brine, dried over Na_2_SO_4_, filtered, and evaporated. The residue was chromatographed on silica gel on Biotage, eluent EtOAc:EtOH 3:1 in petroleum ether, gradient 20–50% to obtain the title compound (74 mg, 71%) as a white solid. See the following: ^1^H NMR (400 MHz, CDCl_3_) δ 8.33 (s, 1H), 7.86 (d, *J =* 2.5 Hz, 1H), 7.85 (s, 1H), 7.50 (dd, *J =* 8.7, 2.5 Hz, 1H), 6.85 (d, *J =* 8.7 Hz, 1H), 6.03 (d, *J =* 2.1 Hz, 1H), 5.58 (br s, 2H), 5.52 (dd, *J =* 6.4, 2.1 Hz, 1H), 5.09 (dd, *J =* 6.3, 3.0 Hz, 1H), 4.34 (ddd, *J =* 7.7, 6.5, 3.0 Hz, 1H), 3.89 (s, 3H), 3.88 (s, 3H), 3.17 (dd, *J =* 13.7, 7.7 Hz, 1H), 3.10 (dd, *J =* 13.7, 6.5 Hz, 1H), 1.58 (s, 3H), and 1.39 (s, 3H); ^13^C NMR (100 MHz, CDCl_3_) δ 165.9, 158.8, 155.7, 153.2, 149.2, 140.3, 137.8, 135.9, 125.2, 120.8, 120.5, 114.5, 112.8, 91.3, 86.5, 84.2, 84.0, 56.3, 52.3, 38.4, 27.2, and 25.5. HRMS (ESI/TOF-Q) m/z: [M + H]^+^ calcd. for C_22_H_26_N_5_O_6_S, 488.1598; found, 488.1613.

Methyl 2-bromo-5-((2′,3′-*O*-isopropylideneadenosyl)thio)benzoate (**8b**): Synthesized following procedure for **8a** from methyl 2-bromo-5-mercaptobenzoate (0.18 g, 0.74 mmol) and 5′-chloro-5′-deoxy-2′,3′-*O*-isopropylidenadenosine (**7**) (0.20 g, 0.61 mmol), yielded 0.21 g, calculated yield is 48%, contains 20% of **7** as an impurity based on LCMS. See the following: ^1^H NMR (300 MHz, CDCl_3_) δ 8.33 (s, 1H), 7.84 (s, 1H), 7.73 (d, *J =* 2.4 Hz, 1H), 7.48 (d, *J =* 8.4 Hz, 1H), 7.23 (dd, *J =* 8.4, 2.4 Hz, 1H), 6.04 (d, *J =* 2.0 Hz, 1H), 5.69 (br s, 2H), 5.53 (dd, *J =* 6.3, 2.0 Hz, 1H), 5.11 (dd, *J =* 6.3, 3.1 Hz, 1H), 4.37 (td, *J =* 7.0, 3.1 Hz, 1H), 3.91 (s, 3H), 3.31 (dd, *J =* 13.7, 7.5 Hz, 1H), 3.21 (dd, *J =* 13.7, 6.5 Hz, 1H), 1.58 (s, 3H), and 1.39 (s, 3H). HRMS (ESI/TOF-Q) m/z: [M + H]^+^ calcd. for C_21_H_23_BrN_5_O_5_S, 536.0598; found, 536.0611.

Methyl 2-bromo-4-((2′,3′-*O*-isopropylideneadenosyl)thio)benzoate (**8c**): Synthesized following procedure for **8a** from methyl 2-bromo-4-mercaptobenzoate (0.17 g, 0.71 mmol) and 5′-chloro-5′-deoxy-2′,3′-*O*-isopropylidenadenosine (**7**) (0.20 g, 0.61 mmol), yielded 0.27 g, calculated yield is 82%, contains 10% of **7** as an impurity based on LCMS as a white solid. See the following: ^1^H NMR (300 MHz, CDCl_3_) δ 8.34 (s, 1H), 7.85 (s, 1H), 7.66 (d, *J =* 8.3 Hz, 1H), 7.53 (d, *J =* 1.9 Hz, 1H), 7.18 (dd, *J =* 8.3, 1.9 Hz, 1H), 6.06 (d, *J =* 2.0 Hz, 1H), 5.98 (br s, 2H), 5.53 (dd, *J =* 6.3, 2.0 Hz, 1H), 5.13 (dd, *J =* 6.3, 3.2 Hz, 1H), 4.39 (td, *J =* 6.9, 3.2 Hz, 1H), 3.89 (s, 3H), 3.39 (dd, *J =* 13.7, 7.4 Hz, 1H), 3.25 (dd, *J =* 13.7, 6.4 Hz, 1H), 1.58 (s, 3H), and 1.38 (s, 3H). HRMS (ESI/TOF-Q) m/z: [M + H]^+^ calcd. for C_21_H_23_BrN_5_O_5_S, 536.0598; found, 536.0604.

Methyl 4-((2′,3′-*O*-isopropylideneadenosyl)thio)-[1,1′]-biphenyl]-2-carboxylate (**9b**): Synthesized following general procedure P2 from **8b** (purity 80%, 0.10 g, 0.15 mmol) and phenylboronic acid (0.02 g, 0.16 mmol), yielded 47 mg, 59% as a white solid. See the following: ^1^H NMR (400 MHz, CDCl_3_) δ 8.34 (s, 1H), 7.90 (s, 1H), 7.78 (d, *J =* 2.0 Hz, 1H), 7.46 (dd, *J =* 8.1, 2.0 Hz, 1H), 7.42–7.32 (m, 3H), 7.30–7.26 (m, 2H) 7.24 (d, *J =* 8.1 Hz, 1H), 6.07 (d, *J =* 2.1 Hz, 1H), 5.95 (s, 2H), 5.54 (dd, *J =* 6.3, 2.1 Hz, 1H), 5.13 (dd, *J =* 6.3, 3.1 Hz, 1H), 4.44 (ddd, *J =* 7.6, 6.2, 3.1 Hz, 1H), 3.63 (s, 3H), 3.35 (dd, *J =* 13.7, 7.6 Hz, 1H), 3.26 (dd, *J =* 13.7, 6.2 Hz, 1H), 1.60 (s, 3H), and 1.40 (s, 3H); ^13^C NMR (100 MHz, CDCl_3_) δ 168.6, 155.7, 152.8, 149.2, 140.8, 140.6, 140.4, 134.7, 132.5, 131.6, 131.3, 131.0, 128.4, 128.2, 127.5, 120.3, 114.6, 91.3, 86.3, 84.2, 84.0, 52.2, 36.4, 27.2, and 25.4. HRMS (ESI/TOF-Q) m/z: [M + H]^+^ calcd. for C_27_H_28_N_5_O_5_S, 534.1806; found, 534.1809.

Methyl 5-((2′,3′-*O-*isopropylideneadenosyl)thio)-[1,1′-biphenyl]-2-carboxylate (**9c**): Synthesized following general procedure P2 from **8c** (purity 90%, 0.10 g, 0.17 mmol) and phenylboronic acid (22.0 mg, 0.18 mmol), yielded 75 mg, 84% as a white solid. See the following: ^1^H NMR (400 MHz, CDCl_3_) δ 8.30 (s, 1H), 7.85 (s, 1H), 7.76–7.71 (m, 1H), 7.41–7.32 (m, 3H), 7.29–7.23 (m, 4H), 6.06 (d, *J =* 2.1 Hz, 1H), 5.86 (br s, 2H), 5.54 (dd, *J =* 6.3, 2.1 Hz, 1H), 5.14 (dd, *J =* 6.3, 3.1 Hz, 1H), 4.43 (ddd, *J =* 7.6, 6.1, 3.1 Hz, 1H), 3.61 (s, 3H), 3.40 (dd, *J =* 13.7, 7.6 Hz, 1H), 3.25 (dd, *J =* 13.7, 6.1 Hz, 1H), 1.57 (s, 3H), and 1.38 (s, 3H); ^13^C NMR (100 MHz, CDCl3) δ 168.4, 155.8, 153.2, 149.2, 143.6, 140.9, 140.3, 140.2, 130.7, 130.2, 128.3, 128.1, 128.1, 127.6, 126.5, 120.6, 114.6, 91.2, 86.1, 84.1, 84.0, 52.0, 35.1, 27.2, and 25.4. HRMS (ESI/TOF-Q) m/z: [M + H]^+^ calcd. for C_27_H_28_N_5_O_5_S, 534.1806; found, 534.1815.

2-Methoxy-5-((adenosyl)thio)benzoic acid (**10d**): Synthesized following general procedure P4 from **8a** (70 mg, 0.14 mmol), yielded 22 mg, 35%, as a white solid. HPLC purity 97%. See the following: 1H NMR (400 MHz, DMSO-*d*_6_) δ 12.75 (br s, 1H), 8.34 (s, 1H), 8.15 (s, 1H), 7.63 (d, *J =* 2.5 Hz, 1H), 7.53 (dd, *J =* 8.7, 2.5 Hz, 1H), 7.28 (s, 2H), 7.06 (d, *J =* 8.7 Hz, 1H), 5.88 (d, *J =* 6.1 Hz, 1H), 5.49 (d, *J =* 5.1 Hz, 1H), 5.35 (br s, 1H), 4.81 (q, *J =* 5.0 Hz, 1H), 4.20–4.11 (m, 1H), 3.94 (ddd, *J =* 7.2, 6.0, 3.3 Hz, 1H), 3.79 (s, 3H), 3.33 (dd, overlapped with water, 1H), and 3.22 (dd, *J =* 13.7, 7.2 Hz, 1H); ^13^C NMR (100 MHz, DMSO-*d*_6_) δ 167.2, 157.6, 156.6, 153.1, 149.9, 140.5, 135.7, 133.1, 125.9, 122.7, 119.7, 113.9, 87.9, 83.3, 73.1, 73.0, 56.4, and 37.7. HRMS (ESI/TOF-Q) m/z: [M + H]^+^ calcd. for C_18_H_20_N_5_O_5_S, 434.1129; found, 434.1136.

4-((Adenosyl)thio)-[1,1′-biphenyl]-2-carboxylic acid (**10e**): Synthesized following general procedure P4 from **9b** (45 mg, 0.084 mmol), yielded 28 mg, 69%, as a white solid. HPLC purity 97%. See the following: ^1^H NMR (400 MHz, DMSO-*d*_6_) δ 12.73 (br s, 1H), 8.36 (s, 1H), 8.16 (s, 1H), 7.62 (d, *J =* 2.1 Hz, 1H), 7.52 (dd, *J =* 8.1, 2.1 Hz, 1H), 7.44–7.36 (m, 2H), 7.36–7.27 (m, 6H), 5.91 (d, *J =* 5.9 Hz, 1H), 5.54 (d, *J =* 4.5 Hz, 1H), 5.43 (br s, 1H), 4.84 (q, *J =* 4.8 Hz, 1H), 4.23 (t, *J =* 4.0 Hz, 1H), 4.04 (ddd, *J =* 7.1, 5.9, 3.4 Hz, 1H), 3.50 (dd, *J =* 13.8, 5.9 Hz, 1H), and 3.41 (dd, *J =* 13.8, 7.1 Hz, 1H); ^13^C NMR (100 MHz, DMSO-*d*_6_) δ 169.2, 156.1, 152.7, 149.5, 140.2, 140.0, 138.2, 135.3, 133.3, 131.1, 130.1, 128.3, 128.2, 128.1, 127.2, 119.3, 87.5, 82.8, 72.8, 72.6, and 35.1. HRMS (ESI/TOF-Q) m/z: [M + H]^+^ calcd. for C_23_H_22_N_5_O_5_S, 480.1336; found, 480.1346.

5-((Adenosyl)thio)-[1,1′-biphenyl]-2-carboxylic acid (**10f**): Synthesized following general procedure P4 from **9c** (73 mg, 0.14 mmol), yielded 47 mg, 72%, as a white solid. HPLC purity 95%. See the following: ^1^H NMR (400 MHz, DMSO-*d*_6_) δ 12.65 (br s, 1H), 8.35 (s, 1H), 8.12 (s, 1H), 7.66 (d, *J =* 8.2 Hz, 1H), 7.39 (dd, *J =* 8.2, 2.0 Hz, 1H), 7.38–7.32 (m, 3H), 7.29 (br s, 2H), 7.29–7.25 (m, 2H), 7.24 (d, *J =* 2.0 Hz, 1H), 5.90 (d, *J =* 5.7 Hz, 1H), 5.54 (d, *J =* 6.1 Hz, 1H), 5.40 (d, *J =* 5.0 Hz, 1H), 4.81 (q, *J =* 5.6 Hz, 1H), 4.25–4.20 (m, 1H), 4.06 (ddd, *J =* 7.1, 5.7, 3.6 Hz, 1H), 3.53 (dd, *J =* 13.8, 5.7 Hz, 1H), and 3.43 (dd, *J =* 13.8, 7.1 Hz, 1H); ^13^C NMR (100 MHz, DMSO-*d*_6_) δ 168.9, 156.1, 152.7, 149.5, 142.2, 140.5, 140.3, 140.0, 130.1, 128.8, 128.4, 128.3, 128.0, 127.3, 125.5, 119.2, 87.6, 82.7, 72.7, 72.7, and 34.1. HRMS (ESI/TOF-Q) m/z: [M + H]^+^ calcd. for C_23_H_22_N_5_O_5_S, 480.1336; found, 480.1340.

### 3.2. Molecular Modelling

Compounds designed were docked in the crystal structure of SARS-CoV-2 nsp14 (PDB ID: 7R2V) and human RNA (guanine-*N*7-)MTase (PDB ID: 3BGV) using the Schrodinger software package [26]. Protein crystal structures were prepared using Maestro Protein Preparation Wizard [27] by adding missing side chains using Prime [28], adjusting side chain protonation states at pH 7, and minimizing heavy atoms with convergence up to 0.30 Å. Inhibitors were prepared for docking using standard protocol implemented in LigPrep [27] at pH 7.

Docking studies were initiated by docking model validation, where the known and co-crystallized MTase adenosyl group-containing compounds (SAM, SAH, and SFG) were docked alongside 100 property-matched decoys (generated using DUD-E [29]). Docking models that returned SAM analogues in the correct docked pose (RMSD < 2 Å) and amongst the top scoring compounds were selected for further studies. Additionally, SAM analog docking was performed by restraining the adenosyl group of the inhibitor to the reference ligand adenosyl group pose with tolerance up to 0.1 Å. Scaling of the van der Waals radii was set to 0.9 for protein and ligand heavy atoms. Molecular docking was performed using Glide [30] at standard precision (Glide SP), and docked poses were visualized using PyMOL [31]. Compounds were prioritized based on docking scores against Nsp14, and the docking score against the human MTase was not considered for compound selection because the difference in the compound docking scores between the different MTases was at the level of scoring uncertainty (~2 kcal/mol).

### 3.3. Homogeneous Time-Resolved Fluorescent Energy Transfer (HTRF) Assay

Nsp10, nsp14, and nsp16 protein expression and purification, SARS-CoV-2 nsp16/nsp10 methyltransferase substrate RNA production were performed as described before [22]. Human Glycine N-Methyltransferase was obtained from MyBioSource, cat. nr. MBS636160. MTase activity was determined with an EPIgeneous Methyltransferase Assay kit by assaying the conversion of SAM to SAH according to the manufacturer’s instructions as described before [22].

### 3.4. Cell Permeability Testing

#### 3.4.1. Compound Incubation in Cell Culture

Permeability testing was performed in human non-small cell lung cancer cell line A549 (ATCC). Each compound was added to A549 cell culture at a concentration 20 μM and incubated for 24 h. Cells were seeded in 6-well plates at densities 2 × 10^4^ and 4 × 10^4^ cells per well at concentrations 1 × 10^4^ and 2 × 10^4^ cells/mL media for testing of each compound, each in three replicates. DMEM medium (Sigma, D6046, Irvin, UK) supplemented with 1% penicillin (100 U/mL)–streptomycin (100 μg/mL) and 10% fetal bovine serum (Sigma, F7524, St. Louis, MO, USA) was used for cell cultivation and all incubations were performed in a humidified 5% CO_2_ atmosphere at 37 °C. After the incubation, the cell cultivation media and cell lysates were collected. After the removal of media, cells were washed with ice-cold PBS (Sigma, D1408) and lysed for 30 min in 500 mL per well with ice-cold RIPA buffer (Sigma, R0278). Samples were stored at −80 °C until analysis.

#### 3.4.2. LC/MS/MS Analysis

The quantitative determination of tested compounds in cell lysates and culture media was performed on a Waters MICROMASS QUATTRO microTM tandem mass spectrometer combined with Acquity UPLC system as described before [22].

## Figures and Tables

**Table 1:** Inhibitory activities of compounds **5a–l** against SARS-CoV-2 nsp14, nsp16/nsp10 and hGNMT.

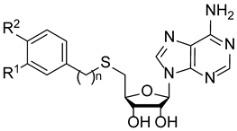				SARS-CoV-2		
Compound	n	R^1^	R^2^	nsp14, IC_50_, nM	nsp16/nsp10, IC_50_, nM	hGNMT, IC_50_, nM
**2a** ^1^	0	COOH	H	160 ± 20	40 ± 10	150 ± 30
**2b** ^1^	1	COOH	H	8.0 ± 0.6	4 ± 0.5	8.6 ± 0.4
**5a**	1	H	COOH	500 ± 30	1450 ± 70	400 ± 40
**5b**	1	–OCH_2_O–		730 ± 40	1980 ± 90	780 ± 40
**5c**	1	NHSO_2_Me	H	280 ± 20	480 ± 30	250 ± 20
**5d**	1	CH_2_COOH	H	680 ± 30	1840 ± 90	400 ± 30
**5e**	1	OH	H	1150 ± 60	8800 ± 400	600 ± 30
**5f**	1	CH_2_OH	H	740 ± 40	nd ^2^	1200 ± 100
**5g**	1	CN	H	351 ± 28	nd ^2^	724 ± 58
**5h**	1	1-tetrazole	H	340 ± 12	nd ^2^	220 ± 9
**5i**	1	SO_2_Me	H	270 ± 20	nd ^2^	250 ± 20
**5j**	1	SO_2_NHMe	H	400 ± 19	nd ^2^	350 ± 17
**5k**	1	COOMe	H	1350 ± 70	nd ^2^	870 ± 50
**5l**	1	CONHMe	H	2790 ± 140	nd ^2^	2780 ± 130

^1^ Ref. [22], ^2^ Not determined.

**Table 2:** Cell permeability of selected compounds.

Compound	Cell Permeability, % ^1^	
	1 × 10^4^ Cells/mL	2 × 10^4^ Cells/mL
**2a**	3.9%	9.3%
**2b**	<LOQ ^2^	<LOQ ^2^
**5c**	2.3%	2.3%
**5k**	6.6%	4.6%
**5p**	<LOQ ^2^	2.8%
**5r**	1.8%	2.2%
**10e**	3.7%	3.8%

^1^ The cell permeability is expressed in the percentage of compound concentration in cell lysates compared with the compound concentration in culture media after 24 h incubation. ^2^ Limit of quantification (0.2 μM or 1%).

**Table 3:** Inhibitory activities of compounds **5m–p,r,t,v**, and **10a–f** against SARS-CoV-2 nsp14, nsp16/nsp10, and hGNMT.

	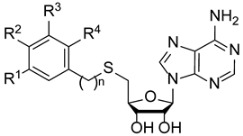					SARS-CoV-2		
Compound	n	R^1^	R^2^	R^3^	R^4^	nsp14, IC_50_, nM	nsp16/nsp10, IC_50_, nM	hGNMT, IC_50_, nM
**2a** ^1^	0	COOH	H	H	H	160 ± 20	40 ± 10	150 ± 30
**2b** ^1^	1	COOH	H	H	H	8.0 ± 0.6	4 ± 0.5	8.6 ± 0.4
**10a**	0	COOH	Cl	H	H	20 ± 2	30 ± 4	13 ± 2
**10b**	0	COOH	H	H	Cl	1300 ± 60	2300 ± 120	330 ± 20
**10c**	0	COOH	Br	H	H	24 ± 2	nd ^2^	8.2 ± 0.4
**10d**	0	COOH	OMe	H	H	17 ± 0.9	nd ^2^	13 ± 0.6
**10e**	0	COOH	Ph	H	H	25 ± 2	nd ^2^	24 ± 1
**10f**	0	Ph	COOH	H	H	420 ± 19	nd ^2^	210 ± 10
**5m**	1	COOH	Cl	H	H	7.7 ± 0.4	nd ^2^	6.6 ± 0.3
**5n**	1	COOH	F	H	H	16 ± 2	nd ^2^	6.1 ± 0.3
**5o**	1	COOH	H	Cl	H	24 ± 1.2	nd ^2^	15 ± 0.7
**5p**	1	COOH	H	Ph	H	0.58 ± 0.03	nd ^2^	0.97 ± 0.05
**5r**	1	COOH	Ph	H	H	36 ± 1.8	nd ^2^	10 ± 0.5
**5t**	1	COOH	H	*p*-OH-Ph	H	4.2 ± 0.2	nd ^2^	4.5 ± 0.2
**5v**	1	COOH	H	C≡CCH_2_OH	H	12 ± 0.6	nd ^2^	9.8 ± 0.5

^1^ Ref. [22], ^2^ Not determined.

## Data Availability

Not applicable.

## Supplementary Materials

The following supporting information can be downloaded at: https://www.mdpi.com/article/10.3390/molecules28020768/s1, synthesis of starting materials; copies of NMR spectra of final compounds **5a–p,r,t,v**, and **10a–f**.

## Sample Availability

Samples of the compounds **5a–p,r,t,v** and **10a–f** are available from the authors based on the material transfer agreement.
