# Supplementary material for: 3-(Adenosylthio)benzoic Acid Derivatives as SARS-CoV-2 Nsp14 Methyltransferase Inhibitors
Source: Molecules. 2023 Jan 12;28(2):768. doi: 10.3390/molecules28020768 (PMC9862586; doi:10.3390/molecules28020768)
Supplement: Supplementary file 1 [file molecules-28-00768-s001.zip › molecules-2131566-supplementary.pdf]

Supporting information

3-(Adenosylthio)benzoic Acid Derivatives as SARS-CoV-2 Nsp14 Methyltransferase Inhibitors

Olga Bobileva [1,*], Raitis Bobrovs [1], Evelina Elva Sirma [1], Iveta Kanepe [1], Anna L. Bula [1], Liene Patetko [2], Anna Ramata-Stunda [2], Solveiga Grinberga [1], Aigars Jirgensons [1] and Kristaps Jaudzems [1]

[1] Latvian Institute of Organic Synthesis, LV-1006 Riga, Latvia; raitis.bobrovs@osi.lv (R.B.); evelina.elva.sirma@osi.lv (E.E.S.); iveta@farm.osi.lv (I.K.); anna.lina.bula@osi.lv (A.L.B.); solveiga@osi.lv (S.G.); aigars@osi.lv (A.J.); kristaps.jaudzems@osi.lv (K.J.)

[2] Faculty of Biology, University of Latvia, LV-1004 Riga, Latvia; liene.patetko@gmail.com (L.P.); anna.ramata-stunda@lu.lv (A.R.-S.)

* Correspondence: olga_bobileva@osi.lv

Table of Contents

1 Synthesis of starting materials 2

1.1 General 2

1.2 Synthesis of benzyl bromides 2

1.3 Synthesis of thiophenols 7

2 ^{1}H and ^{13}C NMR copies of final products 10

1 Synthesis of starting materials

1.1 General

Reagents and solvents were obtained from commercial sources and used without purification. Dry THF, DCM, and toluene were prepared on MB-SPS MBraun solvent purifier system. Reaction conditions and yields were not optimized. Normal phase chromatography was performed on Davisil 60 Å 35-70 μm silica on a *Biotage Isolera One* purification system. Reactions were monitored by thin-layer chromatography using Merck F_{254} Alumina Silica Plates using UV visualization or staining. NMR spectra were recorded on 300 or 400 MHz Bruker spectrometers. Chemical shifts are reported in parts per million and referenced to the residual solvent signal. UPLC-MS analysis (referred as LCMS) was performed on Waters Acquity system: Acquity UPLC BEH-C18 (2.1 mm × 50 mm, 1.7 μm), gradient 0.01% HCOOH in water/CH_3CN 90%/10% to 10%/90% equipped with PDA (photodiode matrix) detector. GCMS analysis was obtained on *Agilent* 5975C gas chromatograph using *Agilent Technologies* (30 m × 0.250 mm) column. HRMS (ESI+) was obtained on a Waters Synapt G2-S*i* Mass Spectrometer.

1.2 Synthesis of benzyl bromides

*5-(Bromomethyl)benzo[d][1,3]dioxole (**S 4b**)*

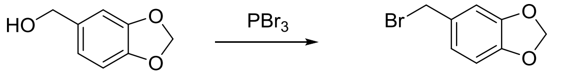

S 4b

To a solution of benzo[d][1,3]dioxol-5-ylmethanol (0.30 g, 1.97 mmol) in DCM (20 mL) was added PBr_3 (0.90 mL, 9.45 mmol) solution in DCM (9 mL) cooling the reaction mixture in the ice bath. The reaction mixture was stirred at room temperature for 2 h. The reaction mixture was quenched by the addition of water (50 mL), extracted with DCM (50 mL), the organic layer was washed with brine, dried over Na_2SO_4, filtered, and evaporated. The residue was chromatographed on silica gel, eluent EtOAc in petroleum ether 1:10 to obtain the title compound (0.28 g, 54%) as a white solid. ^{1}H NMR (300 MHz, $CDCl_3$) δ 6.90 – 6.83 (m, 2H), 6.77 – 6.71 (m, 1H), 5.97 (s, 2H), 4.46 (s, 2H). GCMS m/z: [M] calcd for $C_8H_7BrO_2$ 214.0; found 214.0.

*N-(3-(hydroxymethyl)phenyl)methanesulfonamide (**S 4c**)*

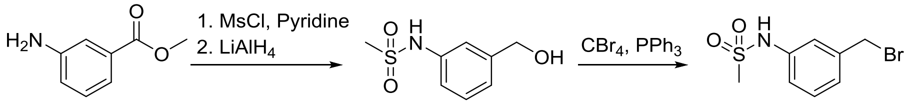

S 4c

To an ice-cold solution of 3-aminobenzoic acid methyl ester (0.50 g, 3.31 mmol) in DCM (10 mL) pyridine (0.50 mL, 6.19 mmol) was added, followed by methanesulfonyl chloride (0.30 mL, 3.87 mmol) cooling the reaction mixture in the ice bath. The reaction was stirred at room temperature for 2 h. Water (10 mL) and DCM (15 mL) were added, the aqueous layer was extracted with DCM (15 mL), and combined organic layers were washed with 1N HCl (10 mL). The organic layer was dried over Na_2SO_4, filtered, and concentrated under reduced pressure. The residue was dissolved in DCM and filtered through a short pad of silica, eluting with EtOAc in petroleum ether 25%, then 33%, the filtrate was concentrated to afford methyl 3-methanesulfonylaminobenzoate (0.67 g, 88%) as a white solid. ^{1}H NMR (300 MHz, $CDCl_3$) δ 7.92 – 7.84 (m, 2H), 7.54 (ddd, *J* = 8.1, 2.4, 1.3 Hz, 1H), 7.45 (t, *J* = 7.8 Hz, 1H), 6.96 (s, 1H), 3.94 (s, 3H), 3.04 (s, 3H). To a suspension of $LiAlH_4$ (0.09 g, 2.37 mmol) in dry THF (10 mL) under argon was added a solution of methyl 3-methanesulfonylaminobenzoate (0.34 g, 1.48 mmol) in dry THF (5 mL) dropwise over a period of 15 min, cooling reaction mixture in the ice bath. The mixture was stirred in the ice bath for 30 min, then at room temperature for 18 h. Additional $LiAlH_4$ (1 M solution in THF, 2.00 mL, 2.00 mmol) was added dropwise and the mixture was stirred for 1 h at room temperature. The reaction mixture was quenched with water (10 mL) and EtOAc (20 mL). Water layer was separated, acidified to pH 3 by the addition of 1 N HCl, then extracted with EtOAc (2 x 20 mL), combined organic layers were washed with brine and evaporated to obtain *N*-(3-(hydroxymethyl)phenyl)methanesulfonamide (0.25 g, 84%) as a slightly pink oil. ^{1}H NMR (300 MHz, $CDCl_3$) δ 7.35 (t, *J* = 7.8 Hz, 1H), 7.25 – 7.12 (m, 3H), 6.57 (s, 1H), 4.71 (s, 2H), 3.02 (s, 3H), 1.61 (s, 1H). To a stirred solution of *N*-(3-(hydroxymethyl)phenyl)methanesulfonamide (0.22 g, 1.09 mmol) in DCM (10 mL) under argon cooling in the ice bath was added PPh_3 (0.34 g, 1.31 mmol), and then CBr_4 (0.44 g, 1.33 mmol) in small portions. The reaction mixture was stirred for 1 h, then evaporated under reduced pressure. The

residue was chromatographed on silica gel on Biotage, eluent EtOAc in petroleum ether, gradient 20-80% to obtain the title compound (0.25 g, 87%) as a white solid. ^{1}H NMR (300 MHz, DMSO-*d6*) δ 9.84 (br s, 1H), 7.42 – 7.21 (m, 2H), 7.22 – 7.07 (m, 2H), 4.67 (s, 2H), 3.00 (s, 3H). ^{13}C NMR (75 MHz, DMSO-*d6*) δ 139.2, 138.7, 129.6, 124.6, 120.1, 119.5, 39.3, 34.2. HRMS (ESI/TOF-Q) m/z [M - H]$^-$ calcd for $C_8H_9BrNO_2S$, 261.9543; found, 261.9542.

*Methyl 2-(3-(bromomethyl)phenyl)acetate (**S 4d**)*

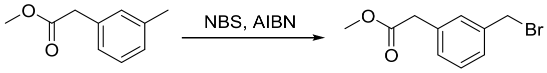

S 4d

To a solution of methyl 3-tolylacetate (0.50 g, 3.05 mmol) in CCl_4 (3 mL) was added NBS (0.54 g, 3.05 mmol) and AIBN (0.05 g, 0.31 mmol) and the reaction mixture was stirred at reflux for 3 h. The reaction mixture was evaporated. The residue was chromatographed on silica gel on Biotage, eluent EtOAc in petroleum ether 5% to obtain the title compound (0.44 g, 59%) as a white solid. ^{1}H NMR (300 MHz, $CDCl_3$) δ 7.37 – 7.28 (m, 3H), 7.25 – 7.19 (m, 1H), 4.48 (s, 2H), 3.70 (s, 3H), 3.63 (s, 2H). GCMS m/z [M] calcd for $C_{10}H_{11}BrO_2$ 242.0; found 242.0.

*(3-(Bromomethyl)phenoxy)(tert-butyl)dimethylsilane (**S 4e**)*

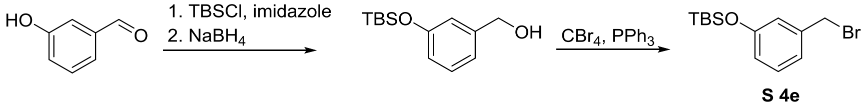

S 4e

To a solution of 3-hydroxybenzaldehyde (0.50 g, 4.09 mmol) in DCM (20 mL) was added imidazole (0.51 g, 7.48 mmol), the mixture was cooled in the ice bath, then and TBSCl (0.68 g, 4.50 mmol) was added and the reaction mixture was stirred at room temperature for 3 h. The reaction mixture was quenched with water, and extracted with DCM, the organic layer was separated, washed with brine, dried over Na_2SO_4, filtered, and evaporated. The residue was dissolved in dry EtOH (6 mL) and cooled in the ice bath, then $NaBH_4$ (0.24 g, 6.40 mmol) was added in portions over a period of 10 min, then the reaction mixture was stirred at room temperature for 1 h. The solvent was evaporated, the residue was partitioned between water and EtOAc, the organic layer was washed with brine, dried over Na_2SO_4, filtered, and evaporated to obtain (3-((*tert*-butyldimethylsilyl)oxy)phenyl)methanol (0.57 g, 58%). ^{1}H NMR (300 MHz, $CDCl_3$) δ 7.21 (t, *J* = 7.8 Hz, 1H), 6.98 – 6.90 (m, 1H), 6.86 (t, *J* = 1.9 Hz, 1H), 6.80 – 6.70 (m, 1H), 4.64 (s, 2H), 1.63 (s, 1H), 1.06 – 0.94 (m, 9H), 0.27 – 0.15 (m, 6H). To an ice-cold solution of (3-((*tert*-butyldimethylsilyl)oxy)phenyl) methanol (0.32 g, 1.35 mmol) in DCM (13 mL) was added PPh_3 (0.43 g, 1.62 mmol) followed by CBr_4 (0.54 g, 1.62 mmol) in small portions. The reaction mixture was stirred at room temperature for 1 h, then the solvent was evaporated. The residue was chromatographed on silica gel, eluent EtOAc in petroleum ether 5% to obtain the title compound (0.41 g, 82%) as a colorless oil. ^{1}H NMR (300 MHz, $CDCl_3$) δ 7.19 (t, *J* = 7.9 Hz, 1H), 6.97 (dt, *J* = 7.6, 1.4 Hz, 1H), 6.87 (t, *J* = 2.0 Hz, 1H), 6.77 (ddd, *J* = 8.1, 2.5, 1.0 Hz, 1H), 4.44 (s, 2H), 0.99 (s, 9H), 0.20 (s, 6H).). GCMS m/z [M] calcd for $C_{13}H_{21}BrOSi$ 300.1, found 300.1.

*(3-(Bromomethyl)phenyl)methanol (**S 4f**)*

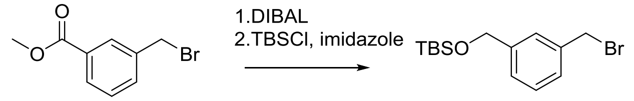

S 4f

To a solution of methyl 3-(bromomethyl)benzoate (0.30 g, 1.31 mmol) in dry DCM (6 mL) was added 1 DIBAL solution in toluene (1.2 M, 3.86 mL, 4.65 mmol) under argon at -78°C. The reaction mixture was stirred at -78°C for 1 h, then allowed to warm to room temperature and stirred for 3 h. The reaction mixture was quenched by the addition of potassium sodium tartrate (4.0 g, 14.2 mmol) in water (6 mL), stirred for 10 min, and then filtered through a pad of Celite. The filtrate was extracted with DCM, washed with brine, dried over anhydrous Na_2SO_4, and evaporated to give (3-(bromomethyl)phenyl)methanol (0.21 g, 80%) as a colorless solid. ^{1}H NMR (300 MHz, $CDCl_3$) δ 7.43 – 7.28 (m, 4H), 4.70 (d, *J* = 4.9 Hz, 2H), 4.50 (s, 2H), 1.73 (t, *J* = 4.9 Hz, 1H). To a solution of (3-(bromomethyl)phenyl)methanol (0.21 g, 1.06 mmol) and imidazole (0.13 g, 1.92 mmol) in DCM (6 mL) cooling in the ice bath was added a solution of TBSCl (0.18 g, 1.19 mmol) in DCM (2 mL), and the reaction mixture was stirred at 0 °C for 15 min, then 1 h at room temperature. The reaction mixture was diluted with EtOAc (30 mL), washed with brine, dried over Na_2SO_4, filtered, and evaporated. The residue was chromatographed on silica gel, eluent EtOAc in petroleum ether 10% to give the title

compound (0.21 g, 62%) as a colorless oil. ^{1}H NMR (300 MHz, $CDCl_3$) δ 7.35 – 7.27 (m, 4H), 4.73 (s, 2H), 4.50 (s, 2H), 0.95 (s, 9H), 0.12 (s, 6H). GCMS m/z [M] calcd for $C_{14}H_{23}BrOSi$ 314.1; found [M-57] 257.0.

*1-(Bromomethyl)-3-(methylsulfonyl)benzene (**S 4i**)*

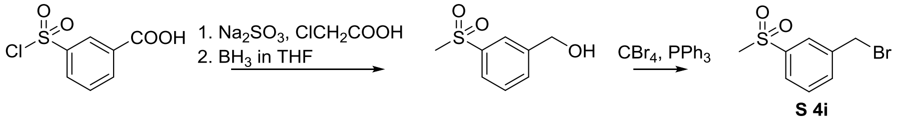

To a mixture of Na_2SO_3 (0.29 g, 2.27 mmol) and $NaHCO_3$ (0.42 g, 6.81 mmol) in water (2.5 mL) was added 3-(chlorosulfonyl)benzoic acid (0.50 g, 2.27 mmol) and the reaction mixture was stirred at 75 °C for 1 h, then chloroacetic acid (0.24 g, 3.41 mmol) was added to the reaction mixture, followed by NaOH (0.10 g, 3.41 mmol). The reaction mixture was stirred at 105 °C (bath temperature) for 24 h. The reaction mixture was acidified with 1 M HCl to pH 2, extracted with DCM (2 x 30 mL), dried over anhydrous Na_2SO_4, and evaporated. The filtrate was acidified with 1 N HCl and partially concentrated. The precipitate was filtered, washed with water and combined with the residue from DCM extract, then dried under reduced pressure to obtain 3-(methylsulfonyl)benzoic acid (0.19 g, 45%). ^{1}H NMR (300 MHz, DMSO-*d6*) 8.40 (s, 1H), 8.25 (d, *J*= 7.7 Hz, 1H), 8.16 (d, *J*= 7.7 Hz, 1H), 7.79 (t, *J*= 7.7 Hz, 1H), 3.27 (s, 3H). To a solution of 3-(methylsulfonyl)benzoic acid (0.19 g, 0.86 mmol) in dry THF (5 mL) under argon atmosphere was added 1 M BH_3 in THF (1.43 mL, 1.43 mmol) and the mixture was stirred at room temperature for 16 h. To the reaction mixture was added an additional 1 M BH_3 in THF (1.50 mL, 1.50 mmol) and the reaction mixture was stirred at room temperature for 2 h, then additional 1 M BH_3 in THF (1.00 mL, 1.00 mmol) was added, and the reaction mixture was stirred overnight. The reaction mixture was cooled in the ice bath, then MeOH (5 mL) was added dropwise, after 10 minutes, the volatiles were evaporated. The residue was chromatographed on silica gel on Biotage, eluent EtOAc:EtOH 3:1 in petroleum ether, gradient 20-80% to give (3-(methylsulfonyl)phenyl)methanol (0.12 g, 71%). ^{1}H NMR (300 MHz, $CDCl_3$) δ 7.95 (dt, *J* = 1.5 Hz, 1H), 7.85 (dt, *J* = 7.7, 1.5 Hz, 1H), 7.65 (dt, *J* = 7.9, 1.5 Hz, 1H), 7.56 (t, *J* = 7.8 Hz, 1H), 4.80 (s, 2H), 3.05 (s, 3H), 2.21 (s, 1H). Triphenylphosphine (0.21 g, 0.80 mmol) and CBr_4 (0.26 g, 0.80 mmol) were added to an ice-cold solution of (3-(methylsulfonyl)phenyl)methanol (0.12 g, 0.67 mmol) in dry DCM (6.5 mL). The reaction mixture was stirred at room temperature for 2 h. The solvent was removed under reduced pressure. The residue was purified by chromatography on silica gel on Biotage, eluent EtOAc:EtOH 3:1 in petroleum ether, gradient 20-80% to give 0.09 g (calculated yield 52%, contains PPh_3O as an impurity based on NMR) of the title compounds as a white solid. Used in the next step without additional purification. ^{1}H NMR (300 MHz, $CDCl_3$) 7.97 (t, *J* = 1.5 Hz, 1H), 7.88 (td, *J* = 7.8, 1.4 Hz, 1H), 7.70-7.64 (m, 1H), 7.57 (t, *J* = 7.8 Hz, 1H), 4.53 (s, 2H), 3.07 (s, 3H). LCMS (ESI$^+$) m/z $[M+H]^+$ calcd for $C_8H_{10}BrO_2S$ 249.0; found 248.9.

*3-Bromomethyl-N-methyl-benzenesulfonamide (**S 4j**)*

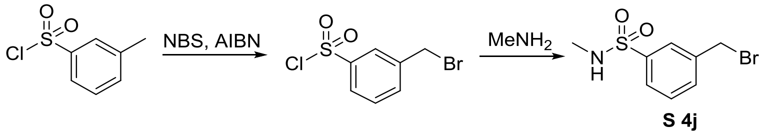

To solution of NBS (0.33 g, 1.24 mmol) in MeCN (3 mL) was added 3-methylbenzenesulfonyl chloride (0.24 g, 1.24 mmol) and AIBN (0.02 g, 0.12 mmol). The reaction mixture was stirred at reflux for 3 h, then additional AIBN (0.01 g, 0.06 mmol) was added and the reaction mixture was stirred for additional 20 minutes. The solvent was removed under reduced pressure, the residue was extracted with EtOAc (2 x 30 mL). The organic layer was washed with water, brine, dried over Na_2SO_4, filtered, and evaporated to obtain crude 3-(bromomethyl)benzenesulfonyl chloride. This residue was dissolved in Et_2O, and cooled in the ice bath, then TEA (0.16 mL, 1.18 mmol) was added, after 5 min followed by 2 M methylamine solution in THF (1.11 mL, 2.22 mmol). The reaction mixture was stirred at 0 °C for 3 h and concentrated under reduced pressure. The residue was dissolved in Et_2O (30 mL) and washed with sat. NH_4Cl. The organic phase was separated, dried over Na_2SO_4, and concentrated under reduced pressure. The residue was purified by chromatography on silica gel on Biotage, eluent EtOAc:EtOH 3:1 in petroleum ether, gradient 20-50% to give 0.14 g (52%) of the title compound as a white solid. ^{1}H NMR (300 MHz, $CDCl_3$) δ 7.89 (d, *J* = 1.5 Hz, 1H), 7.80 (dt, *J* = 7.7, 1.5 Hz, 1H), 7.62 (dt, *J* = 7.7, 1.5 Hz, 1H), 7.52 (t, *J* = 7.7 Hz, 1H), 7.44 – 7.37 (m, 1H), 4.52 (s, 2H), 2.70 (d, *J* = 5.4 Hz, 3H). No ionization was observed at standard LCMS conditions.

*Methyl 5-(bromomethyl)-2-chlorobenzoate (**S 4m**)*

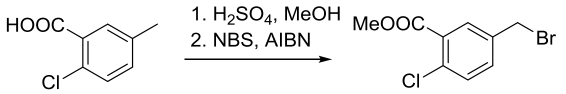

S 4m

To a solution of 2-chloro-5-methylbenzoic acid (0.66 g, 3.87 mmol) in MeOH (7 mL) was added conc. sulfuric acid (0.15 mL, 2.81 mmol), and the reaction mixture was stirred for 16 h at 50°C. The reaction mixture was concentrated, diluted with EtOAc (30 mL), and washed with sat. $NaHCO_3$, brine, dried over Na2SO4 and concentrated under reduced pressure to obtain methyl ester derivative (0.65 g, 91%) as a colorless oil. ^{1}H NMR (300 MHz, $CDCl_3$) δ 7.65 – 7.61 (m, 1H), 7.33 (d, *J* = 8.2 Hz, 1H), 7.24 – 7.19 (m, 1H), 3.93 (s, 3H), 2.35 (s, 3H). To a solution of methyl 2-chloro-5-methybenzoate (0.30 g, 1.63 mmol) in MeCN (8 mL) and DCM (0.5 mL) was added NBS (0.30 g, 1.69 mmol) and AIBN (18 mg, 0.11 mmol) in the dark, and the reaction mixture was stirred at reflux for 2 h. The solvent was evaporated and the reaction mixture was purified by chromatography on Biotage, eluent EtOAc in petroleum ether, gradient 1-6% to obtain the title compound (0.38 g, 89%) as a colorless oil. ^{1}H NMR (300 MHz, $CDCl_3$) δ 7.88 – 7.84 (m, 1H), 7.46 – 7.43 (m, 2H), 4.46 (s, 2H), 3.94 (s, 3H). GCMS m/z [M] calcd for $C_9H_8ClO_2$ 263.5; found 263.0.

*Methyl 5-(bromomethyl)-2-fluorobenzoate (**S 4n**)*

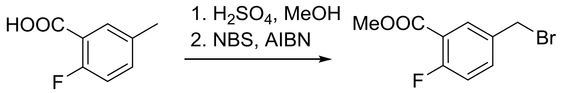

S 4n

To a solution of 2-fluoro-5-methylbenzoic acid (0.30 g, 1.95 mmol) in MeOH (5 mL) was added conc. sulfuric acid (0.10 mL, 1.95 mmol), and the reaction mixture was stirred for 16 h at 50 °C. The reaction mixture was concentrated, diluted with EtOAc (30 mL), and washed with sat. $NaHCO_3$, brine, dried over Na_2SO_4 and concentrated under reduced pressure to obtain methyl 2-fluoro-5-methylbenzoate (0.31 g, 93%) as a colorless oil. ^{1}H NMR (300 MHz, $CDCl_3$) δ 7.75 – 7.70 (m, 1H), 7.34 – 7.27 (m, 1H), 7.02 (dd, *J* = 10.6, 8.4 Hz, 1H), 3.92 (s, 3H), 2.35 (s, 3H). To a solution of methyl 2-fluoro-5-methybenzoate (0.30 g, 1.75 mmol) in MeCN (8 mL) was added NBS (0.33 g, 1.85 mmol) and AIBN (12 mg, 0.07 mmol) in the dark, and the reaction mixture was stirred at reflux for 6 h. The solvent was evaporated and the reaction mixture was purified by chromatography on Biotage, eluent EtOAc in petroleum ether, gradient 1-10% to obtain the title compound (0.29 g, 68%) as a colorless oil. ^{1}H NMR (300 MHz, $CDCl_3$) δ 7.97 (dd, *J* = 6.7, 2.5 Hz, 1H), 7.56 (ddd, *J* = 8.5, 4.5, 2.5 Hz, 1H), 7.13 (dd, *J* = 10.3, 8.5 Hz, 1H), 4.48 (s, 2H), 3.94 (s, 3H). GCMS m/z [M] calcd for $C_9H_8BrFO_2$ 246.0, found 246.0.

*Methyl 3-(bromomethyl)-5-chlorobenzoate (**S 4o**)*

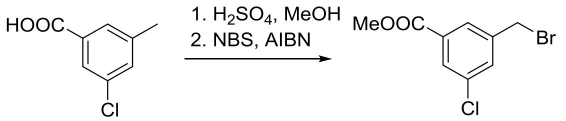

S 4o

To a solution of 3-chloro-5-methylbenzoic acid (0.24 g, 1.41 mmol) in MeOH (3 mL) was added conc. sulfuric acid (0.10 mL, 1.95 mmol), and the reaction mixture was stirred for 3 h at reflux. The reaction mixture was concentrated, diluted with EtOAc (20 mL), and washed with sat. $NaHCO_3$, brine, dried over Na_2SO_4 and concentrated under reduced pressure to obtain methyl 3-chloro-5-methylbenzoate (0.24 g, 93%) as a colorless oil. ^{1}H NMR (300 MHz, $CDCl_3$) δ 7.83 – 7.78 (m, 1H), 7.76 – 7.71 (m, 1H), 7.38 – 7.33 (m, 1H), 3.91 (s, 3H), 2.38 (s, 3H). To a solution of methyl 3-chloro-5-methybenzoate (0.24 g, 1.32 mmol) in MeCN (8 mL) was added NBS (0.24 g, 1.35 mmol) and AIBN (10 mg, 0.06 mmol) in the dark, and the reaction mixture was stirred at reflux for 46 h, additional AIBN (25 mg, 0.15 mmol) was added in 5 portions during this time. The solvent was evaporated and the reaction mixture was purified by chromatography on Biotage, eluent EtOAc in petroleum ether, gradient 1-10% to obtain the product (0.22 g, calculated yield 38%, purity 60%, contains methyl 3-chloro-5-methybenzoate as an impurity based on NMR) as a colorless oil. Used in the next step without additional purification.^{1}H NMR (300 MHz, $CDCl_3$) δ 7.97 (dd, *J* = 6.7, 2.5 Hz, 1H), 7.56 (ddd, *J* = 8.5, 4.5, 2.5 Hz, 1H), 7.13 (dd, *J* = 10.3, 8.5 Hz, 1H), 4.48 (s, 2H), 3.94 (s, 3H). GCMS m/z [M] calcd for $C_9H_8BrClO_2$ 261.9; found 261.9.

*Methyl 5-(bromomethyl)-[1,1′-biphenyl]-3-carboxylate (**S 4p**)*

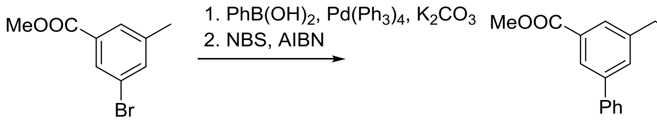

S 4p

To a mixture of methyl 3-bromo-5-methylbenzoate (0.23 g, 1.00 mmol), phenylboronic acid (0.13 g, 0.10 mmol), $Pd(PPh_3)_4$ (35 mg, 0.03 mmol), and K_2CO_3 (0.42 g, 3.01 mmol) in a vial under argon was added degassed mixture of dioxane and water (2 mL, 4:1), the vial was sealed and the mixture was stirred for 14 h at 100°C. The reaction mixture was filtered through a pad of silica, the filtrate was evaporated. The residue was chromatographed on silica gel on Biotage, eluent EtOAc in petroleum ether, gradient 1-15% to obtain methyl 5-methyl-[1,1'-biphenyl]-3-carboxylate (0.16 g, 72%) as a brown oil. ^{1}H NMR (300 MHz, $CDCl_3$) δ 8.08 (t, *J* = 1.6 Hz, 1H), 7.86 – 7.83 (m, 1H), 7.65 – 7.57 (m, 3H), 7.49 – 7.41 (m, 2H), 7.41 – 7.32 (m, 1H), 3.91 (s, 3H), 2.47 (s, 3H). To a solution of methyl 5-methyl-[1,1'-biphenyl]-3-carboxylate (0.16 g, 0.71 mmol) in CCl_4 (4 mL) was added NBS (0.14 g, 0.78 mmol) and AIBN (8 mg, 0.05 mmol) in the dark, and the reaction mixture was stirred at reflux for 4 h. The solvent was evaporated and the reaction mixture was purified by chromatography on Biotage, eluent EtOAc in petroleum ether, gradient 3-15% to obtain the title compound (0.18 g, calculated yield 63%, purity 75%, contains dibromide derivative based on LCMS) as a colorless oil. Used in the next step without additional purification. ^{1}H NMR (300 MHz, $CDCl_3$) δ 8.21 (t, *J* = 1.7 Hz, 1H), 8.05 (t, *J* = 1.7 Hz, 1H), 7.81 (t, *J* = 1.7 Hz, 1H), 7.67 – 7.59 (m, 2H), 7.52 – 7.37 (m, 3H), 4.58 (s, 2H), 3.96 (s, 3H). LCMS (ESI$^+$) m/z $[M+H]^+$ calcd for $C_{15}H_{14}BrO_2$ 305.0; found 305.0.

*Methyl 5-(bromomethyl)-2-bromobenzoate (**S 4q**)*

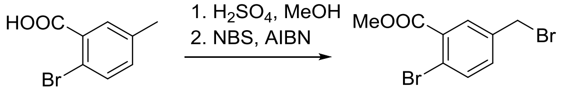

S 4q

To a solution of 2-bromo-5-methybenzoic acid (1.00 g, 4.88 mmol) in MeOH (10 mL) was added conc. sulfuric acid (0.30 mL, 4.13 mmol), and the reaction mixture was stirred for 3 h at reflux. The reaction mixture was concentrated, diluted with EtOAc (50 mL), and washed with sat. $NaHCO_3$, brine, dried over Na_2SO_4 and concentrated under reduced pressure to obtain methyl ester derivative (1.03 g, 92%) as a colorless oil. ^{1}H NMR (300 MHz, $CDCl_3$) δ 7.60 (d, *J* = 2.2 Hz, 1H), 7.53 (d, *J* = 8.2 Hz, 1H), 7.14 (ddq, *J* = 8.2, 2.2, 0.7 Hz, 1H), 3.93 (s, 3H), 2.33 (s, 3H). To a solution of methyl 2-bromo-5-methybenzoate (1.00 g, 4.36 mmol) in MeCN (8 mL) was added NBS (0.82 g, 4.58 mmol) and AIBN (28 mg, 0.17 mmol) in the dark, and the reaction mixture was stirred at reflux for 2.5 h. The solvent was evaporated and the residue was dissolved in Et_2O, washed with water (2 x 15 mL), brine, dried over Na_2SO_4, filtered, and concentered. The residue was purified by chromatography on silica gel on Biotage, eluent EtOAc in petroleum ether, gradient 3-15% to obtain the title compound (0.96 g, 71%) as a colorless solid. ^{1}H NMR (300 MHz, $CDCl_3$) δ 7.82 (d, *J* = 2.4 Hz, 1H), 7.64 (d, *J* = 8.3 Hz, 1H), 7.36 (dd, *J* = 8.3, 2.4 Hz, 1H), 4.44 (s, 2H), 3.95 (s, 3H). GCMS m/z [M] calcd for $C_9H_8Br_2O_2$ 307.9; found 307.9.

*Methyl 3-bromo-5-(bromomethyl)benzoate (**S 4s**)*

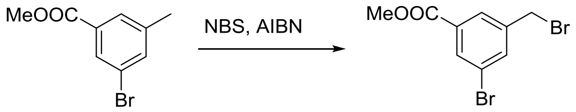

S 4s

To a solution of methyl 3-bromo-5-methybenzoate (0.30 g, 1.31 mmol) in MeCN (8 mL) was added NBS (0.23 g, 1.31 mmol) and AIBN (8.6 mg, 0.05 mmol) in the dark, and the reaction mixture was stirred at reflux for 3 h. The solvent was evaporated and the reaction mixture was purified by chromatography on Biotage, eluent EtOAc in petroleum ether, gradient 3-10% to obtain the title compound (0.32 g, calculated yield 63%, purity 80% based on NMR) as a colorless oil. Used in the next step without additional purification. ^{1}H NMR (300 MHz, $CDCl_3$) δ 8.10 (t, *J* = 1.7 Hz, 1H), 7.99 (t, *J* = 1.7 Hz, 1H), 7.73 (t, *J* = 1.7 Hz, 1H), 4.45 (s, 2H), 3.93 (s, 3H). GCMS m/z [M] calcd for $C_9H_8Br_2O_2$ 307.9; found 307.9.

*Methyl 3-(bromomethyl)-5-iodobenzoate (**S 4u**)*

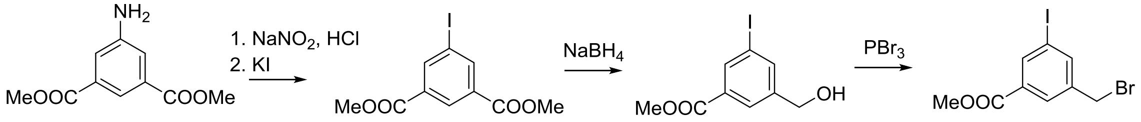

S 4u

A solution of $NaNO_2$ (1.73 g, 25.1 mmol) in water (5 mL) was added to a stirred cold suspension (-3 °C, reaction mixture temperature) of dimethyl 5-aminoisophthalate (5.00 g, 23.9 mmol) in 10% aqueous hydrochloric acid (85 mL). The mixture was stirred at 0 °C for 1 h, then a solution of KI (15.8 g, 95.0 mmol) in water (30 mL) was added dropwise, and the reaction mixture was allowed to warm to room temperature and stirred for 15 h. The resulting dark suspension was supplemented with DCM (100 mL) and EtOAc (100 mL) organic layer was separated, and the water layer was washed with EtOAc (100 mL). Combined organic extracts were washed with 10% $Na_2S_2O_5$ (2 x 50 mL), then water, brine, dried over Na2SO4, filtered, and concentrated. This residue was chromatographed on silica gel on Biotage, eluent EtOAc in petroleum ether, gradient 10-15% to obtain dimethyl 5-iodoisophthalate (3.74 g, calculated yield 42%, purity 85% based on NMR) as slightly yellow solid, Used in the next step without additional purification. ^{1}H NMR (400 MHz, $CDCl_3$) δ 8.63 (t, *J* = 1.5 Hz, 1H), 8.54 (d, *J* = 1.5 Hz, 2H), 3.95 (s, 6H). To the solution of dimethyl 5-iodoisophthalate (2.50 g, 7.81 mmol) and $NaBH_4$ (0.44 g, 11.7 mmol) in dry THF (25 mL) at 50 °C was added MeOH (2.5 mL) dropwise over a period of 30 min. The reaction mixture was stirred at 50 °C for 2 h, then cooled to room temperature. The reaction mixture was diluted with EtOAc (60 mL), washed with water (2 x 20 mL), brine, dried over Na_2SO_4, and concentrated under reduced pressure. The residue was chromatographed on silica gel on Biotage, eluent EtOAc in petroleum ether, gradient 15-30% to obtain methyl 3-(hydroxymethyl)-5-iodobenzoate (1.30 g, 57%) as a white solid. ^{1}H NMR (400 MHz, $CDCl_3$) δ 8.29 (t, *J* = 1.6 Hz, 1H), 7.98 (tt, *J* = 1.6, 0.7 Hz, 1H), 7.93 (tt, *J* = 1.6, 0.7 Hz, 1H), 4.72 (dq, *J* = 5.9, 0.7 Hz, 2H), 3.92 (s, 3H), 1.81 (t, *J* = 5.9 Hz, 1H). To a cooled solution of methyl 3-(hydroxymethyl)-5-iodobenzoate (1.36 g, 4.66 mmol) in DCM (20 mL) in the ice bath under argon was added PBr_3 (0.48 mL, 5.00 mmol) slowly dropwise, and the reaction mixture was stirred in the ice bath for 30 min. The reaction mixture was supplemented with DCM (20 mL) and sat. $NaHCO_3$ (15 mL), was stirred for 10 min, then the organic layer was separated, washed with water, brine, dried over Na_2SO_4, and evaporated. The residue was chromatographed on silica gel on Biotage, eluent EtOAc in petroleum ether, gradient 7-20% to obtain the title compound (0.65 g, 40%) as a white solid. ^{1}H NMR (400 MHz, $CDCl_3$) δ 8.30 (t, *J* = 1.6 Hz, 1H), 8.02 (t, *J* = 1.6 Hz, 1H), 7.92 (t, *J* = 1.6 Hz, 1H), 4.42 (s, 2H), 3.93 (s, 3H). GCMS m/z [M] calcd for $C_9H_8BrIO_2$ 353.9; found 353.9.

1.3 Synthesis of thiophenols

*2-Chloro-5-mercaptobenzoic acid (**S 10a**)*

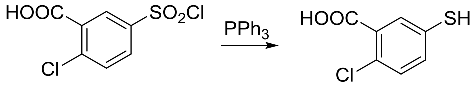

S 10a

To a solution of 2-chloro-5-(chlorosulfonyl)benzoic acid (0.51 g, 2.00 mmol) in dry toluene (3 mL) was added Ph_3P (1.57, 6.00 mmol). The reaction mixture was stirred for 50 min, then water (2 mL) was added and the mixture was stirred for an additional 10 min. The aqueous layer was separated and the organic layer was extracted with 10% NaOH (2 × 5 mL). The alkaline aqueous extract was washed with DCM (2 × 10 mL), cooled in the ice-bath and acidified with 3 N HCl to pH 2. The precipitate was filtered, washed with water and dried *in vacuo* over P_2O_5 to obtain the title compound (0.13 g, 34%) as a white solid. ^{1}H NMR (300 MHz, $CDCl_3$) δ 13.19 (br s, 1H), 7.92 (t, *J* = 1.3 Hz, 1H), 7.38 – 7.34 (m, 2H), 3.57 (s, 1H). LCMS (ESI$^-$) m/z [M - H]$^-$ calcd for $C_7H_4ClO_2S$ 187.0; found 187.0.

*4-Chloro-3-mercaptobenzoic acid (**S 10b**)*

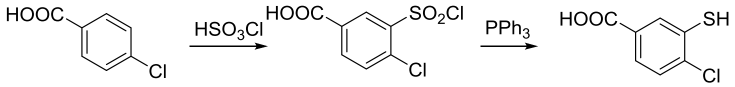

S 10b

4-Chlorobenzoic acid (0.71 g, 4.56 mmol) was added in portions to a chlorosulfonic acid (1.3 mL, 19.6 mmol) cooling in the ice bath. The reaction mixture was heated to 100 °C for 16 h. After cooling to room temperature, the reaction mixture was poured into ice and extracted with EtOAc (2 x 20 mL) The organic layers were combined, washed with

water and brine, dried over Na_2SO_4, filtered, and concentrated under reduced pressure to obtain 4-chloro-3-(chlorosulfonyl)benzoic acid (0.96 g, 82%) as an off-white solid. ^{1}H NMR (400 MHz, CDCl3) δ 8.86 (d, *J* = 2.0 Hz, 1H), 8.36 (dd, *J* = 8.3, 2.0 Hz, 1H), 7.79 (d, *J* = 8.3 Hz, 1H). To a solution of 4-chloro-3-(chlorosulfonyl)benzoic acid (0.50 g, 1.96 mmol) in dry toluene (3 mL) was added Ph_3P (1.54, 5.88 mmol). The reaction mixture was stirred for 50 min, then water (2 mL) was added and the mixture was stirred for an additional 10 min. The aqueous layer was separated and the organic layer was extracted with 10% NaOH (2 × 5 mL). The alkaline aqueous extract was washed with DCM (2 × 10 mL), cooled in the ice bath, and acidified with 3N HCl to pH 2. The precipitate was filtered, washed with water, and dried *in vacuo* over P_2O_5 to obtain the title compound (0.14 g, 37%) as a white solid. ^{1}H NMR (400 MHz, DMSO-d_6) δ 13.26 (br s, 1H), 8.20 – 8.12 (m, 1H), 7.68 – 7.61 (m, 1H), 7.59 – 7.49 (m, 1H), 6.22 (s, 1H). LCMS (ESI$^-$) m/z [M-H]$^-$ calcd for $C_7H_4ClO_2S$ 187.0, found 187.0.

*2-Bromo-5-mercaptobenzoic acid (**S 10c**) and methyl 2-bromo-5-mercaptobenzoate (**S 8b**)*

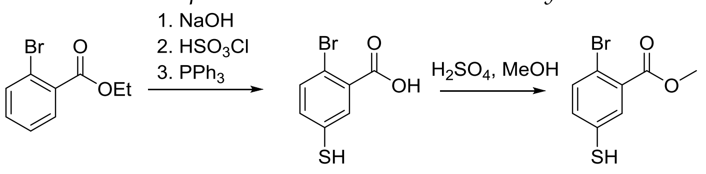

S 10c **S 8b**

To a solution of ethyl 2-bromobenzoate (1.6 g, 7.0 mmol) in THF (5 mL) and MeOH (5 mL) was added 2 N NaOH (10 mL, 20 mmol) and the reaction mixture was stirred for 2 h at 60 °C. The reaction mixture was cooled to room temperature and concentrated under reduced pressure. The residue was diluted with water (10 mL) and acidified with 3 N HCl to pH 3. The mixture was extracted with EtOAc (2 x 30 mL), combined organic layers were dried over Na_2SO_4, filtered, and concentrated. Chlorosulfonic acid (2.8 mL, 42 mmol) was added to the residue and the reaction mixture was heated at 140 °C for 3 h. After cooling, the solution was poured into ice and extracted with EtOAc (2 x 30 mL). The organic layers were combined, washed with brine, dried over Na_2SO_4, filtered, and concentrated to obtain 2-bromo-5-(chlorosulfonyl)benzoic acid (1.7 g, 81%) as a beige solid. ^{1}H NMR (300 MHz, $CDCl_3$) δ 11.56 (br s, 1H), 8.65 (dd, *J* = 1.9, 1.0 Hz, 1H), 8.06 – 7.99 (m, 2H). To a solution of 2-bromo-5-(chlorosulfonyl)benzoic acid (0.71 g, 2.37 mmol) in dry toluene (12 mL) was added Ph_3P (1.86 g, 7.11 mmol). The reaction mixture was stirred for 50 min at 60 °C, then 15% NaOH solution (12 mL) was added and the mixture was stirred for an additional 10 min. The alkaline aqueous extract was separated, washed with toluene (10 mL), then DCM (2 x 15 mL), cooled in the ice bath, and acidified with 3 N HCl to pH 1. The precipitate was extracted with EtOAc (2 x 30 mL), washed with brine, dried over Na_2SO_4, filtered, and concentrated to obtain 2-bromo-5-mercaptobenzoic acid (**S 10c**) (0.33 g, 60%) as a brown solid. ^{1}H NMR (300 MHz, $CDCl_3$) δ 10.32 (br s, 1H), 7.89 (d, *J* = 2.4 Hz, 1H), 7.56 (d, *J* = 8.3 Hz, 1H), 7.27 (dd, *J* = 8.3, 2.4 Hz, 1H), 3.55 (s, 1H). LCMS (ESI$^-$) m/z: [M-H]$^-$ calcd for $C_7H_4BrO_2S$: 230.9, found 230.8. To a solution of 2-bromo-5-mercaptobenzoic acid (0.53 g, 2.27 mmol) in MeOH (8 mL) was added sulfuric acid (0.10 mL, 1.88 mmol) and the reaction mixture was stirred at reflux for 8 h. The reaction mixture was evaporated, diluted with sat. NH_4Cl solution, and extracted with EtOAc (2 x 15 mL), organic layers were combined, and washed with sat. NH_4Cl solution, brine, dried over Na_2SO_4, filtered and evaporated to obtain the title compound (0.57 g, calculated yield 91%, purity 89% based on LCMS) as a brown oil. ^{1}H NMR (300 MHz, $CDCl_3$) δ 7.70 (d, *J* = 2.4 Hz, 1H), 7.51 (d, *J* = 8.4 Hz, 1H), 7.22 (dd, *J* = 8.4, 2.4 Hz, 1H), 3.93 (s, 3H), 3.52 (s, 1H). LCMS (ESI$^-$) m/z [M - H]$^-$ calcd for $C_8H_6BrO_2S$ 244.9; found 244.8.

*Methyl 5-mercapto-2-methoxybenzoate (**S 8a**)*

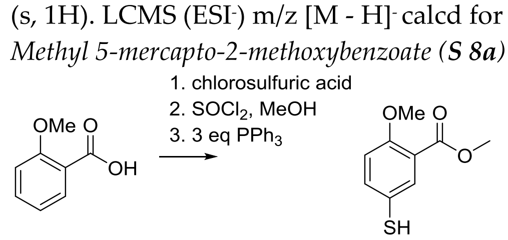

S 8a

2-Methoxybenzoic acid (0.40 g, 2.63 mmol) was added in portions to chlorosulfonic acid (1.50 mL, 22.5 mmol) cooling in the ice bath. The reaction mixture was stirred at room temperature for 16 h. The reaction mixture was poured into ice and extracted with EtOAc (2 x 25 mL) The organic layers were combined, washed with water and brine, dried over Na_2SO_4, filtered, and concentrated under reduced pressure to obtain 5-(chlorosulfonyl)-2-methoxybenzoic acid (0.57

g, 87%) as a white solid. ^{1}H NMR (300 MHz, $CDCl_3$) δ 10.55 (br s, 1H), 8.77 (d, *J* = 2.6 Hz, 1H), 8.22 (dd, *J* = 9.0, 2.6 Hz, 1H), 7.25 (d, *J* = 9.0 Hz, 1H), 4.17 (s, 3H). To a suspension of 5-(chlorosulfonyl)-2-methoxybenzoic acid (0.57 g, 2.27 mmol) in DCM (2 mL) was added thionyl chloride (2.00 mL, 27.6 mmol) cooling the reaction mixture in the ice bath, then the reaction mixture was heated at 60 °C for 3 h. The reaction mixture was allowed to cool to room temperature. The reaction mixture was concentrated, and the resulting clear oil was diluted with DCM and evaporated. The crude benzoyl chloride derivative was cooled in an ice-water bath and cold methanol (3 mL) was added. The reaction mixture was stirred for 10 min in the ice bath. The reaction mixture was quenched by the addition of cold water, extracted with EtOAc (2 x 20 mL), washed with brine, dried over Na_2SO_4, filtered, and evaporated to obtain methyl 5-(chlorosulfonyl)-2-methoxybenzoate (0.55 g, 91%) as a yellow oil. ^{1}H NMR (300 MHz, $CDCl_3$) δ 8.48 (d, *J* = 2.6 Hz, 1H), 8.14 (dd, *J* = 9.0, 2.6 Hz, 1H), 7.15 (d, *J* = 9.0 Hz, 1H), 4.04 (s, 3H), 3.94 (s, 3H). To a solution of methyl 5-(chlorosulfonyl)-2-methoxybenzoate (0.25 g, 0.94 mmol) in dry toluene (7 mL) was added Ph_3P (0.74 g, 2.83 mmol) in portions. The reaction was stirred for 50 min at room temperature. The reaction mixture was concentrated under reduced pressure. The residue was chromatographed on silica gel on Biotage, eluent EtOAc in petroleum ether 5-30% to give the title compound (52 mg, 28%) as a yellow oil. ^{1}H NMR (300 MHz, $CDCl_3$) δ 7.78 (d, *J* = 2.5 Hz, 1H), 7.43 (dd, *J* = 8.7, 2.5 Hz, 1H), 6.88 (d, *J* = 8.7 Hz, 1H), 3.89 (s, 3H), 3.88 (s, 3H), 3.40 (s, 1H). LCMS (ESI$^-$) m/z [M - H]$^-$ calcd for $C_9H_9O_3S$ 197.0; found 196.9.

*Methyl 2-bromo-4-mercaptobenzoate (**S 8c**)*

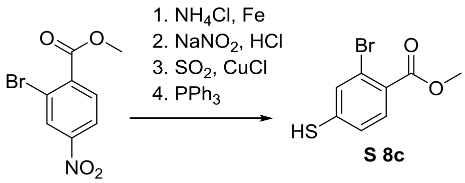

A solution of methyl 2-bromo-4-nitrobenzoate (1.00 g, 3.85 mmol), iron powder (1.29 g, 23.1 mmol), and ammonium chloride (0.25 g, 4.61 mmol) in aqueous 2-propanol (20%, 15 mL) was heated to reflux for 50 minutes, then the reaction mixture was cooled to room temperature. The precipitate was filtered, and washed with THF (10 mL), the filtrate was concentrated under reduced pressure. The residue was partitioned between diethyl ether (20 mL) and water (5 mL). The organic layer was washed with brine, dried over Na_2SO_4, filtered, and concentrated to give methyl 4-amino-2-bromobenzoate (0.52 g, 59%) as an orange solid. ^{1}H NMR (300 MHz, $CDCl_3$) δ 7.78 (d, *J* = 8.5 Hz, 1H), 6.95 (d, *J* = 2.4 Hz, 1H), 6.59 (dd, *J* = 8.5, 2.4 Hz, 1H), 4.06 (br s, 2H), 3.88 (s, 3H). SO_2 solution in water was prepared as follows: thionyl chloride (1.30 mL, 17.9 mmol) was added slowly to cold water (6 mL) and stirred for 18 h at room temperature. Hydrochloric acid (35-37% w/w, 4.00 mL) was added to methyl 4-amino-2-bromobenzoate (0.74 g, 3.22 mmol), cooling the flask in the ice bath. The mixture was sonicated for 3 min, then the mixture was cooled to -5 °C using a dry ice/acetone bath. A cold solution of sodium nitrite (0.30 g, 4.35 mmol) in water (1 mL) was added dropwise over 10 min. The resultant orange slurry was cooled to -5 °C and stirred for 10 min. To a previously prepared ice-cold SO_2 solution in water was added CuCl (5 mg, 0.05 mmol). The resulting mixture was added to the diazonium salt slurry over 10 min, cooling the reaction mixture at below -5 °C. The reaction mixture was stirred in the ice bath for 1 h, then warmed to room temperature and stirred for 1 h. The reaction mixture was extracted with EtOAc (3 x 20 mL), organic layers were combined and washed with brine, dried over Na_2SO_4, filtered, and evaporated to obtain methyl 2-bromo-4-(chlorosulfonyl)benzoate (0.70 g, 69%) as an orange oil. ^{1}H NMR (300 MHz, $CDCl_3$) δ 8.31 (d, *J* = 1.9 Hz, 1H), 8.03 (dd, *J* = 8.3, 1.9 Hz, 1H), 7.95 (d, *J* = 8.3 Hz, 1H), 4.00 (s, 3H). To a solution of methyl 2-bromo-4-(chlorosulfonyl)benzoate (0.30 g, 0.96 mmol) in dry toluene (5 mL) was added Ph_3P (0.75 g, 2.87 mmol) in portions. The reaction was stirred for 50 min at room temperature. The reaction mixture was concentrated under reduced pressure. The residue was chromatographed on silica gel on Biotage, eluent EtOAc in petroleum ether, gradient 5-30% to give methyl 2-bromo-4-mercaptobenzoate (0.17 g, 72%) as a yellow oil. ^{1}H NMR (300 MHz, $CDCl_3$) δ 7.71 (d, *J* = 8.2 Hz, 1H), 7.56 (d, *J* = 1.9 Hz, 1H), 7.21 (dd, *J* = 8.2, 1.9 Hz, 1H), 3.91 (s, 3H), 3.60 (s, 1H). LCMS (ESI$^-$) m/z [M-H]$^-$ calcd for $C_8H_6BrO_2S$ 244.9; found 244.9.

2 ¹H and ¹³C NMR copies of final products

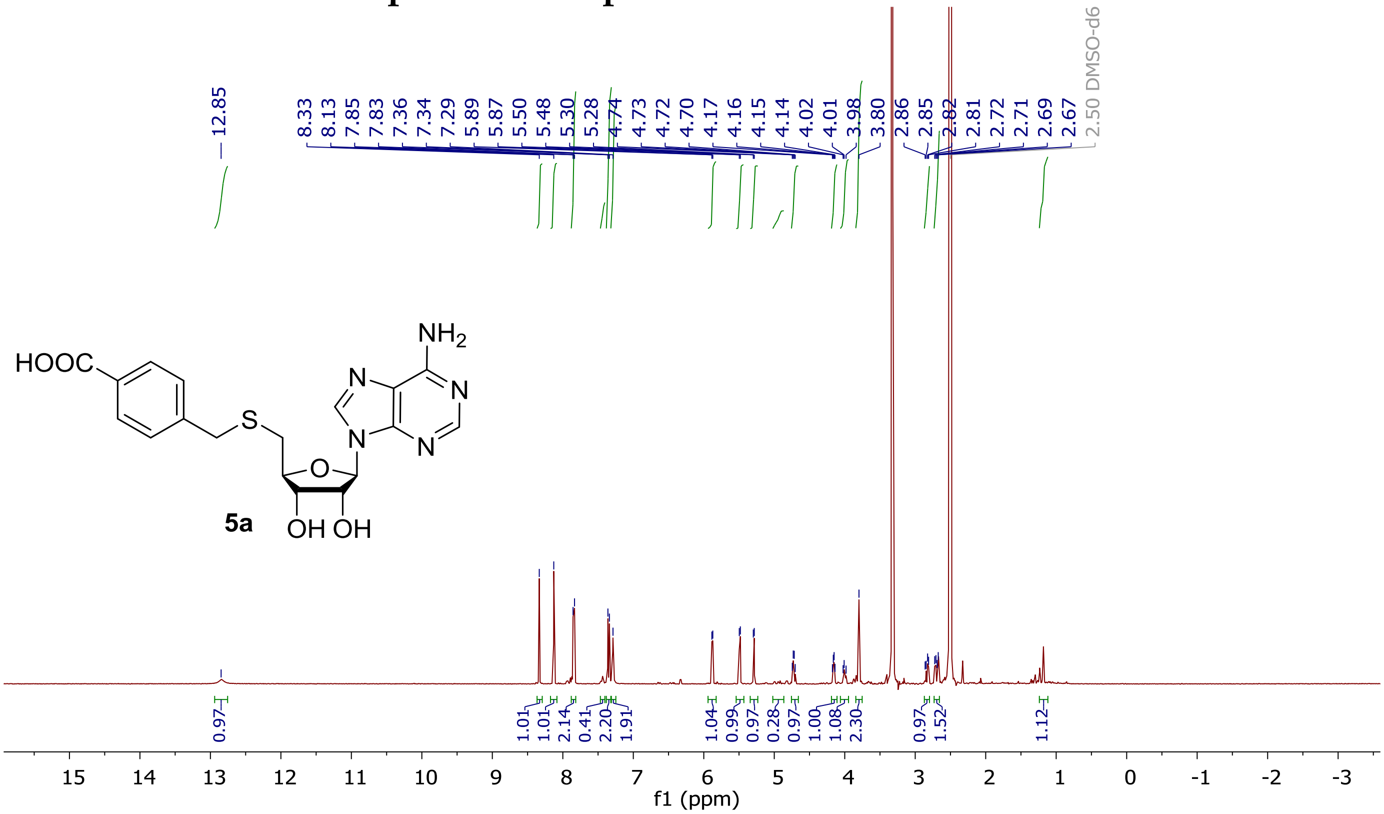

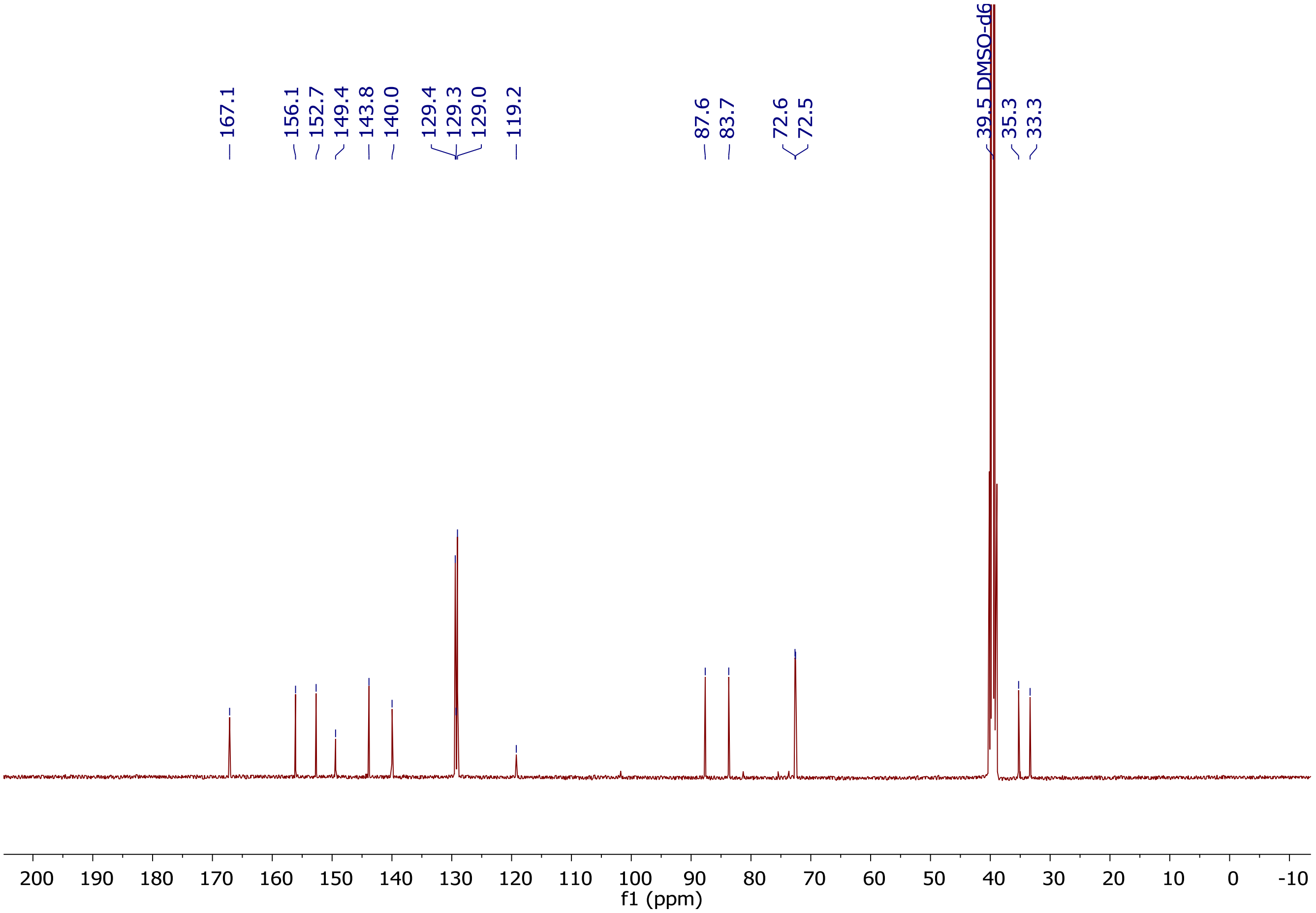

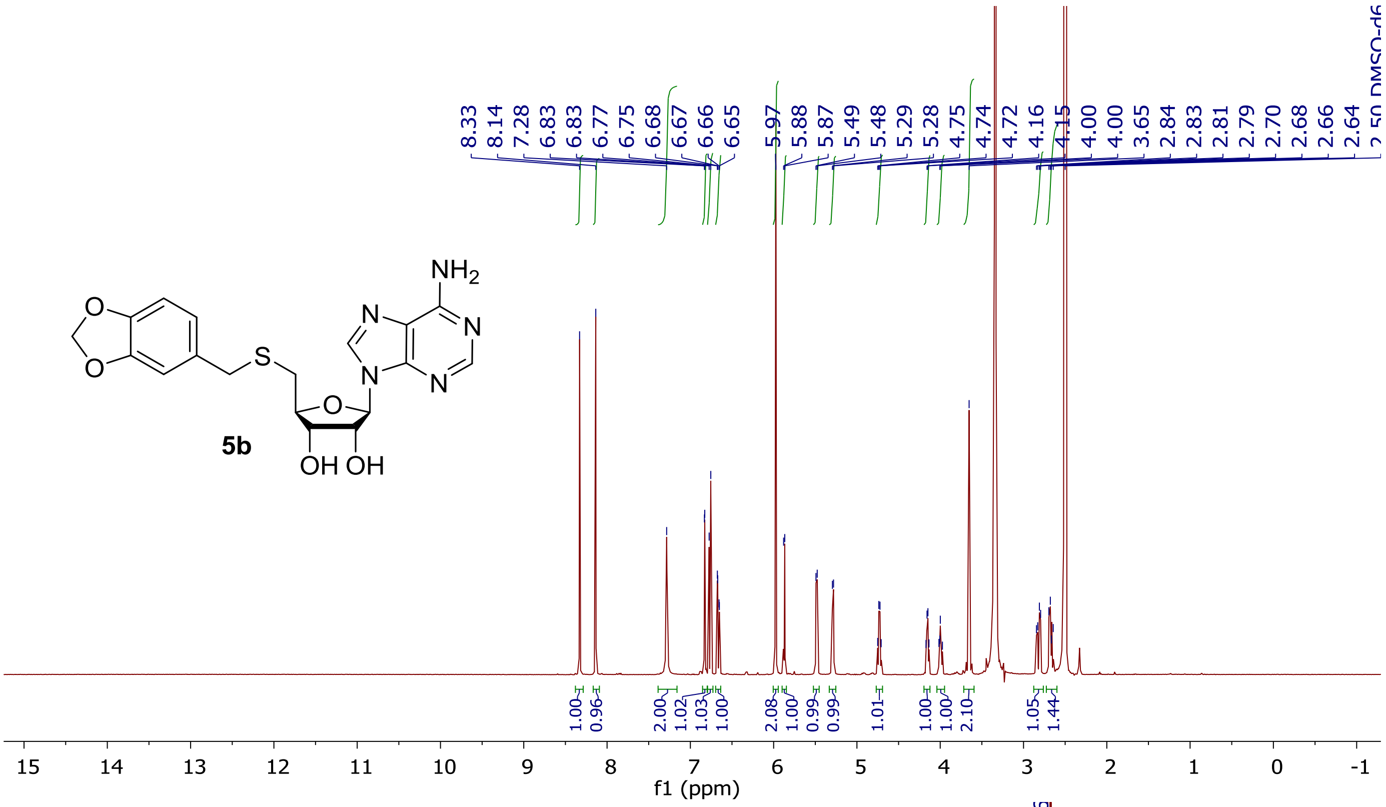
NH2
N
N
N
N
O
O
S
O
OH OH
8.33
8.14
7.28
6.83
6.83
6.77
6.75
6.68
6.67
6.66
6.65
5.97
5.88
5.87
5.49
5.48
5.29
5.28
4.75
4.74
4.72
4.16
4.15
4.00
4.00
3.65
2.84
2.83
2.81
2.79
2.70
2.68
2.66
2.64
1.00
0.96
2.00
1.02
1.03
1.00
2.08
1.00
0.99
0.99
1.01
1.00
1.00
2.10
1.05
1.44
f1 (ppm)

5b

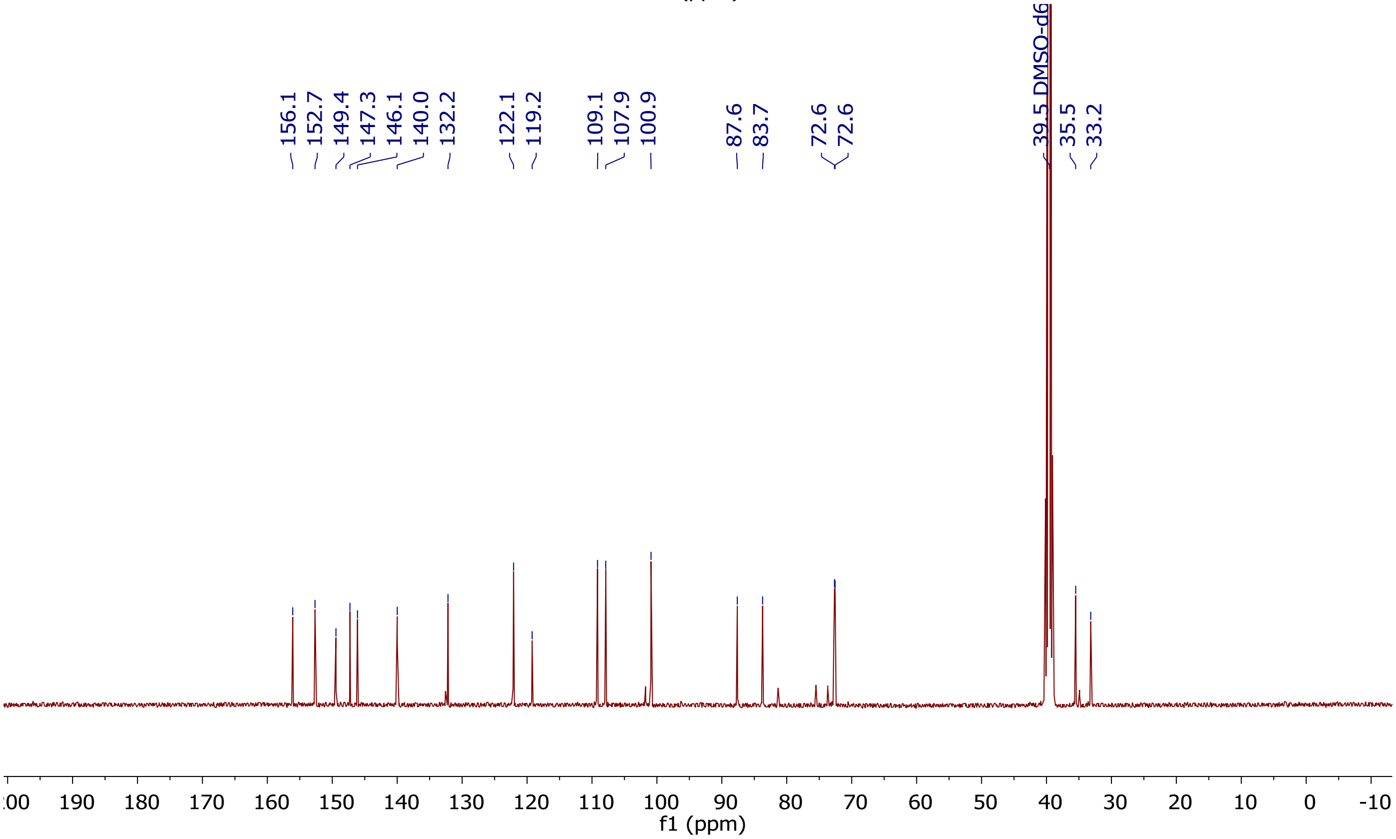
156.1
152.7
149.4
147.3
146.1
140.0
132.2
122.1
119.2
109.1
107.9
100.9
87.6
83.7
72.6
72.6
39.5 DMSO-d6
35.5
33.2
f1 (ppm)

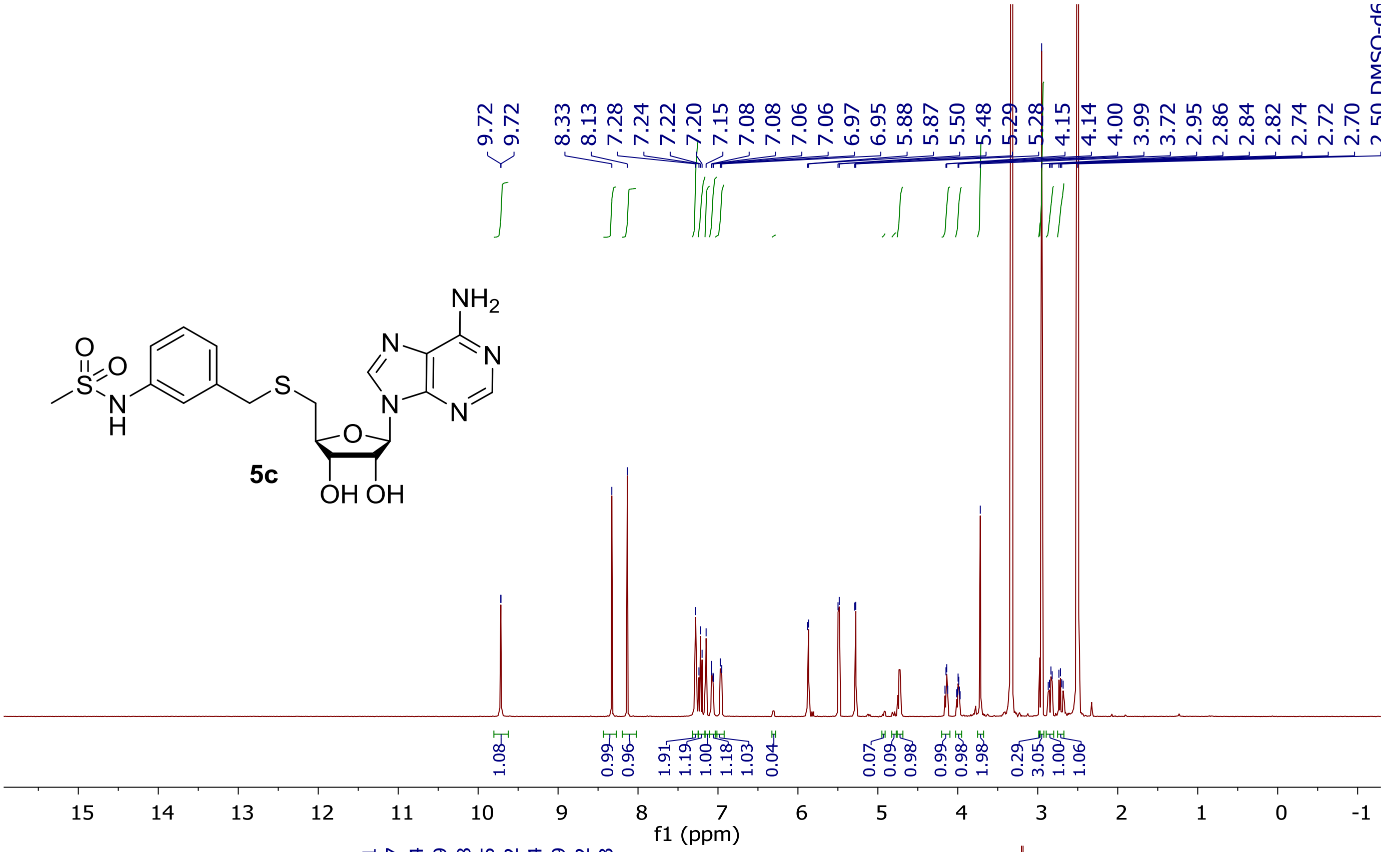
9.72
9.72
8.33
8.13
7.28
7.24
7.22
7.20
7.15
7.08
7.08
7.06
7.06
6.97
6.95
5.88
5.87
5.50
5.48
5.29
5.28
4.15
4.14
4.00
3.99
3.72
2.95
2.86
2.84
2.82
2.74
2.72
2.70
NH2
N
N
N
N
O
O
S
N
H
S
O
OH OH
5c
1.08
0.99
0.96
1.91
1.19
1.00
1.18
1.03
0.04
0.07
0.09
0.98
0.99
0.98
1.98
0.29
3.05
1.00
1.06
15 14 13 12 11 10 9 8 7 6 5 4 3 2 1 0 -1
f1 (ppm)

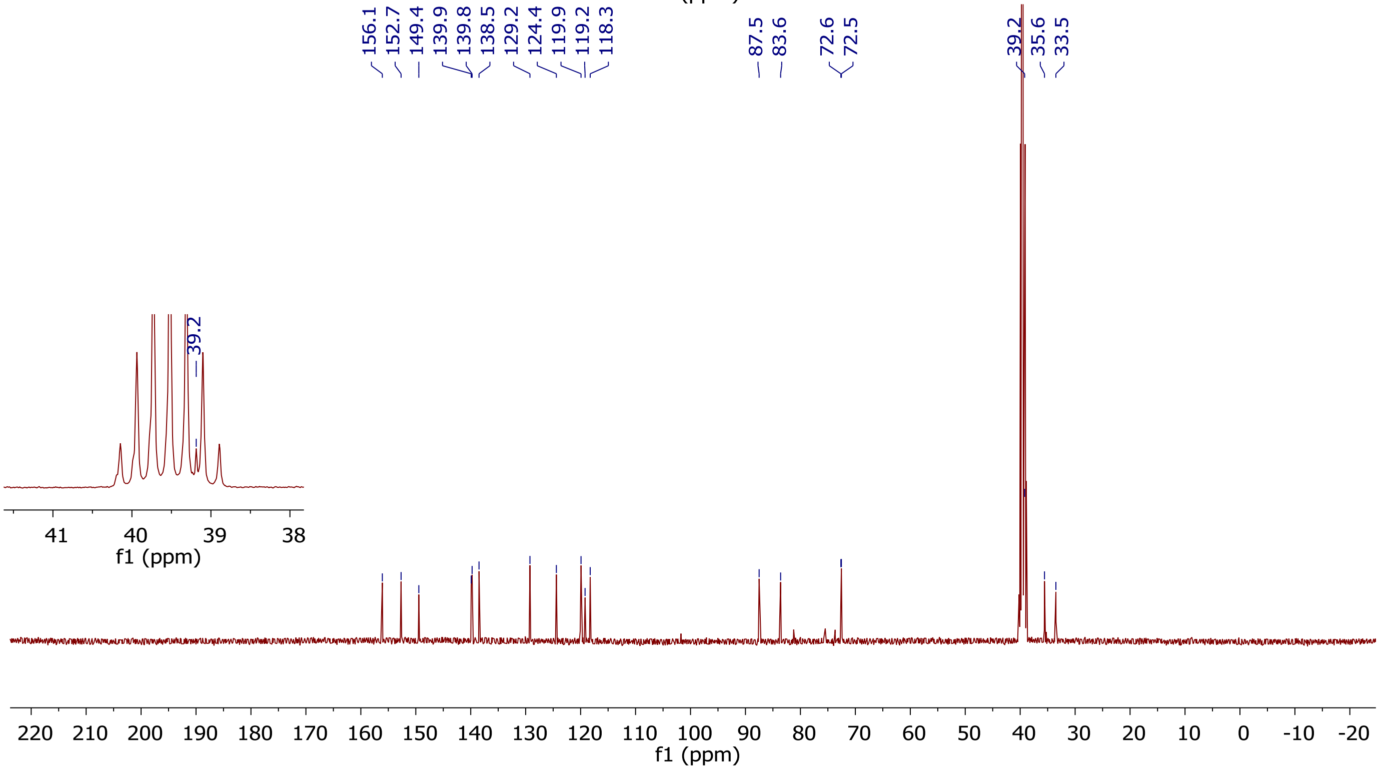
156.1
152.7
149.4
139.9
139.8
138.5
129.2
124.4
119.9
119.2
118.3
87.5
83.6
72.6
72.5
39.2
35.6
33.5
39.2
41 40 39 38
f1 (ppm)
220 210 200 190 180 170 160 150 140 130 120 110 100 90 80 70 60 50 40 30 20 10 0 -10 -20
f1 (ppm)

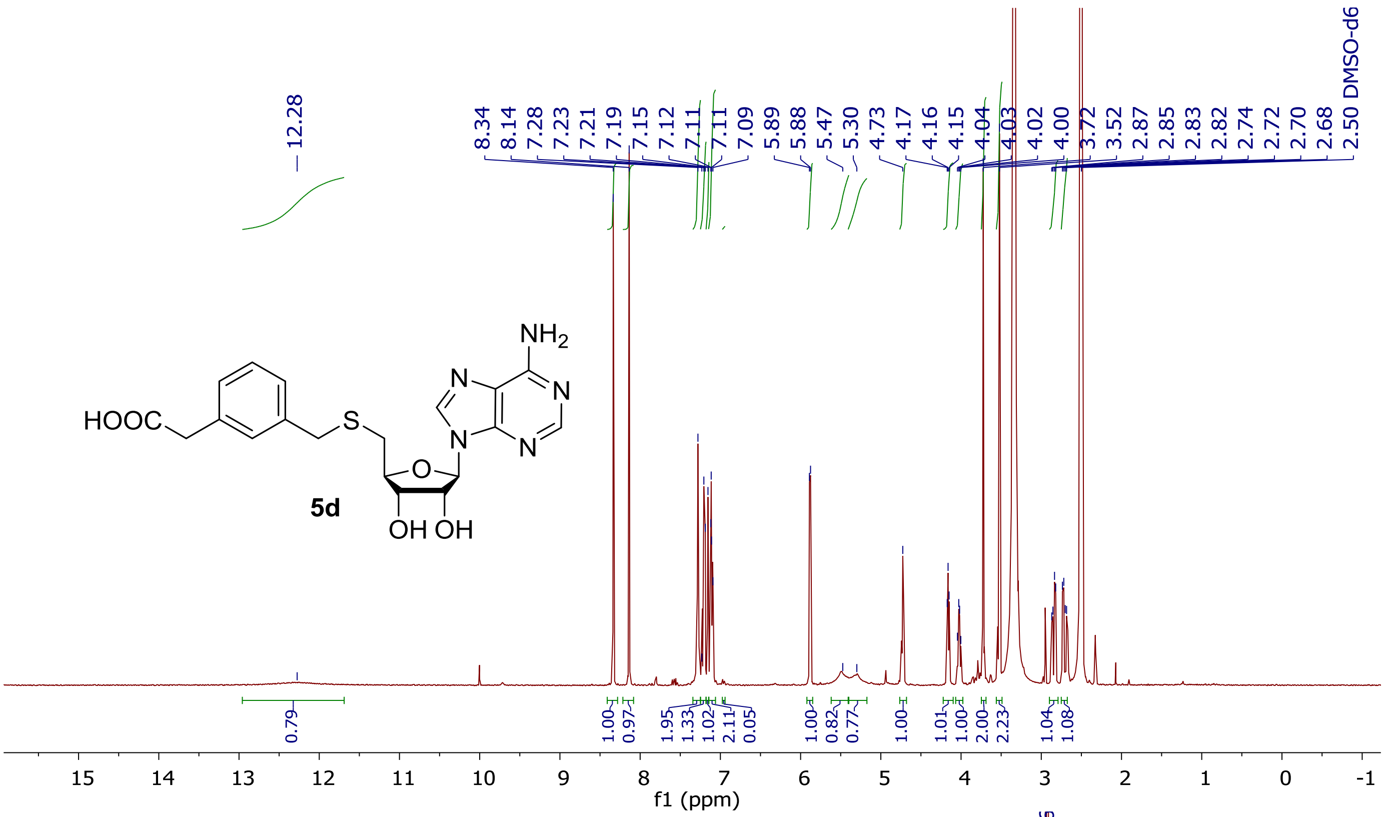

12.28
8.34
8.14
7.28
7.23
7.21
7.19
7.15
7.12
7.11
7.11
7.09
5.89
5.88
5.47
5.30
4.73
4.17
4.16
4.15
4.04
4.03
4.02
4.00
3.72
3.52
2.87
2.85
2.83
2.82
2.74
2.72
2.70
2.68
2.50 DMSO-d6
HOOC
S
O
N
N
N
N
NH2
OH OH
5d
0.79
1.00
0.97
1.95
1.33
1.02
2.11
0.05
1.00
0.82
0.77
1.00
1.01
1.00
2.00
2.23
1.04
1.08
f1 (ppm)

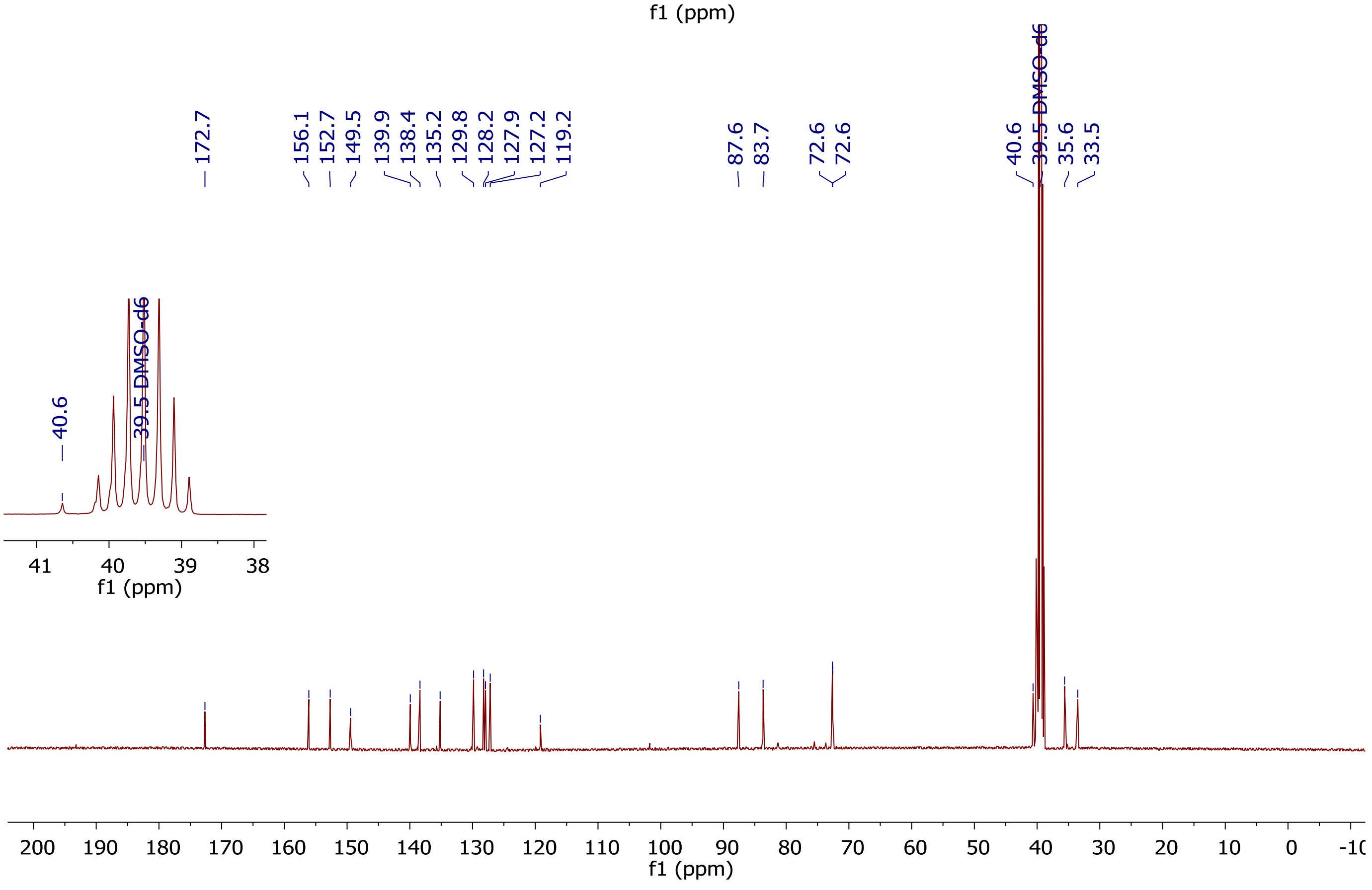

172.7
156.1
152.7
149.5
139.9
138.4
135.2
129.8
128.2
127.9
127.2
119.2
87.6
83.7
72.6
72.6
40.6
39.5 DMSO-d6
35.6
33.5
40.6
39.5 DMSO-d6
f1 (ppm)
f1 (ppm)

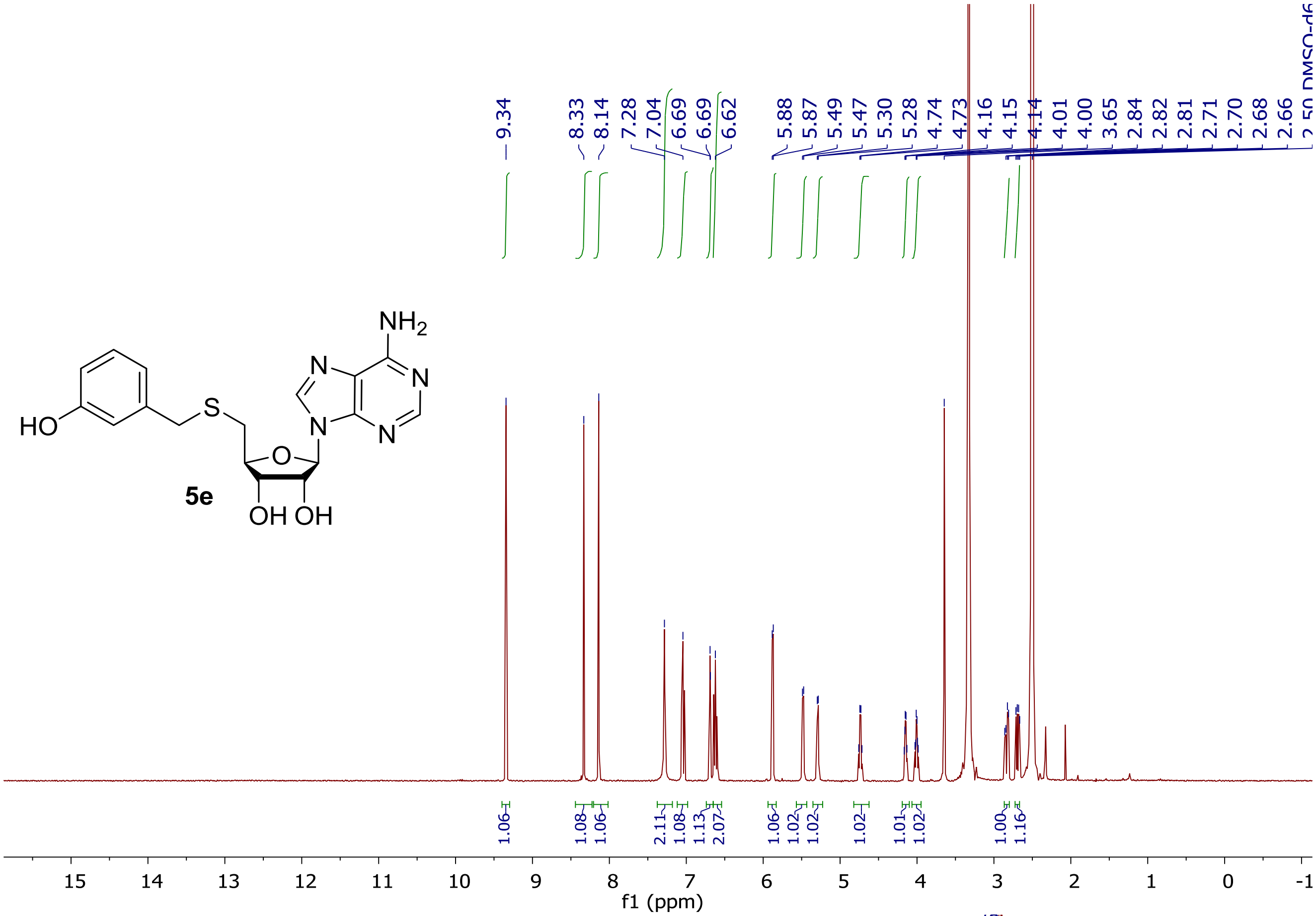

9.34
8.33
8.14
7.28
7.04
6.69
6.69
6.62
5.88
5.87
5.49
5.47
5.30
5.28
4.74
4.73
4.16
4.15
4.14
4.01
4.00
3.65
2.84
2.82
2.81
2.71
2.70
2.68
2.66
NH2
N
N
N
N
HO
S
O
OH OH
5e
1.06
1.08
1.06
2.11
1.08
1.13
2.07
1.06
1.02
1.02
1.02
1.01
1.02
1.00
1.16
15 14 13 12 11 10 9 8 7 6 5 4 3 2 1 0 -1
f1 (ppm)

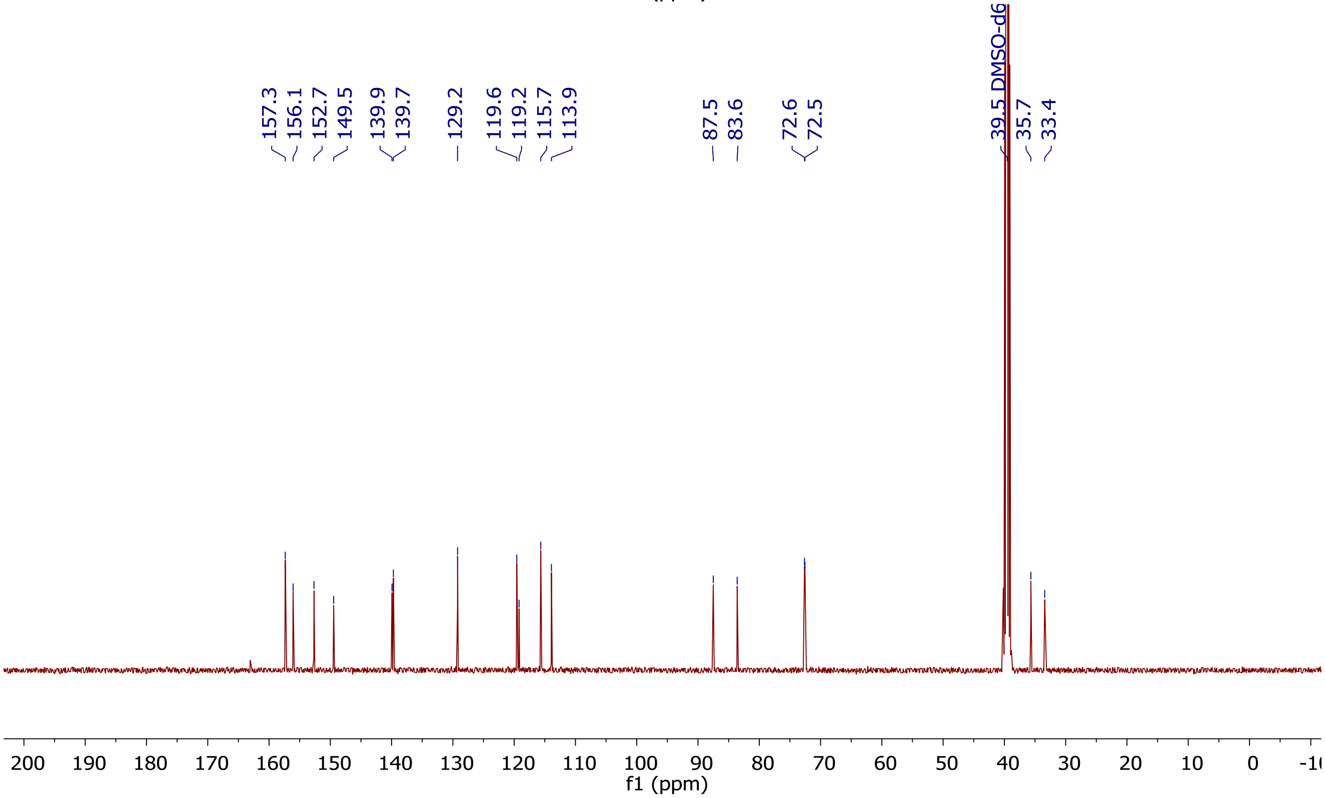

157.3
156.1
152.7
149.5
139.9
139.7
129.2
119.6
119.2
115.7
113.9
87.5
83.6
72.6
72.5
39.5 DMSO-d6
35.7
33.4
200 190 180 170 160 150 140 130 120 110 100 90 80 70 60 50 40 30 20 10 0
f1 (ppm)

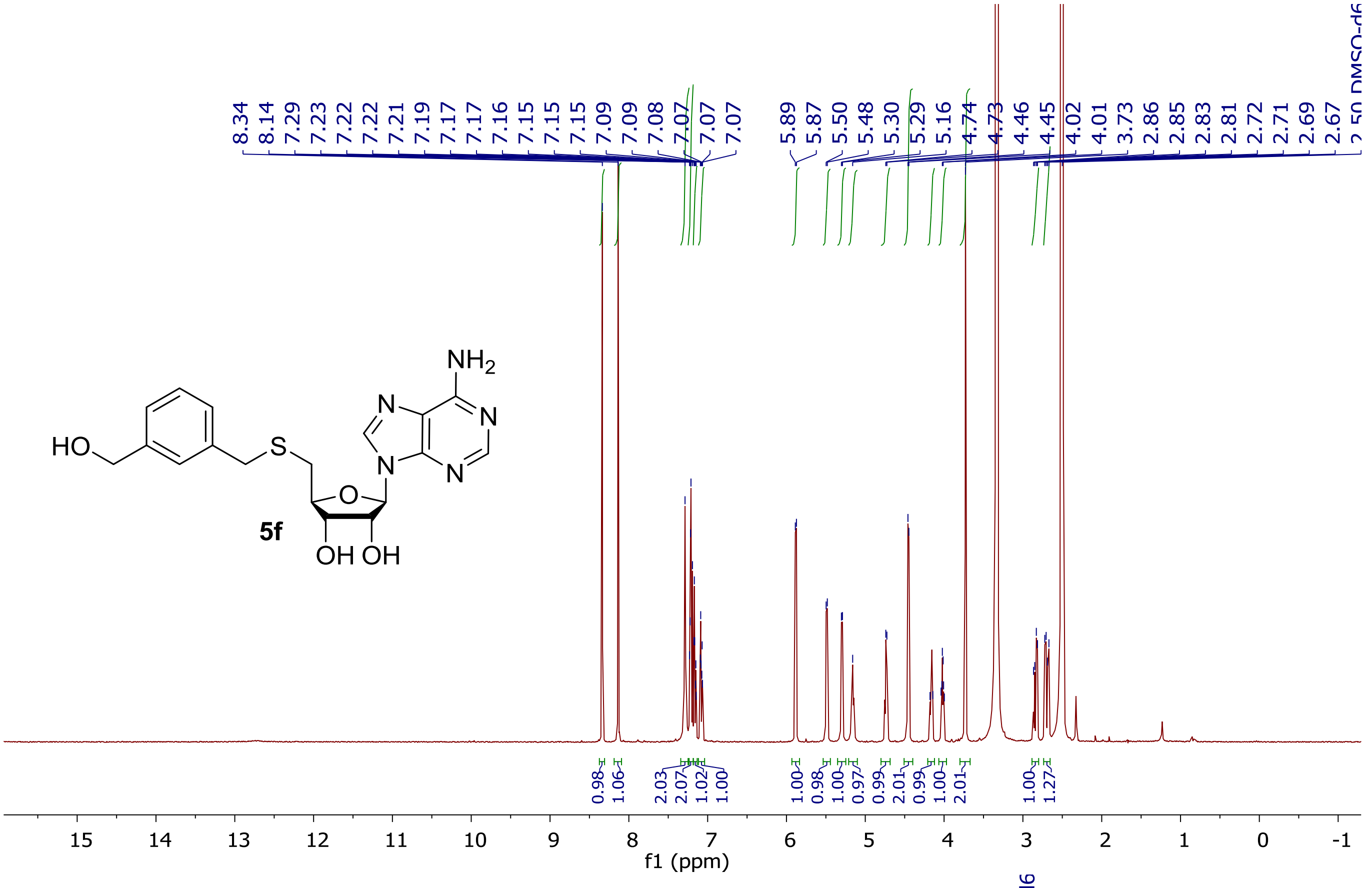
8.34 8.14 7.29 7.23 7.22 7.22 7.21 7.19 7.17 7.17 7.16 7.15 7.15 7.15 7.09 7.09 7.08 7.07 7.07 7.07
5.89 5.87 5.50 5.48 5.30 5.29 5.16 4.74 4.73 4.46 4.45 4.02 4.01 3.73 2.86 2.85 2.83 2.81 2.72 2.71 2.69 2.67 2.50 DMSO-d6
NH2
N
N
HO
S
N
N
N
O
5f
OH OH
0.98 1.06 2.03 2.07 1.02 1.00 1.00 0.98 1.00 0.97 0.99 2.01 0.99 1.00 2.01 1.00 1.27
15 14 13 12 11 10 9 8 7 6 5 4 3 2 1 0 -1
f1 (ppm)

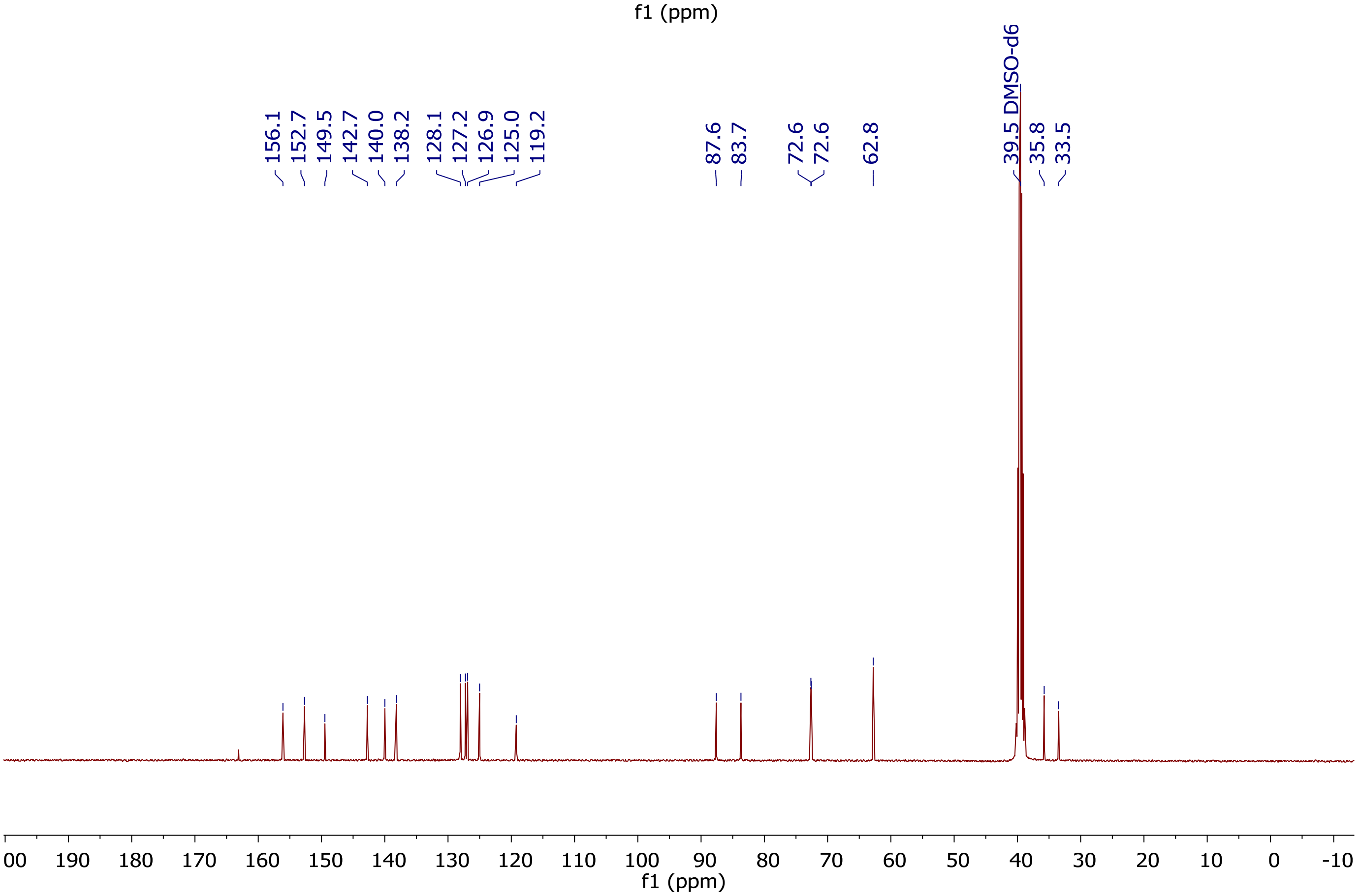
156.1 152.7 149.5 142.7 140.0 138.2 128.1 127.2 126.9 125.0 119.2
87.6 83.7 72.6 72.6 62.8
39.5 DMSO-d6 35.8 33.5
00 190 180 170 160 150 140 130 120 110 100 90 80 70 60 50 40 30 20 10 0 -10
f1 (ppm)

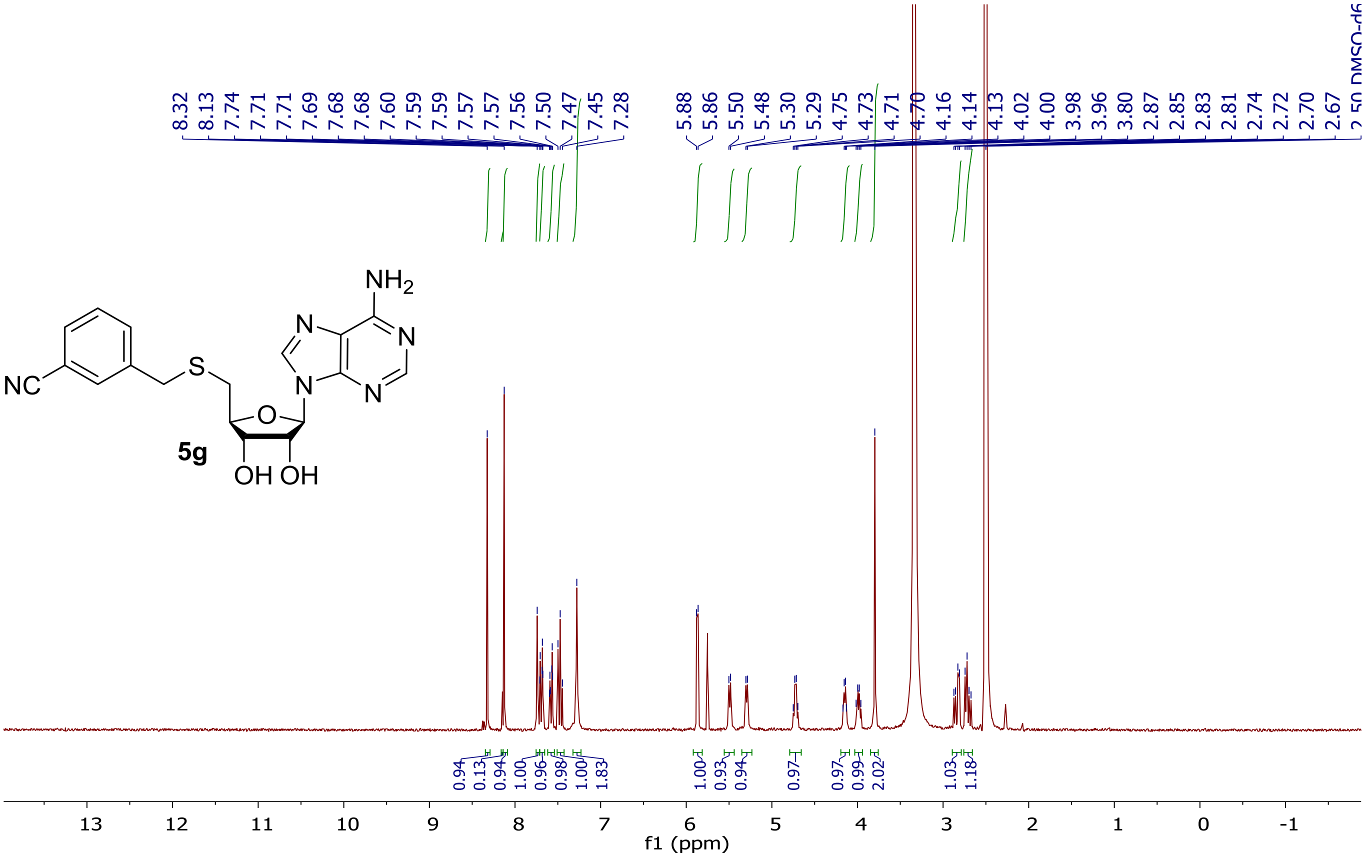
NH2
N
N
N
N
NC
S
O
OH OH

5g

8.32 8.13 7.74 7.71 7.71 7.69 7.68 7.68 7.60 7.59 7.59 7.57 7.57 7.56 7.50 7.47 7.45 7.28
5.88 5.86 5.50 5.48 5.30 5.29 4.75 4.73 4.71 4.70 4.16 4.14 4.13 4.02 4.00 3.98 3.96 3.80 2.87 2.85 2.83 2.81 2.74 2.72 2.70 2.67
0.94 0.13 0.94 1.00 0.96 0.98 1.00 1.83
1.00 0.93 0.94 0.97 0.97 0.99 2.02 1.03 1.18
13 12 11 10 9 8 7 6 5 4 3 2 1 0 -1
f1 (ppm)

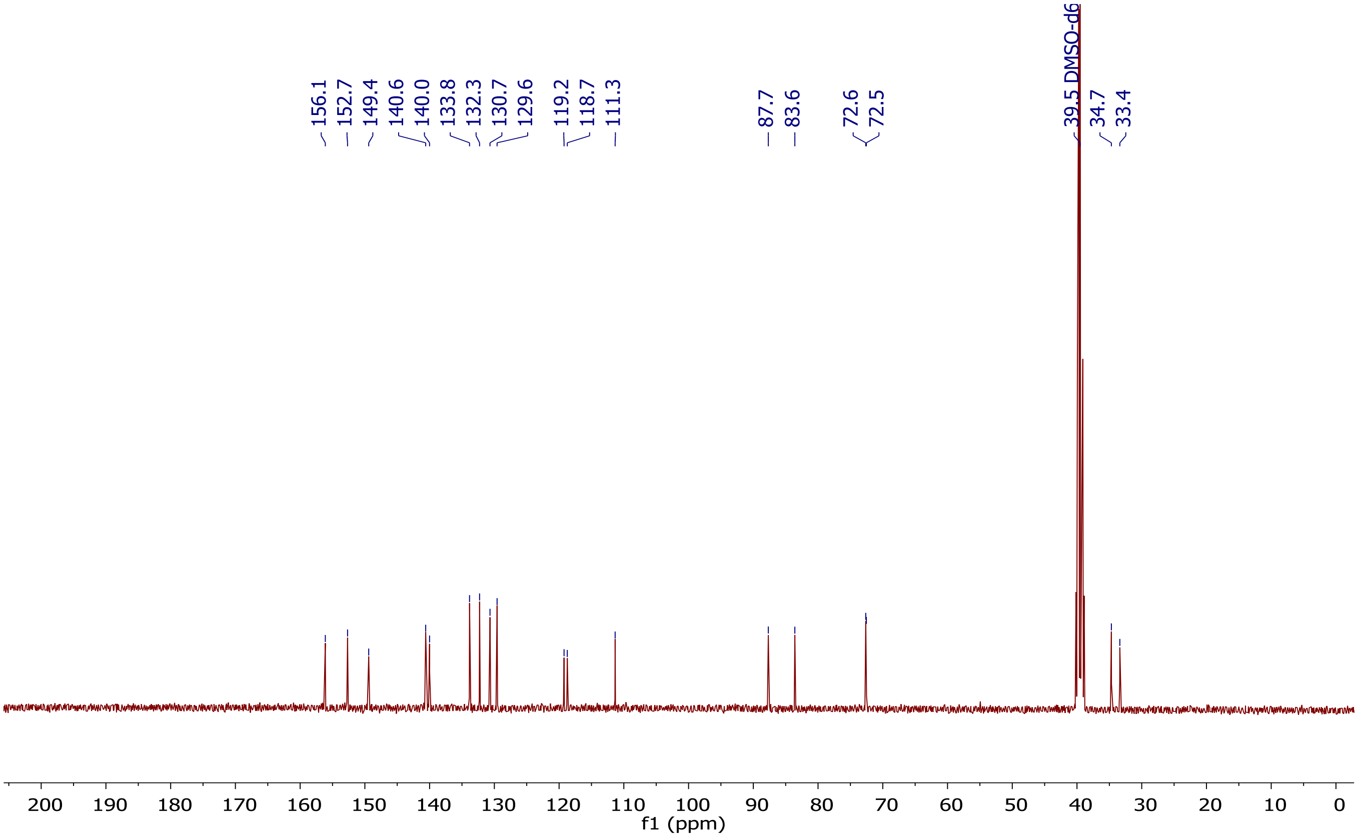
156.1 152.7 149.4 140.6 140.0 133.8 132.3 130.7 129.6 119.2 118.7 111.3
87.7 83.6 72.6 72.5
39.5 DMSO-d6 34.7 33.4
200 190 180 170 160 150 140 130 120 110 100 90 80 70 60 50 40 30 20 10 0
f1 (ppm)

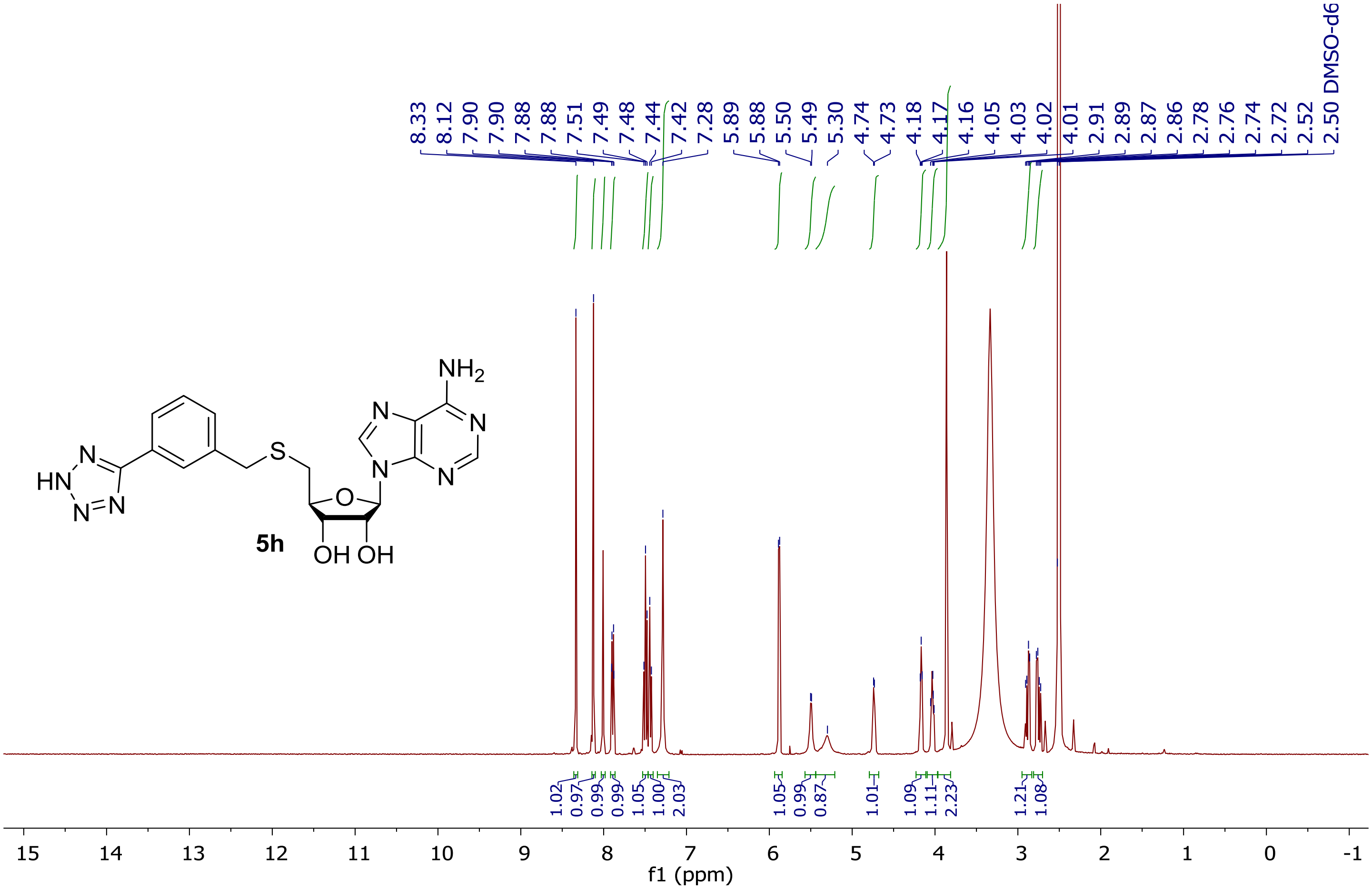
8.33 8.12 7.90 7.90 7.88 7.88 7.51 7.49 7.48 7.44 7.42 7.28 5.89 5.88 5.50 5.49 5.30 4.74 4.73 4.18 4.17 4.16 4.05 4.03 4.02 4.01 2.91 2.89 2.87 2.86 2.78 2.76 2.74 2.72 2.52 2.50 DMSO-d6
1.02 0.97 0.99 0.99 1.05 1.00 2.03 1.05 0.99 0.87 1.01 1.09 1.11 2.23 1.21 1.08
NH2
N
HN
O
S
OH OH
f1 (ppm)

5h

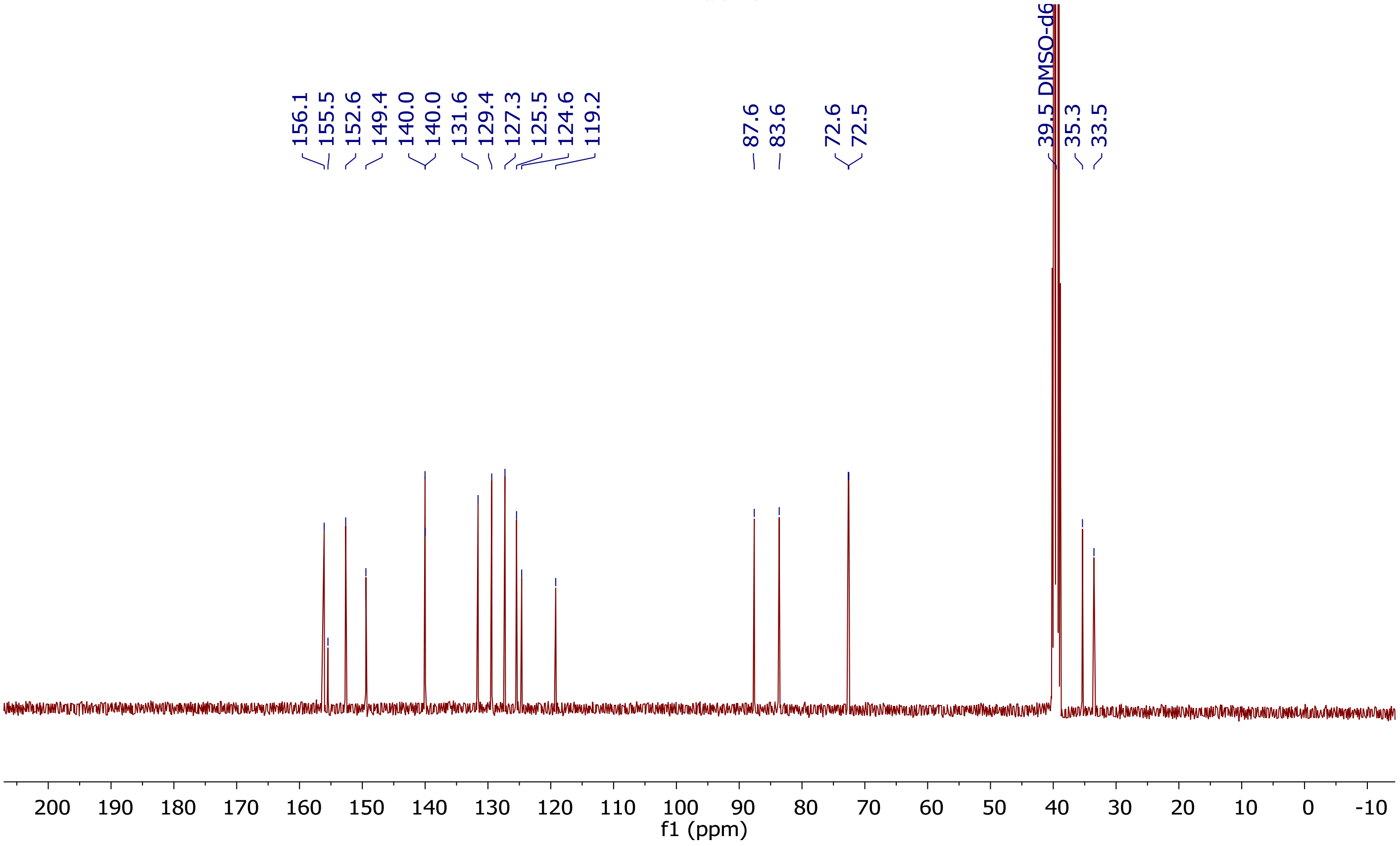
156.1 155.5 152.6 149.4 140.0 140.0 131.6 129.4 127.3 125.5 124.6 119.2 87.6 83.6 72.6 72.5 39.5 DMSO-d6 35.3 33.5
f1 (ppm)

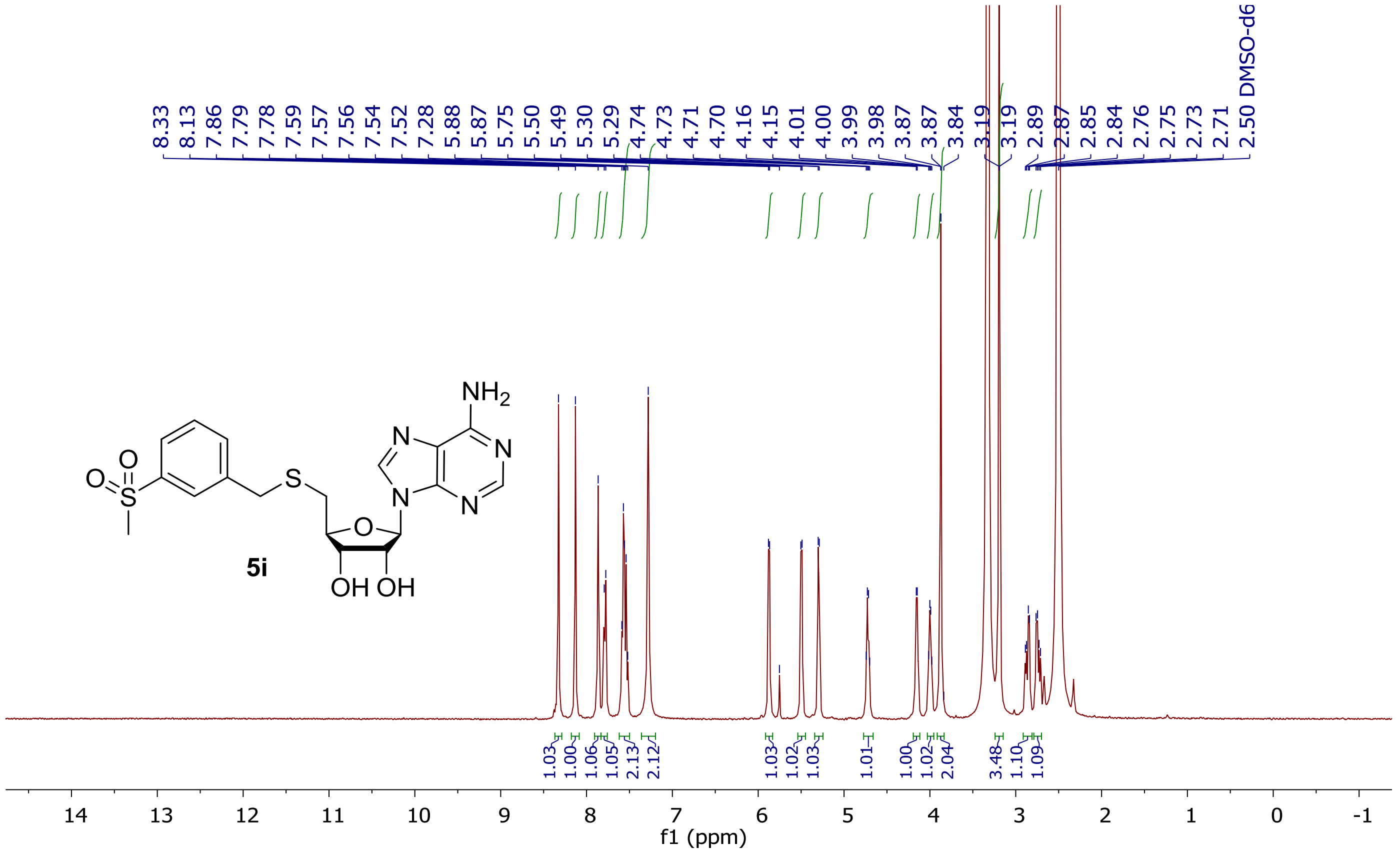
8.33 8.13 7.86 7.79 7.78 7.59 7.57 7.56 7.54 7.52 7.28 5.88 5.87 5.75 5.50 5.49 5.30 5.29 4.74 4.73 4.71 4.70 4.16 4.15 4.01 4.00 3.99 3.98 3.87 3.87 3.84 3.19 3.19 2.89 2.87 2.85 2.84 2.76 2.75 2.73 2.71 2.50 DMSO-d6
NH2
N
N
N
N
O
S
O
O
S
OH OH
5i
1.03 1.00 1.06 1.05 2.13 2.12 1.03 1.02 1.03 1.01 1.00 1.02 2.04 3.48 1.10 1.09
14 13 12 11 10 9 8 7 6 5 4 3 2 1 0 -1
f1 (ppm)

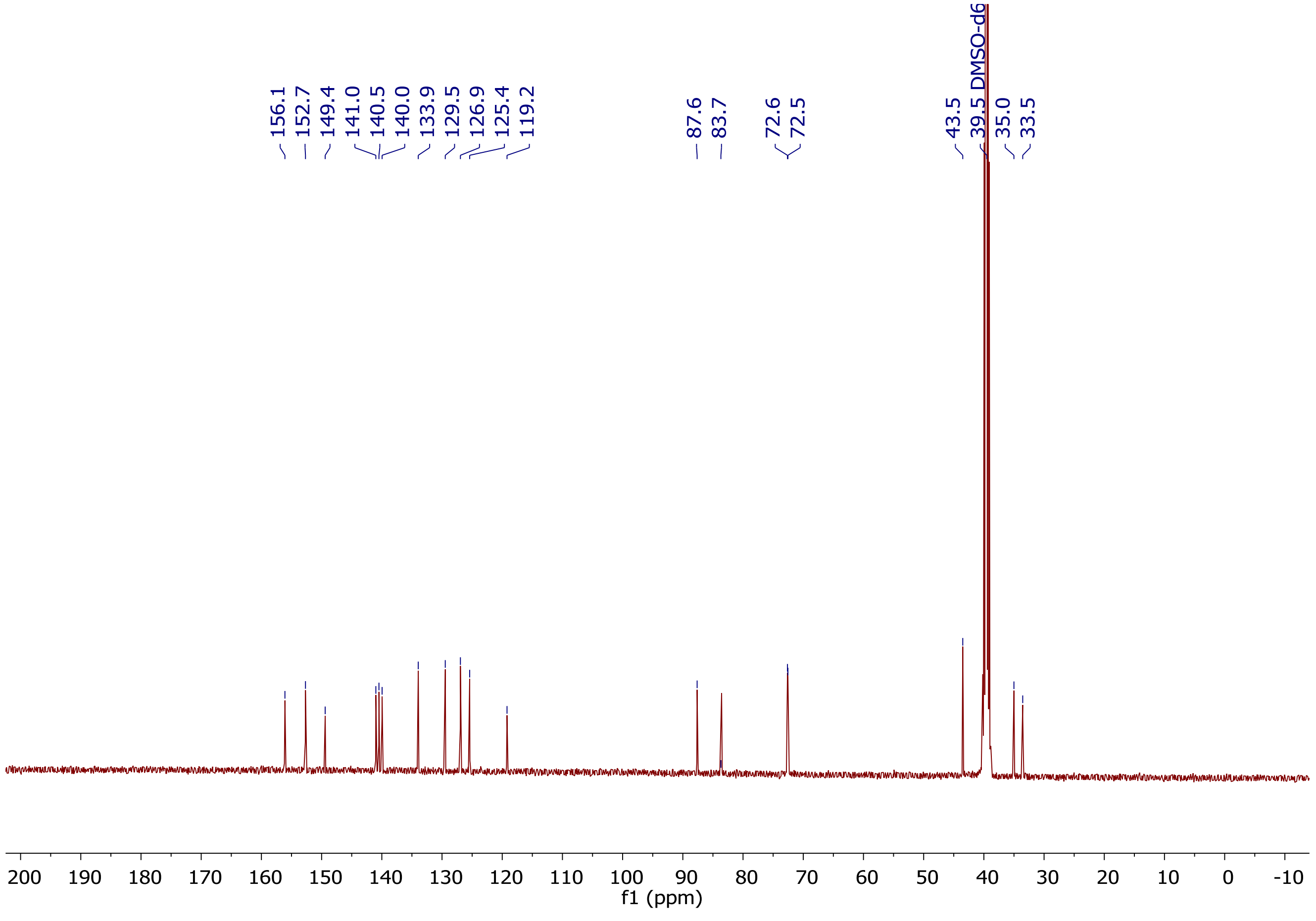
156.1 152.7 149.4 141.0 140.5 140.0 133.9 129.5 126.9 125.4 119.2
87.6 83.7
72.6 72.5
43.5 39.5 DMSO-d6 35.0 33.5
200 190 180 170 160 150 140 130 120 110 100 90 80 70 60 50 40 30 20 10 0 -10
f1 (ppm)

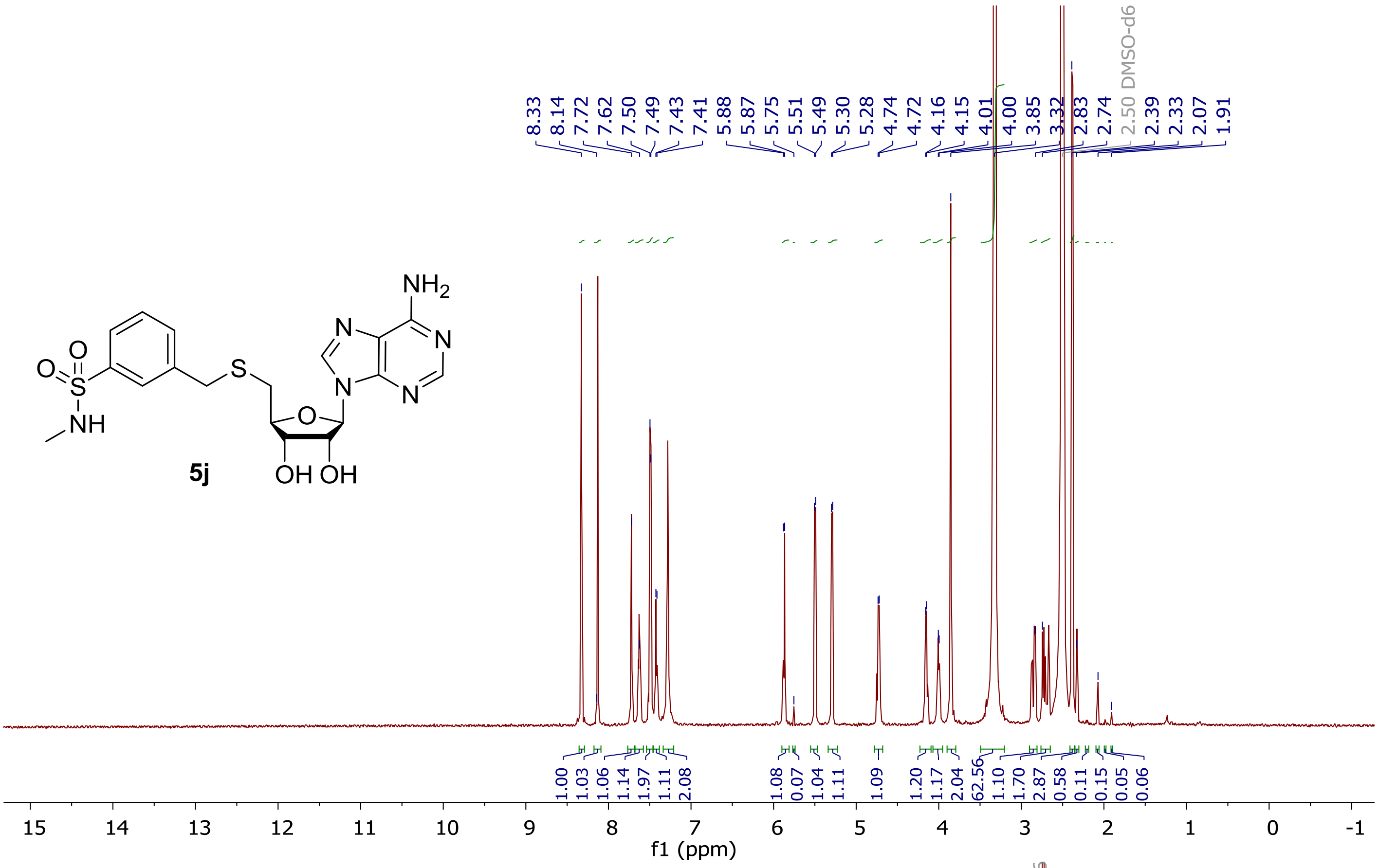
5j
8.33 8.14 7.72 7.62 7.50 7.49 7.43 7.41 5.88 5.87 5.75 5.51 5.49 5.30 5.28 4.74 4.72 4.16 4.15 4.01 4.00 3.85 3.32 2.83 2.74 2.50 DMSO-d6 2.39 2.33 2.07 1.91
1.00 1.03 1.06 1.14 1.97 1.11 2.08 1.08 0.07 1.04 1.11 1.09 1.20 1.17 2.04 62.56 1.10 1.70 2.87 0.58 0.11 0.15 0.05 0.06
f1 (ppm)

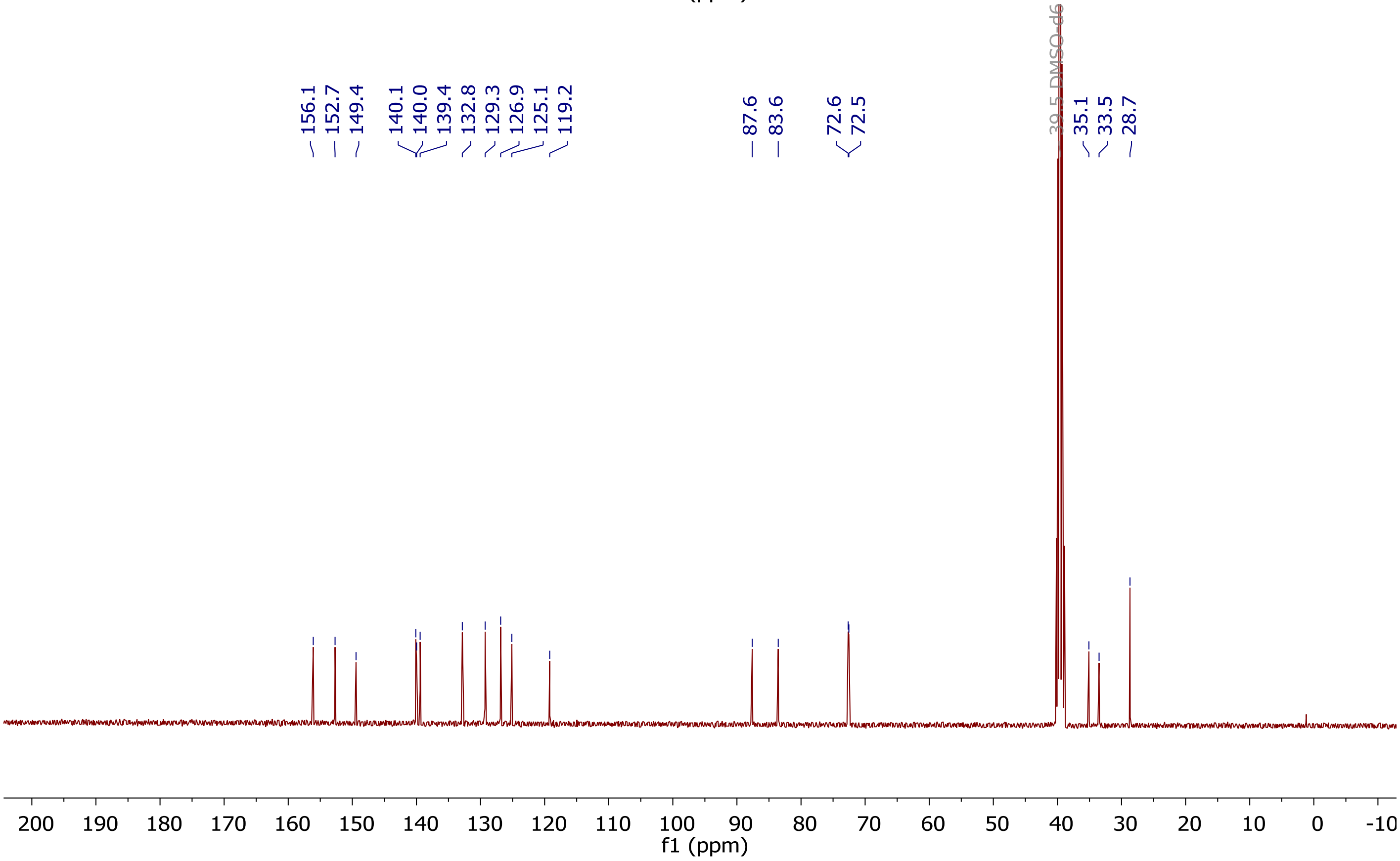
156.1 152.7 149.4 140.1 140.0 139.4 132.8 129.3 126.9 125.1 119.2 87.6 83.6 72.6 72.5 39.5 DMSO-d6 35.1 33.5 28.7
f1 (ppm)

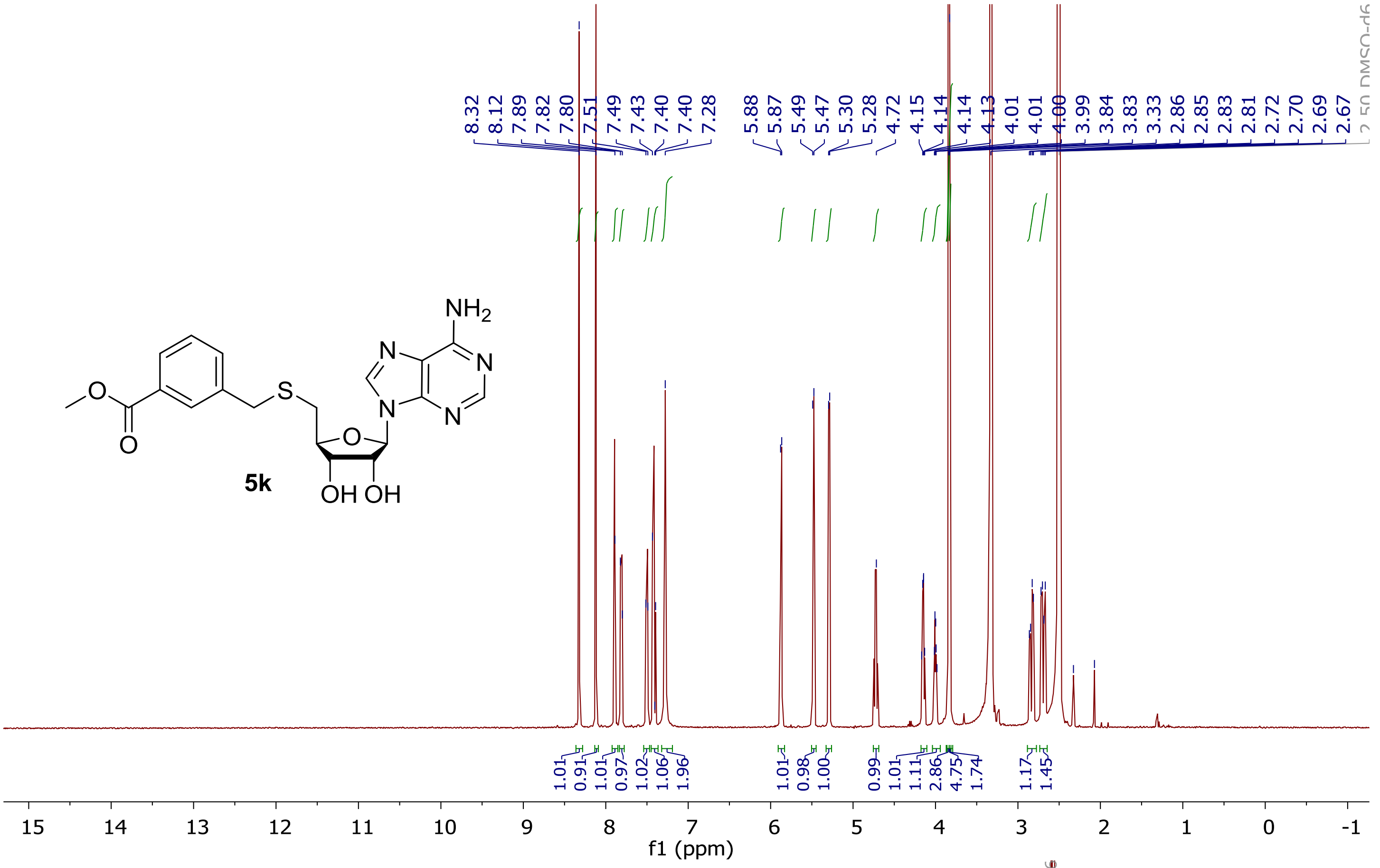
8.32
8.12
7.89
7.82
7.80
7.51
7.49
7.43
7.40
7.40
7.28
5.88
5.87
5.49
5.47
5.30
5.28
4.72
4.15
4.14
4.14
4.13
4.01
4.01
4.00
3.99
3.84
3.83
3.33
2.86
2.85
2.83
2.81
2.72
2.70
2.69
2.67
NH2
N
N
N
N
O
O
S
O
OH OH
5k
1.01
0.91
1.01
0.97
1.02
1.06
1.96
1.01
0.98
1.00
0.99
1.01
1.11
2.86
4.75
1.74
1.17
1.45
15 14 13 12 11 10 9 8 7 6 5 4 3 2 1 0 -1
f1 (ppm)

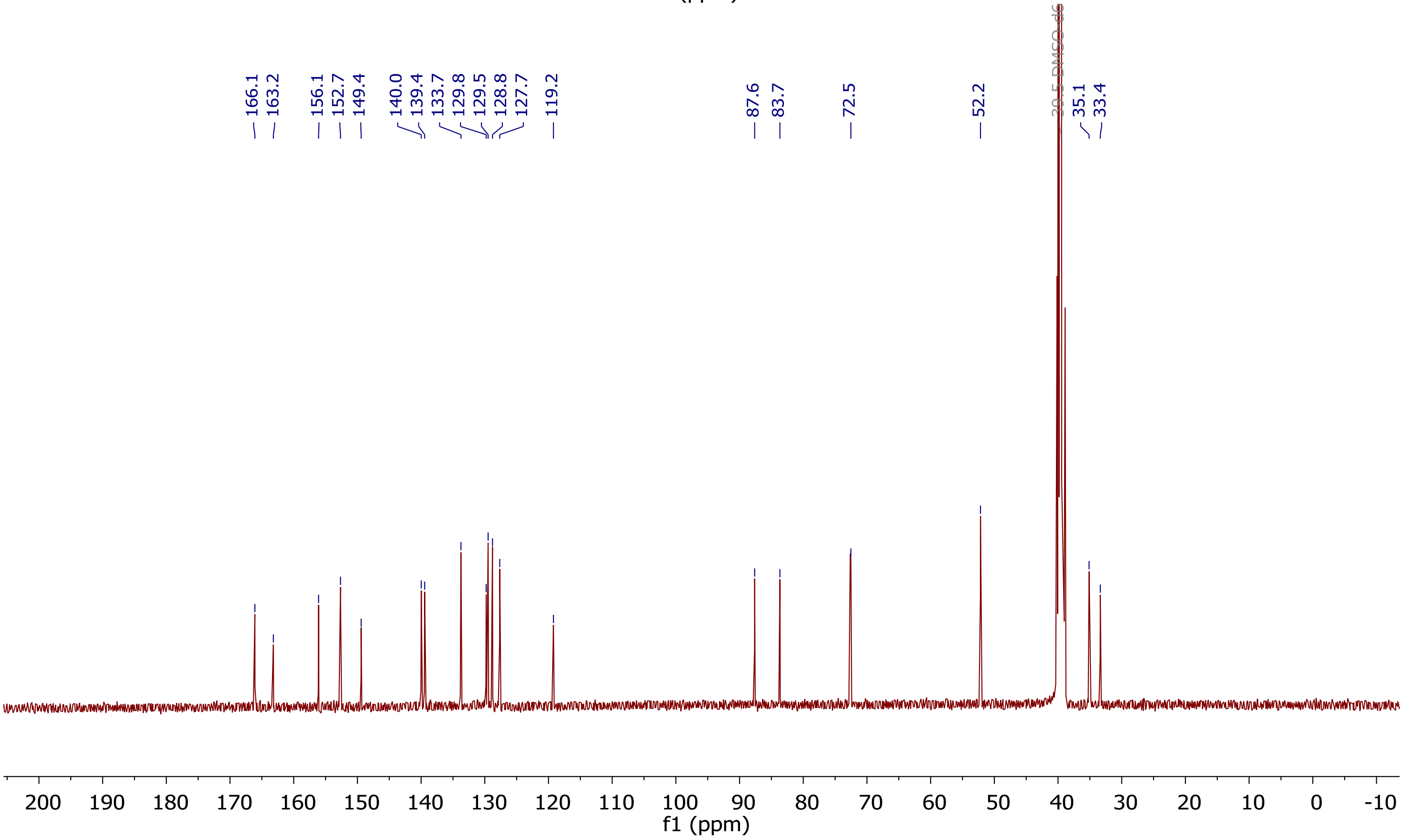
166.1
163.2
156.1
152.7
149.4
140.0
139.4
133.7
129.8
129.5
128.8
127.7
119.2
87.6
83.7
72.5
52.2
35.1
33.4
200 190 180 170 160 150 140 130 120 110 100 90 80 70 60 50 40 30 20 10 0 -10
f1 (ppm)

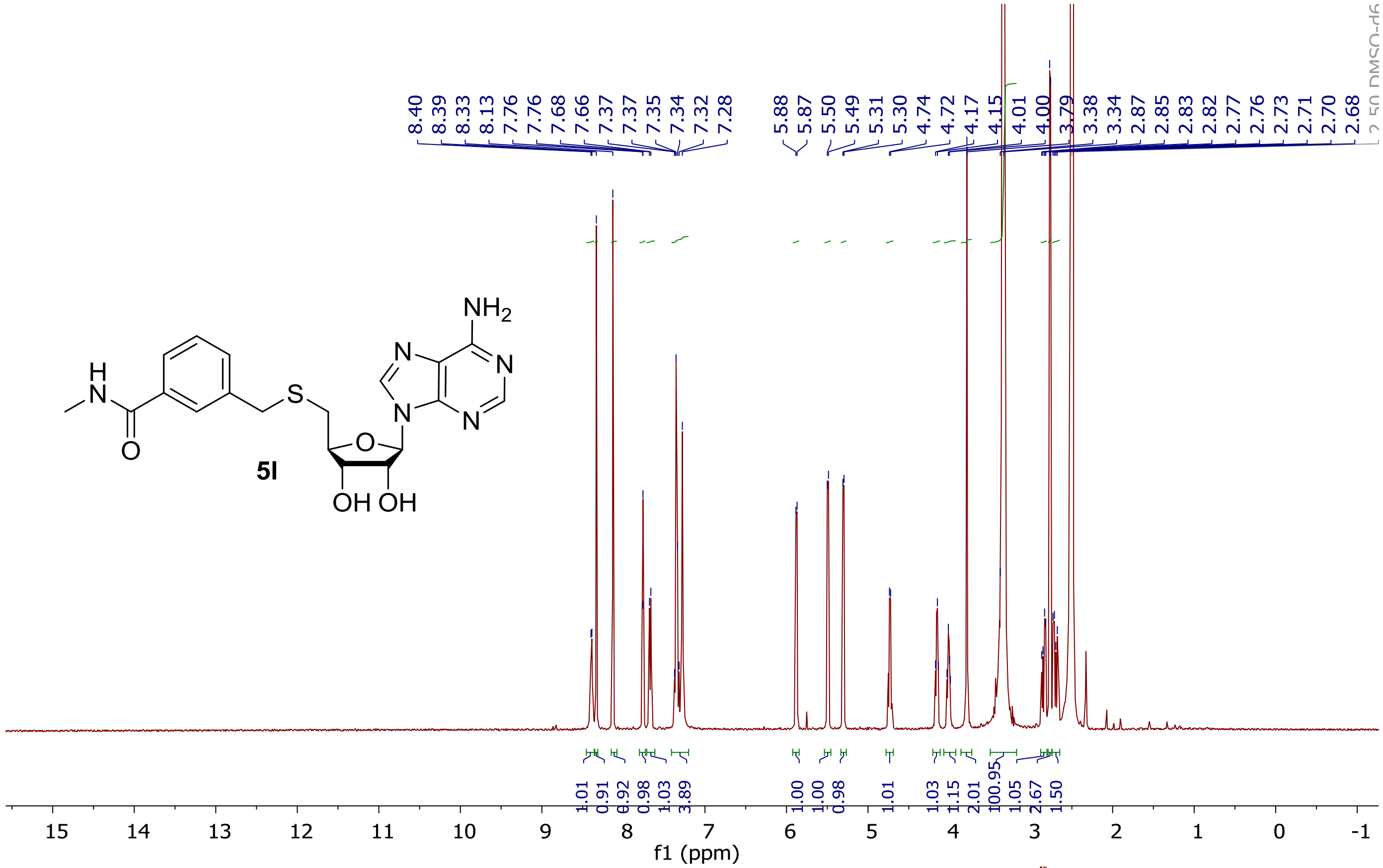
8.40
8.39
8.33
8.13
7.76
7.76
7.68
7.66
7.37
7.37
7.35
7.34
7.32
7.28
5.88
5.87
5.50
5.49
5.31
5.30
4.74
4.72
4.17
4.15
4.01
4.00
3.79
3.38
3.34
2.87
2.85
2.83
2.82
2.77
2.76
2.73
2.71
2.70
2.68
2.50 DMSO-d6
NH2
N
N
N
N
S
H
N
O
O
OH OH
5l
1.01
0.91
0.92
0.98
1.03
3.89
1.00
1.00
0.98
1.01
1.03
1.15
2.01
100.95
1.05
2.67
1.50
f1 (ppm)

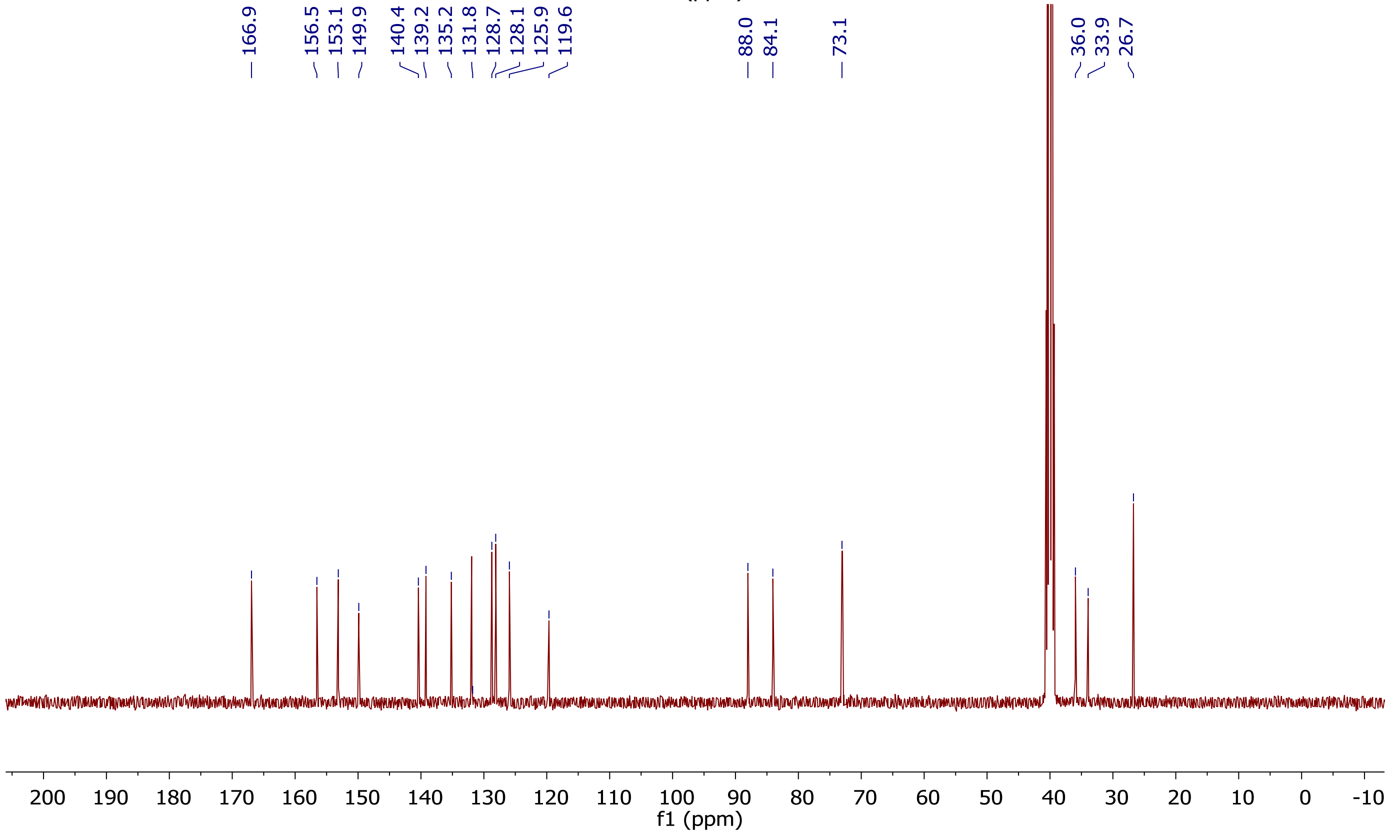
166.9
156.5
153.1
149.9
140.4
139.2
135.2
131.8
128.7
128.1
125.9
119.6
88.0
84.1
73.1
36.0
33.9
26.7
f1 (ppm)

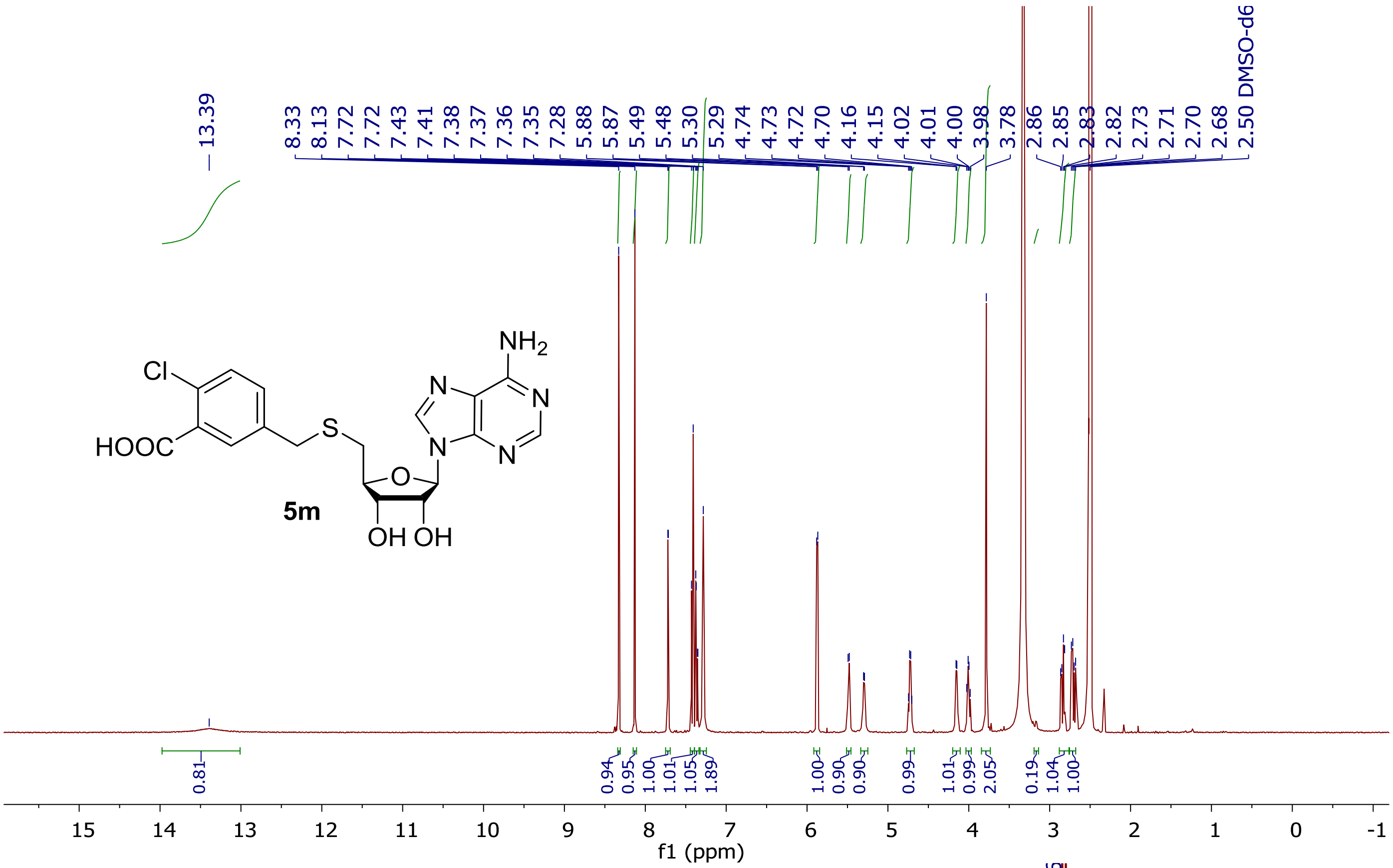
13.39
8.33 8.13 7.72 7.72 7.43 7.41 7.38 7.37 7.36 7.35 7.28 5.88 5.87 5.49 5.48 5.30 5.29 4.74 4.73 4.72 4.70 4.16 4.15 4.02 4.01 4.00 3.98 3.78 2.86 2.85 2.83 2.82 2.73 2.71 2.70 2.68 2.50 DMSO-d6
Cl
HOOC
S
O
N
NH2
OH OH
5m
0.81
0.94 0.95 1.00 1.01 1.05 1.89 1.00 0.90 0.90 0.99 1.01 0.99 2.05 0.19 1.04 1.00
15 14 13 12 11 10 9 8 7 6 5 4 3 2 1 0 -1
f1 (ppm)

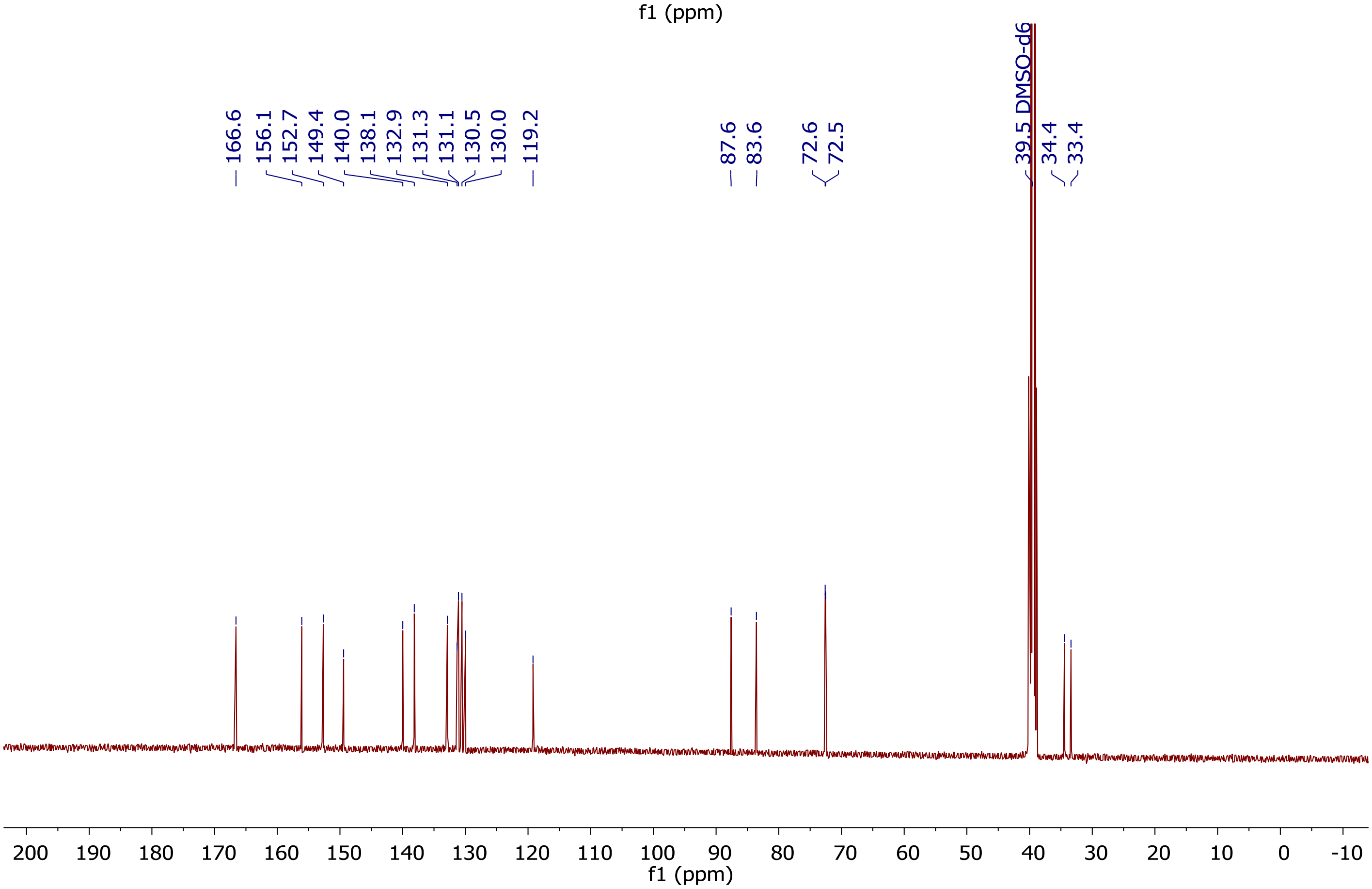
166.6 156.1 152.7 149.4 140.0 138.1 132.9 131.3 131.1 130.5 130.0 119.2
87.6 83.6
72.6 72.5
39.5 DMSO-d6 34.4 33.4
200 190 180 170 160 150 140 130 120 110 100 90 80 70 60 50 40 30 20 10 0 -10
f1 (ppm)

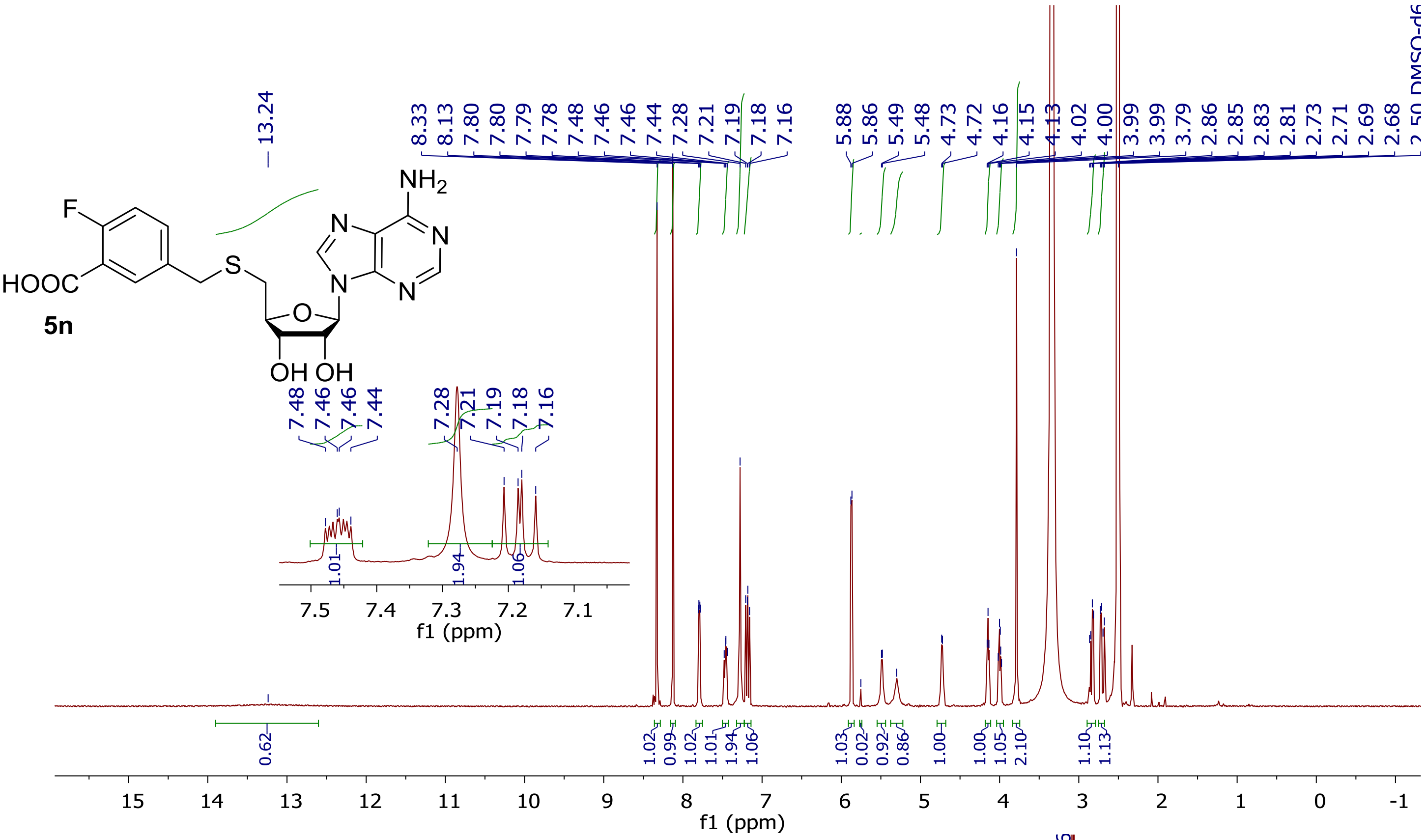
NH2
F
HOOC
S
N
O
OH OH
5n
13.24
8.33 8.13 7.80 7.80 7.79 7.78 7.48 7.46 7.46 7.44 7.28 7.21 7.19 7.18 7.16
5.88 5.86 5.49 5.48 4.73 4.72 4.16 4.15 4.13 4.02 4.00 3.99 3.99 3.79 2.86 2.85 2.83 2.81 2.73 2.71 2.69 2.68
7.48 7.46 7.46 7.44
7.28 7.21 7.19 7.18 7.16
1.01
1.94
1.06
f1 (ppm)
0.62
1.02 0.99 1.02 1.01 1.94 1.06
1.03 0.02 0.92 0.86
1.00
1.00 1.05 2.10
1.10 1.13
f1 (ppm)

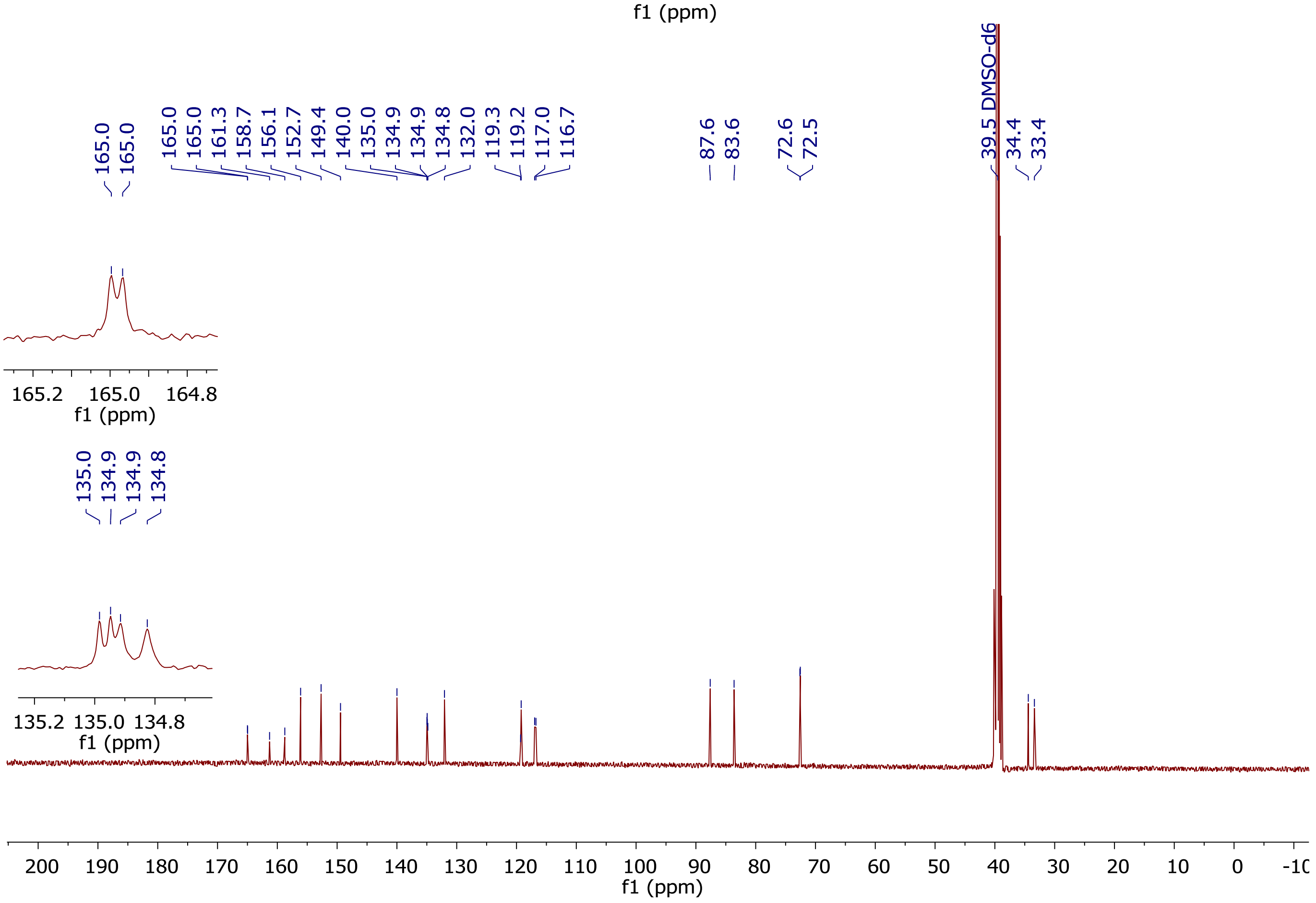
165.0 165.0
165.0 165.0 161.3 158.7 156.1 152.7 149.4 140.0 135.0 134.9 134.9 134.8 132.0 119.3 119.2 117.0 116.7
87.6 83.6
72.6 72.5
39.5 DMSO-d6
34.4 33.4
f1 (ppm)
135.0 134.9 134.9 134.8
f1 (ppm)
f1 (ppm)

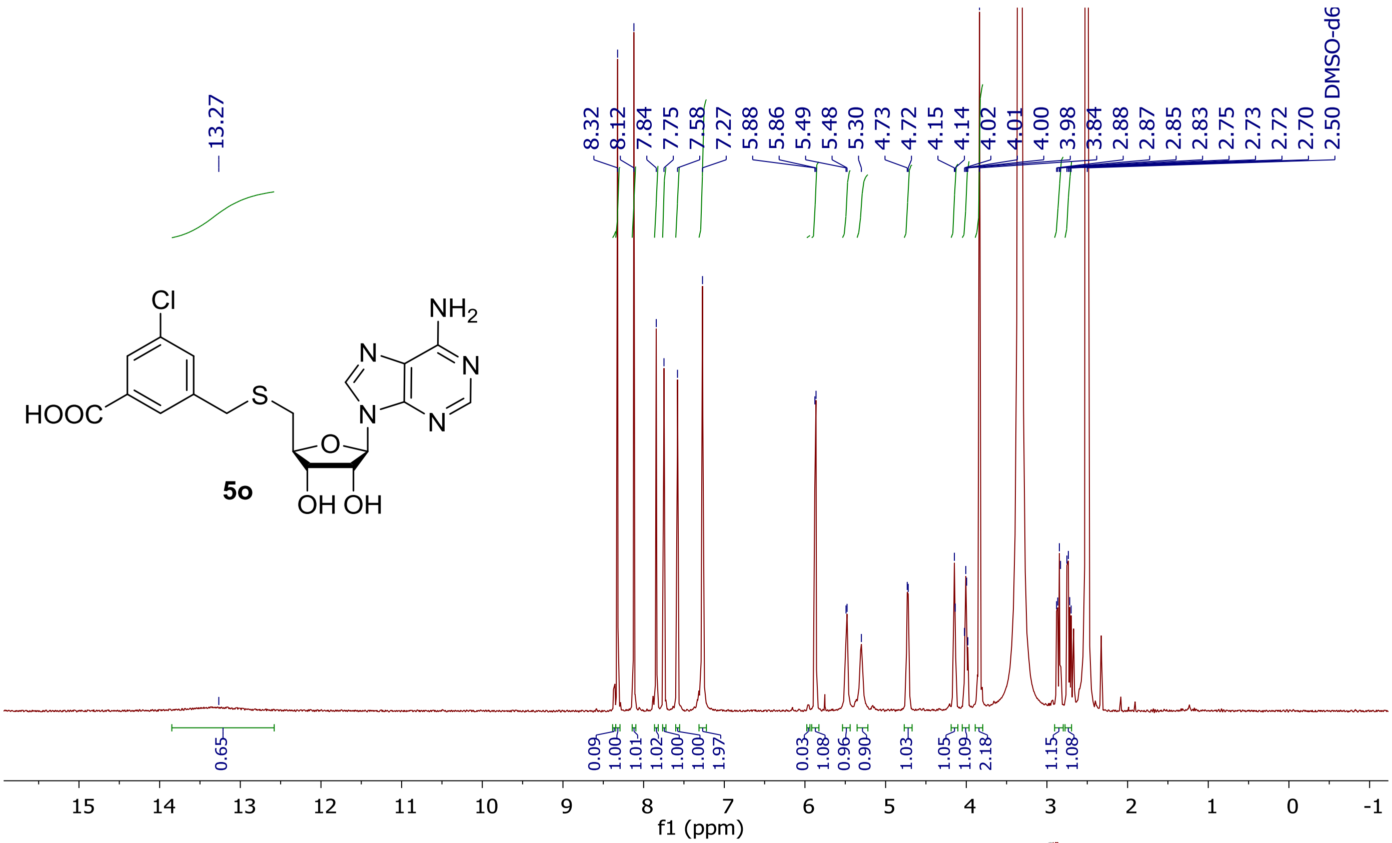

KFSL2-OBV-216.11.fid

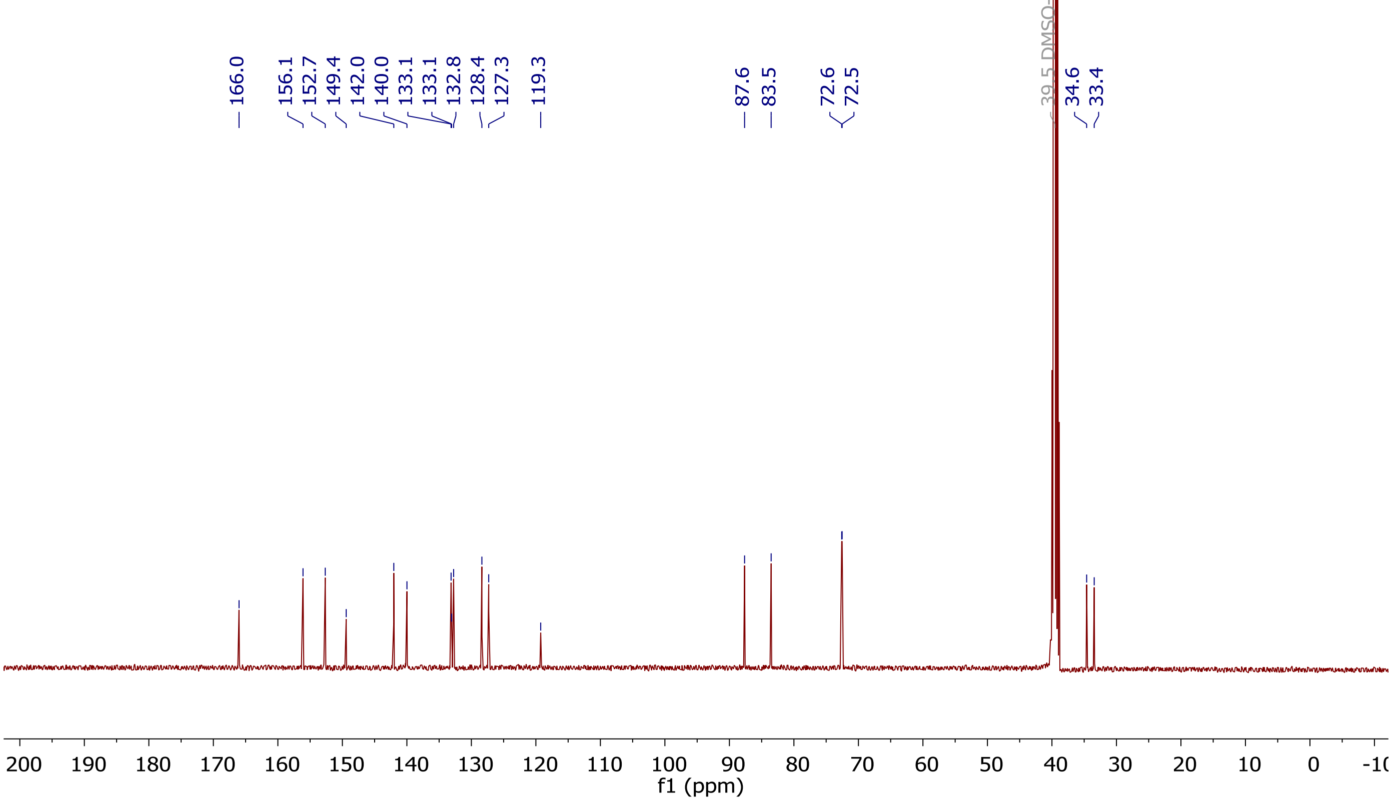

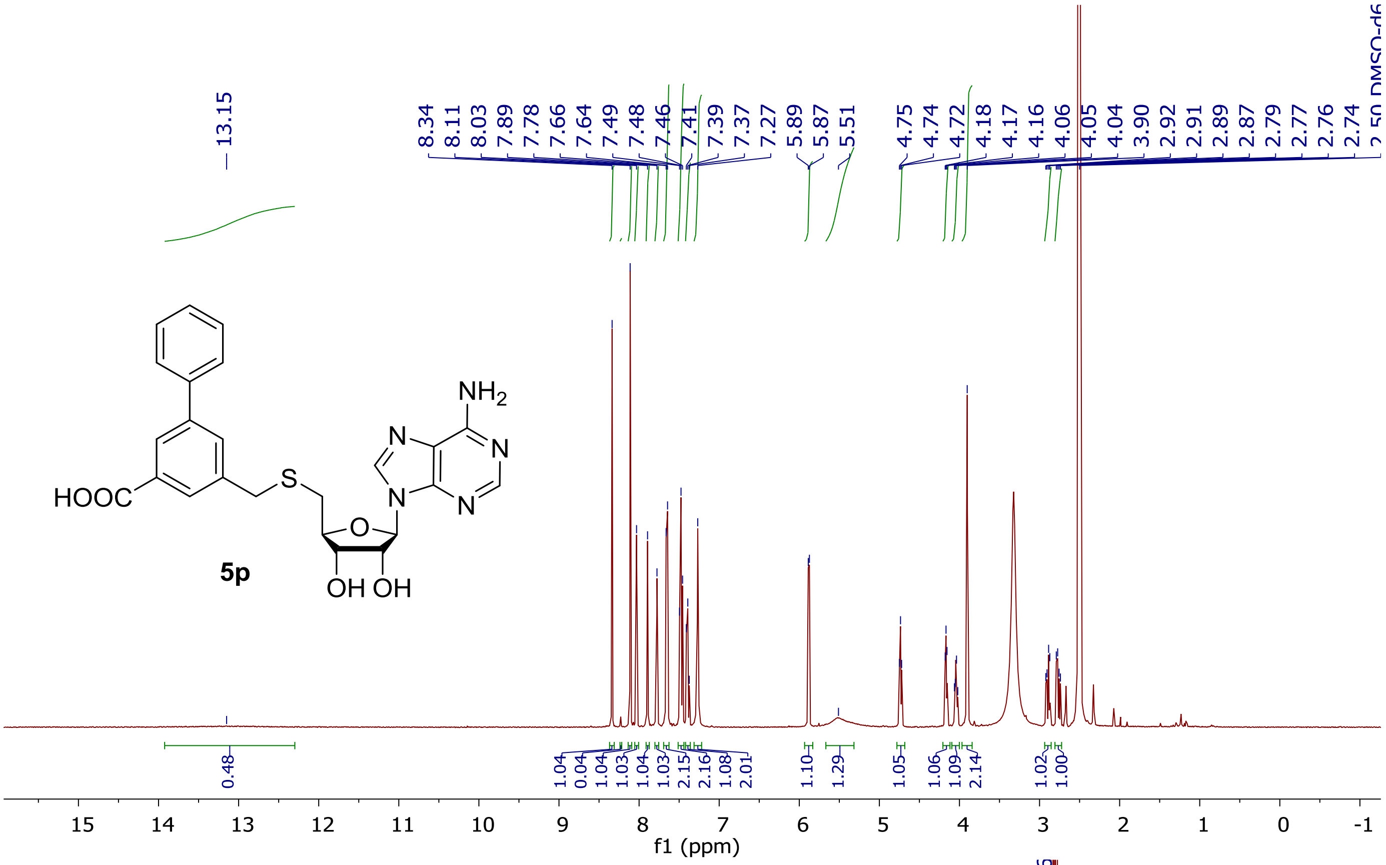
13.15
8.34
8.11
8.03
7.89
7.78
7.66
7.64
7.49
7.48
7.46
7.41
7.39
7.37
7.27
5.89
5.87
5.51
4.75
4.74
4.72
4.18
4.17
4.16
4.06
4.05
4.04
3.90
2.92
2.91
2.89
2.87
2.79
2.77
2.76
2.74
2.50 DMSO-d6
NH2
N
N
N
N
HOOC
S
O
OH OH
5p
0.48
1.04
0.04
1.04
1.03
1.04
1.03
2.15
2.16
1.08
2.01
1.10
1.29
1.05
1.06
1.09
2.14
1.02
1.00
15
14
13
12
11
10
9
8
7
6
5
4
3
2
1
0
-1
f1 (ppm)

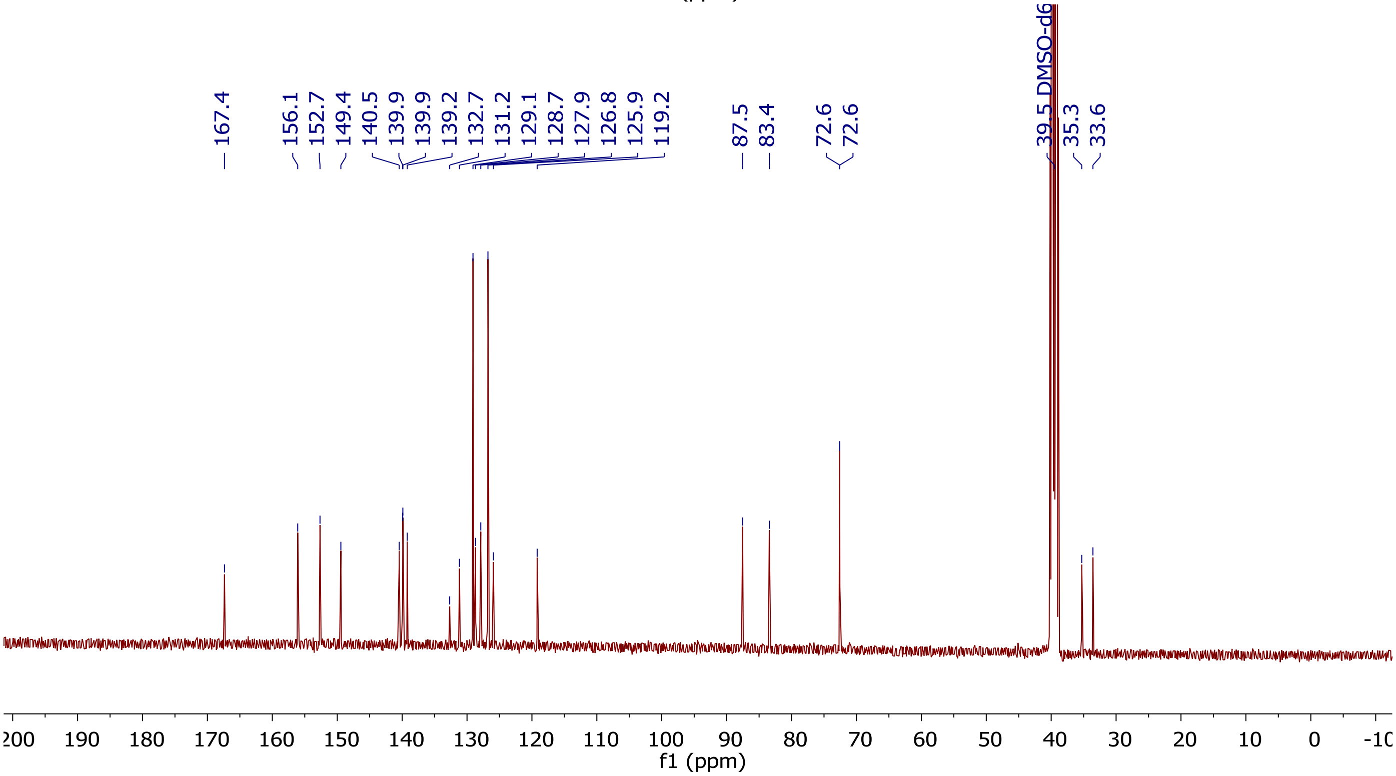
167.4
156.1
152.7
149.4
140.5
139.9
139.9
139.2
132.7
131.2
129.1
128.7
127.9
126.8
125.9
119.2
87.5
83.4
72.6
72.6
39.5 DMSO-d6
35.3
33.6
200
190
180
170
160
150
140
130
120
110
100
90
80
70
60
50
40
30
20
10
0
-10
f1 (ppm)

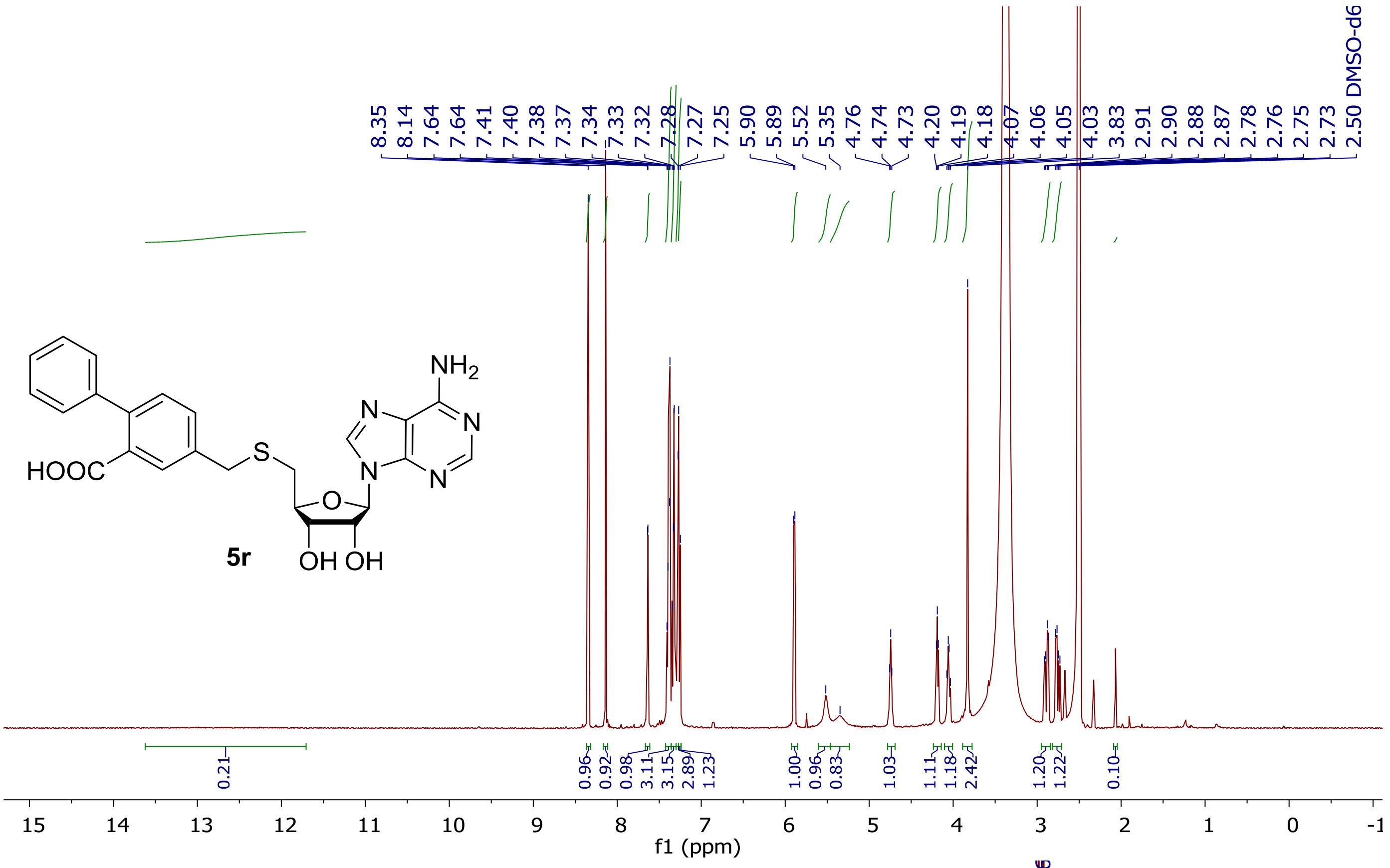
8.35
8.14
7.64
7.64
7.41
7.40
7.38
7.37
7.34
7.33
7.32
7.28
7.27
7.25
5.90
5.89
5.52
5.35
4.76
4.74
4.73
4.20
4.19
4.18
4.07
4.06
4.05
4.03
3.83
2.91
2.90
2.88
2.87
2.78
2.76
2.75
2.73
2.50 DMSO-d6
NH2
N
HOOC
S
O
OH OH
5r
0.21
0.96
0.92
0.98
3.11
3.15
2.89
1.23
1.00
0.96
0.83
1.03
1.11
1.18
2.42
1.20
1.22
0.10
15 14 13 12 11 10 9 8 7 6 5 4 3 2 1 0 -1
f1 (ppm)

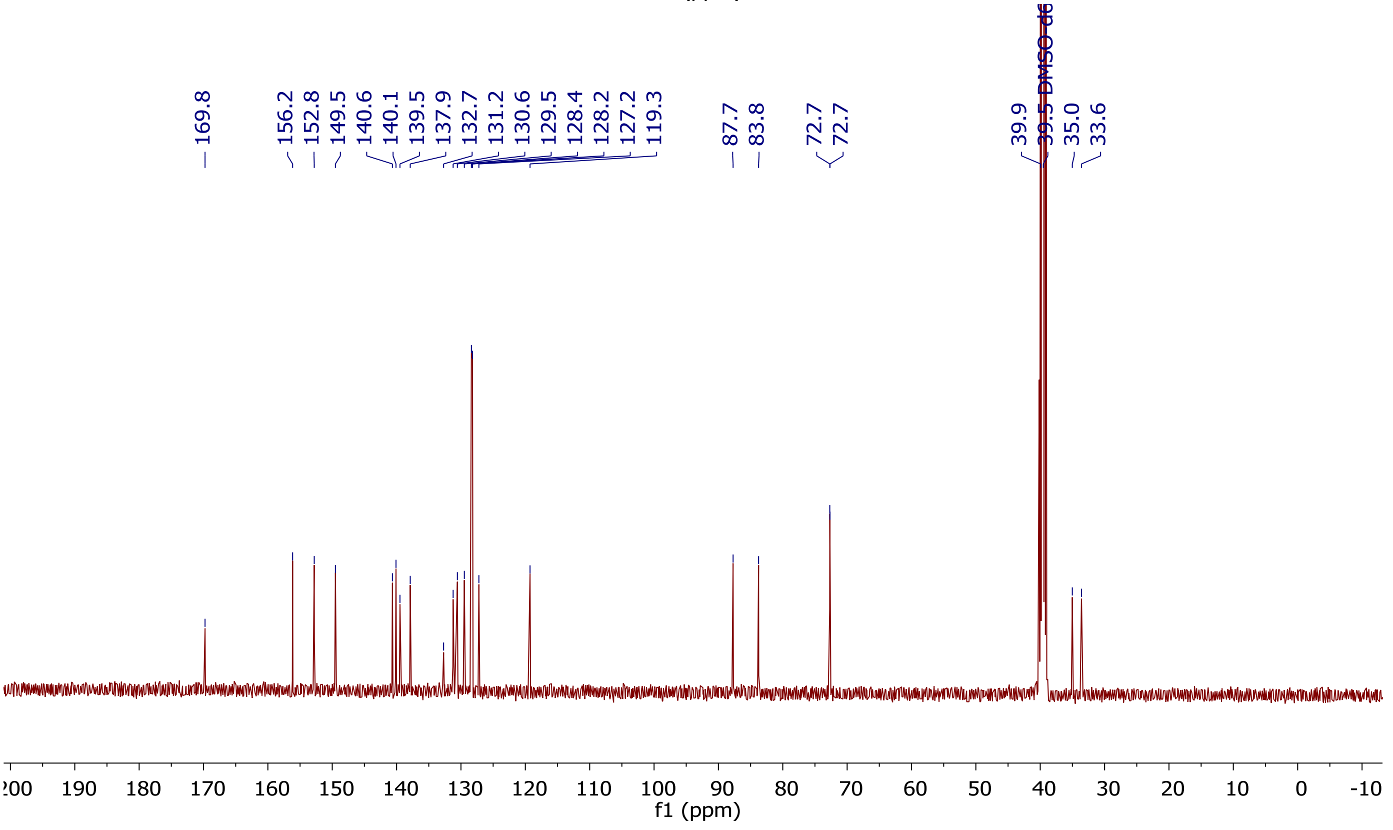
169.8
156.2
152.8
149.5
140.6
140.1
139.5
137.9
132.7
131.2
130.6
129.5
128.4
128.2
127.2
119.3
87.7
83.8
72.7
72.7
39.9
39.5 DMSO-d6
35.0
33.6
200 190 180 170 160 150 140 130 120 110 100 90 80 70 60 50 40 30 20 10 0 -10
f1 (ppm)

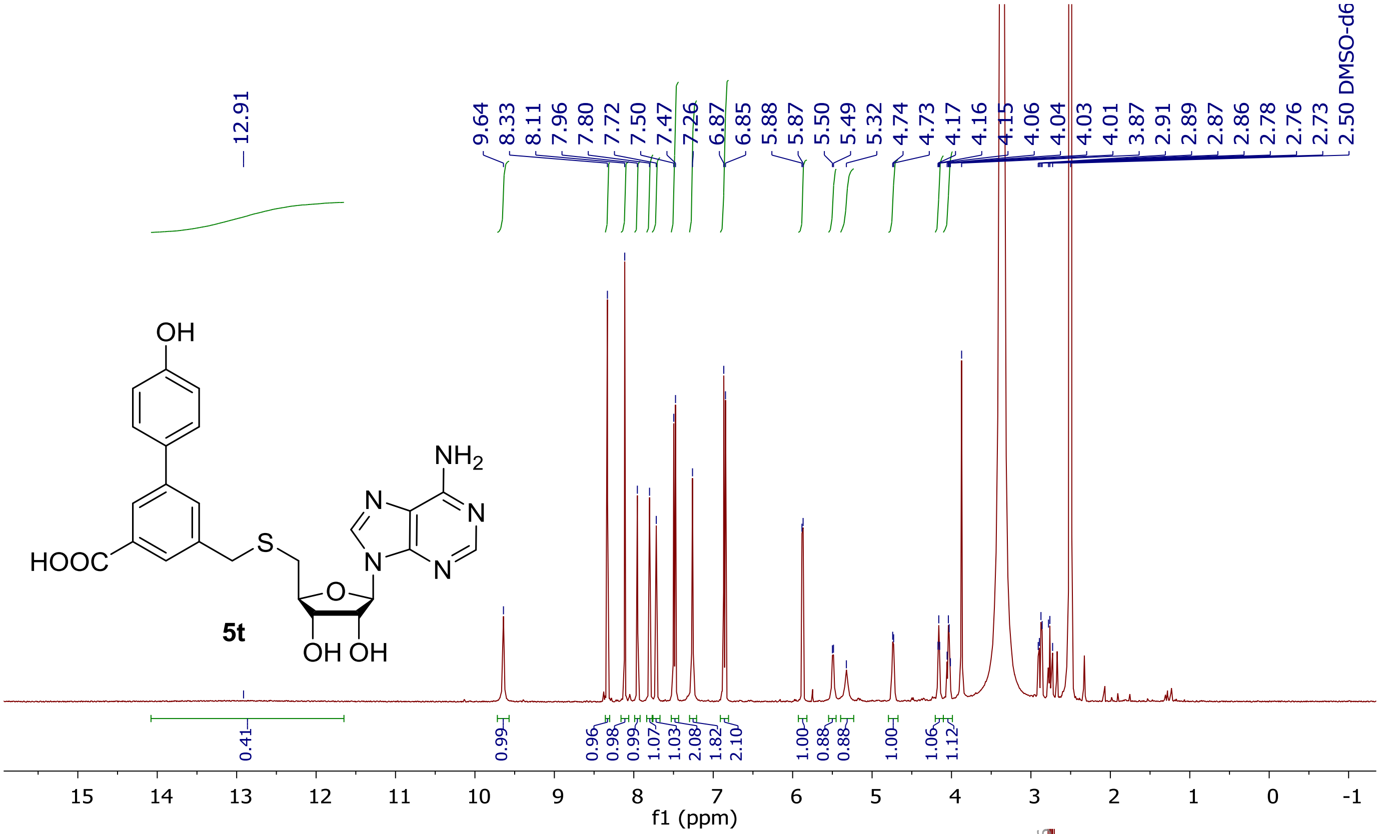
12.91
9.64 8.33 8.11 7.96 7.80 7.72 7.50 7.47 7.26 6.87 6.85 5.88 5.87 5.50 5.49 5.32 4.74 4.73 4.17 4.16 4.15 4.06 4.04 4.03 4.01 3.87 2.91 2.89 2.87 2.86 2.78 2.76 2.73 2.50 DMSO-d6
OH
NH2
N
HOOC
S
O
5t
OH OH
0.41 0.99 0.96 0.98 0.99 1.07 1.03 2.08 1.82 2.10 1.00 0.88 0.88 1.00 1.06 1.12
f1 (ppm)

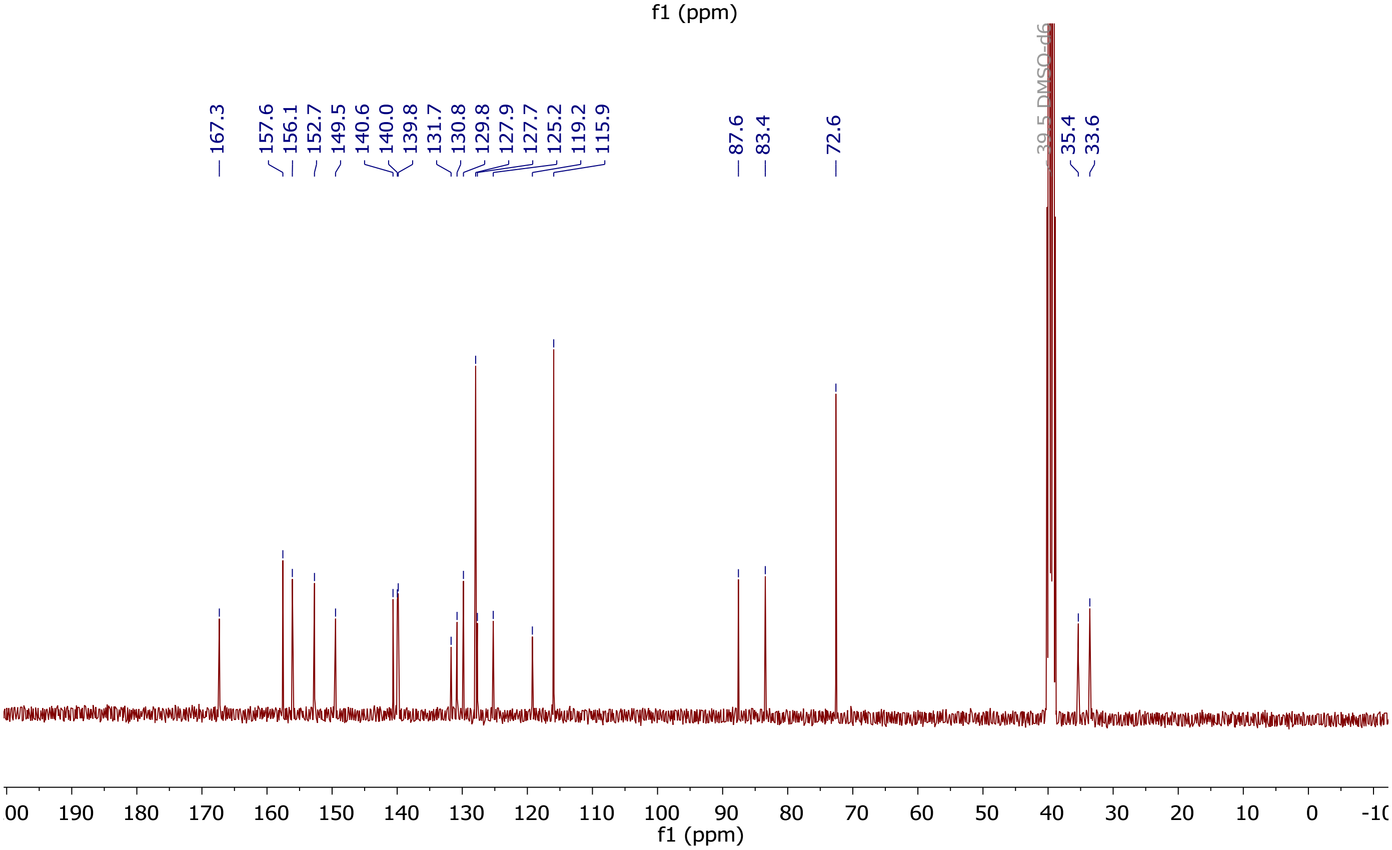
167.3 157.6 156.1 152.7 149.5 140.6 140.0 139.8 131.7 130.8 129.8 127.9 127.7 125.2 119.2 115.9 87.6 83.4 72.6 39.5 DMSO-d6 35.4 33.6
f1 (ppm)

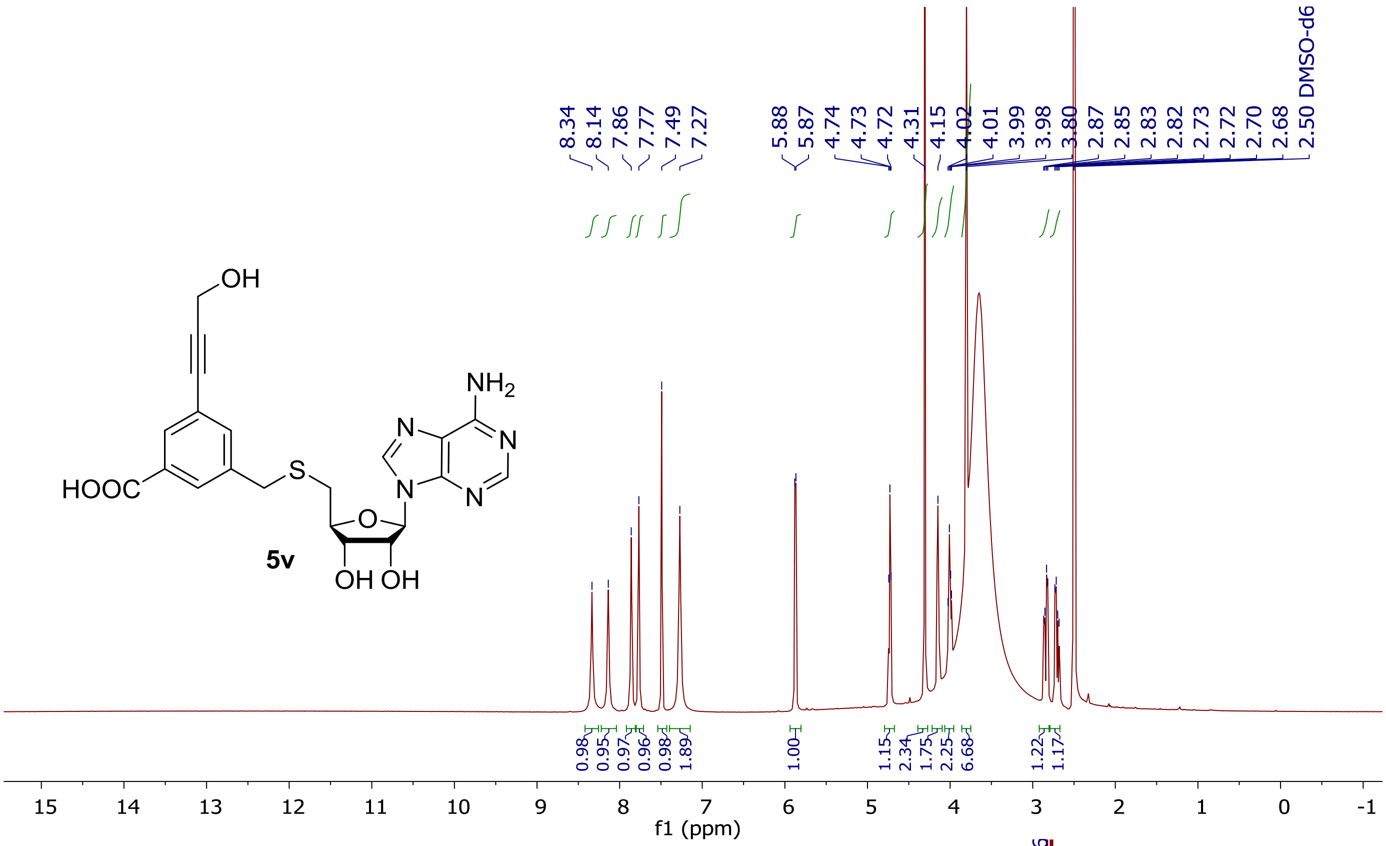
8.34
8.14
7.86
7.77
7.49
7.27
5.88
5.87
4.74
4.73
4.72
4.31
4.15
4.02
4.01
3.99
3.98
3.80
2.87
2.85
2.83
2.82
2.73
2.72
2.70
2.68
2.50 DMSO-d6
OH
NH2
N
HOOC
S
O
5v
OH OH
0.98
0.95
0.97
0.96
0.98
1.89
1.00
1.15
2.34
1.75
2.25
6.68
1.22
1.17
15 14 13 12 11 10 9 8 7 6 5 4 3 2 1 0 -1
f1 (ppm)

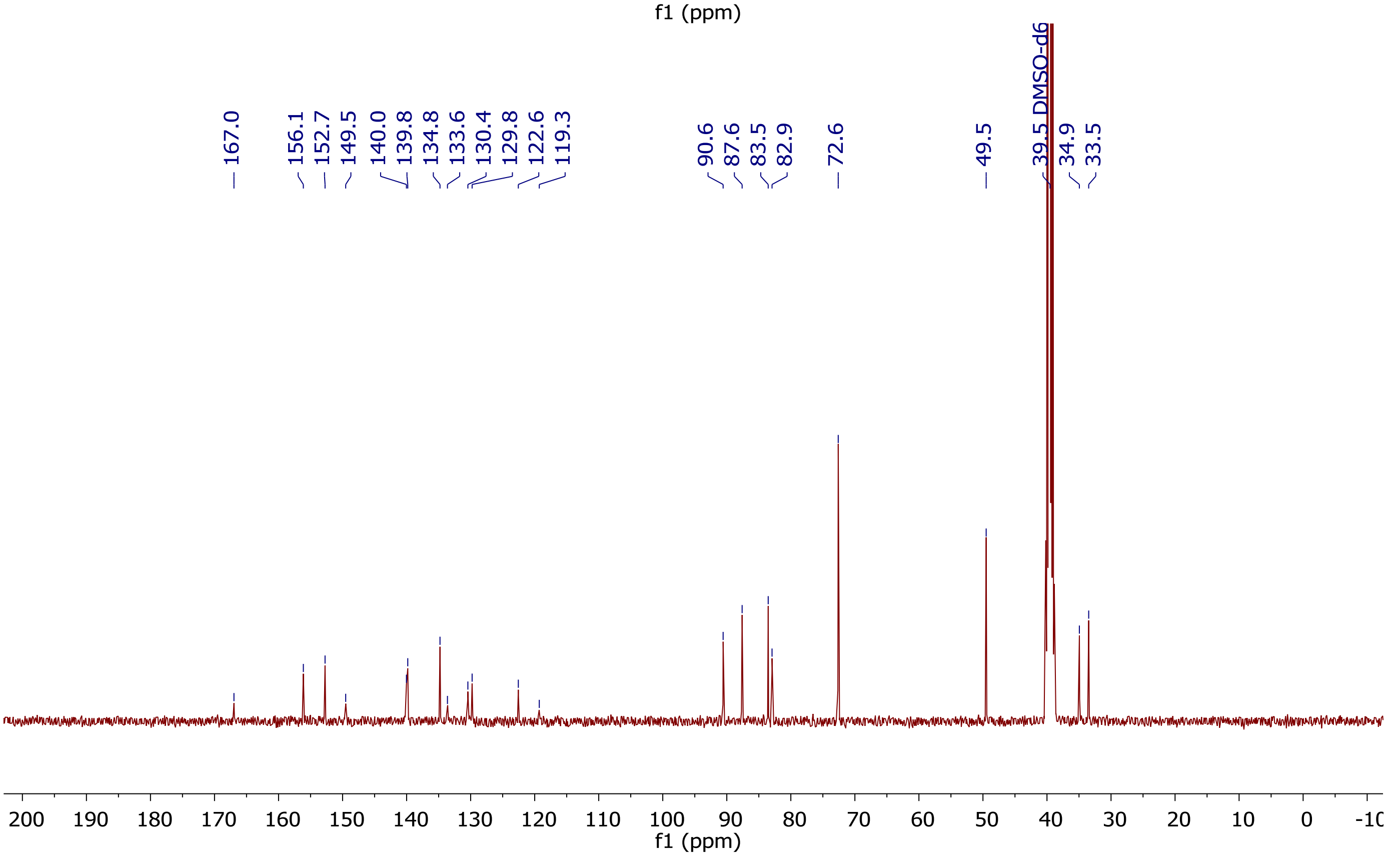
167.0
156.1
152.7
149.5
140.0
139.8
134.8
133.6
130.4
129.8
122.6
119.3
90.6
87.6
83.5
82.9
72.6
49.5
39.5 DMSO-d6
34.9
33.5
200 190 180 170 160 150 140 130 120 110 100 90 80 70 60 50 40 30 20 10 0 -10
f1 (ppm)

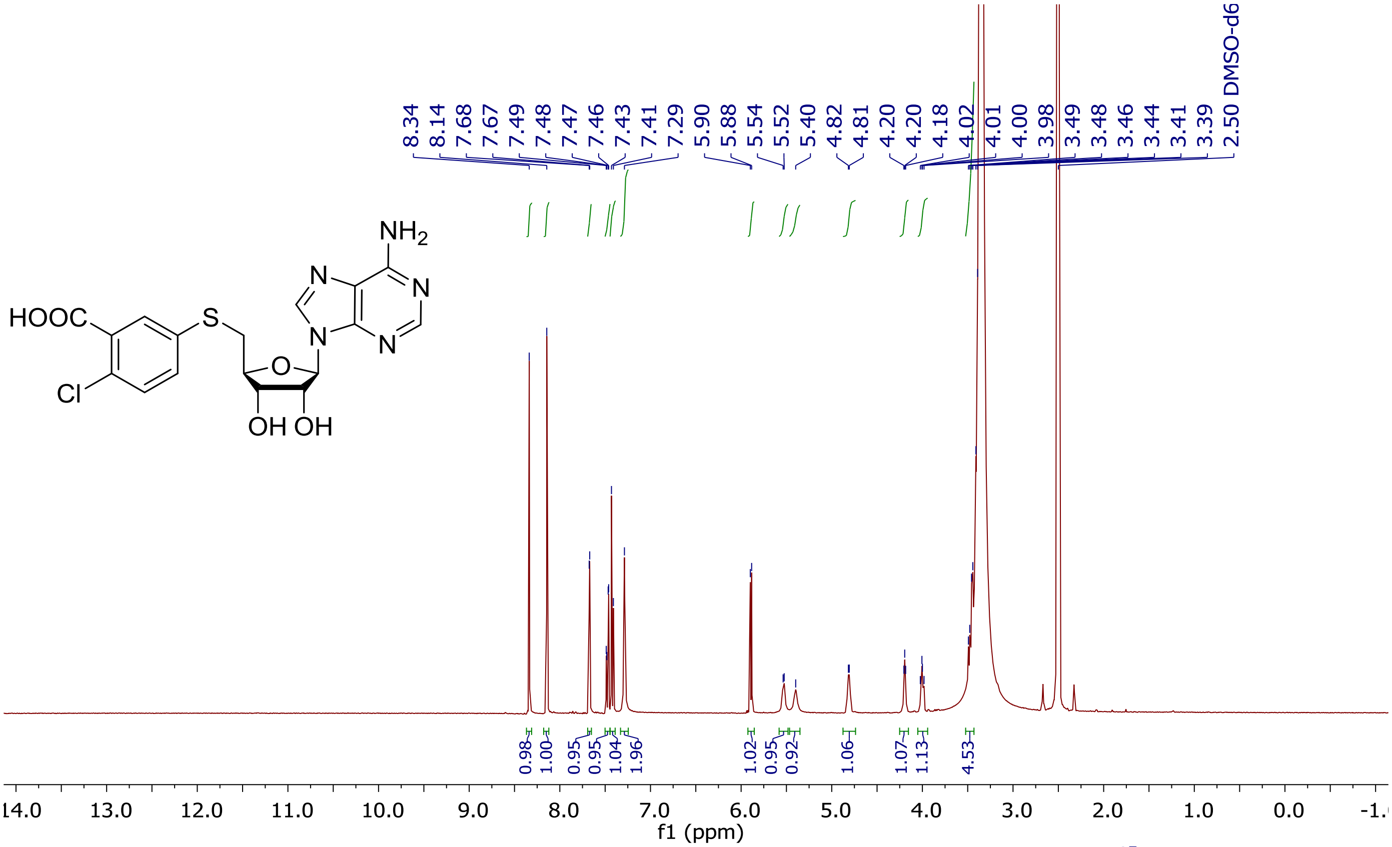
8.34
8.14
7.68
7.67
7.49
7.48
7.47
7.46
7.43
7.41
7.29
5.90
5.88
5.54
5.52
5.40
4.82
4.81
4.20
4.20
4.18
4.02
4.01
4.00
3.98
3.49
3.48
3.46
3.44
3.41
3.39
2.50 DMSO-d6
NH2
N
N
N
N
HOOC
S
O
Cl
OH OH
0.98
1.00
0.95
0.95
1.04
1.96
1.02
0.95
0.92
1.06
1.07
1.13
4.53
14.0 13.0 12.0 11.0 10.0 9.0 8.0 7.0 6.0 5.0 4.0 3.0 2.0 1.0 0.0
f1 (ppm)

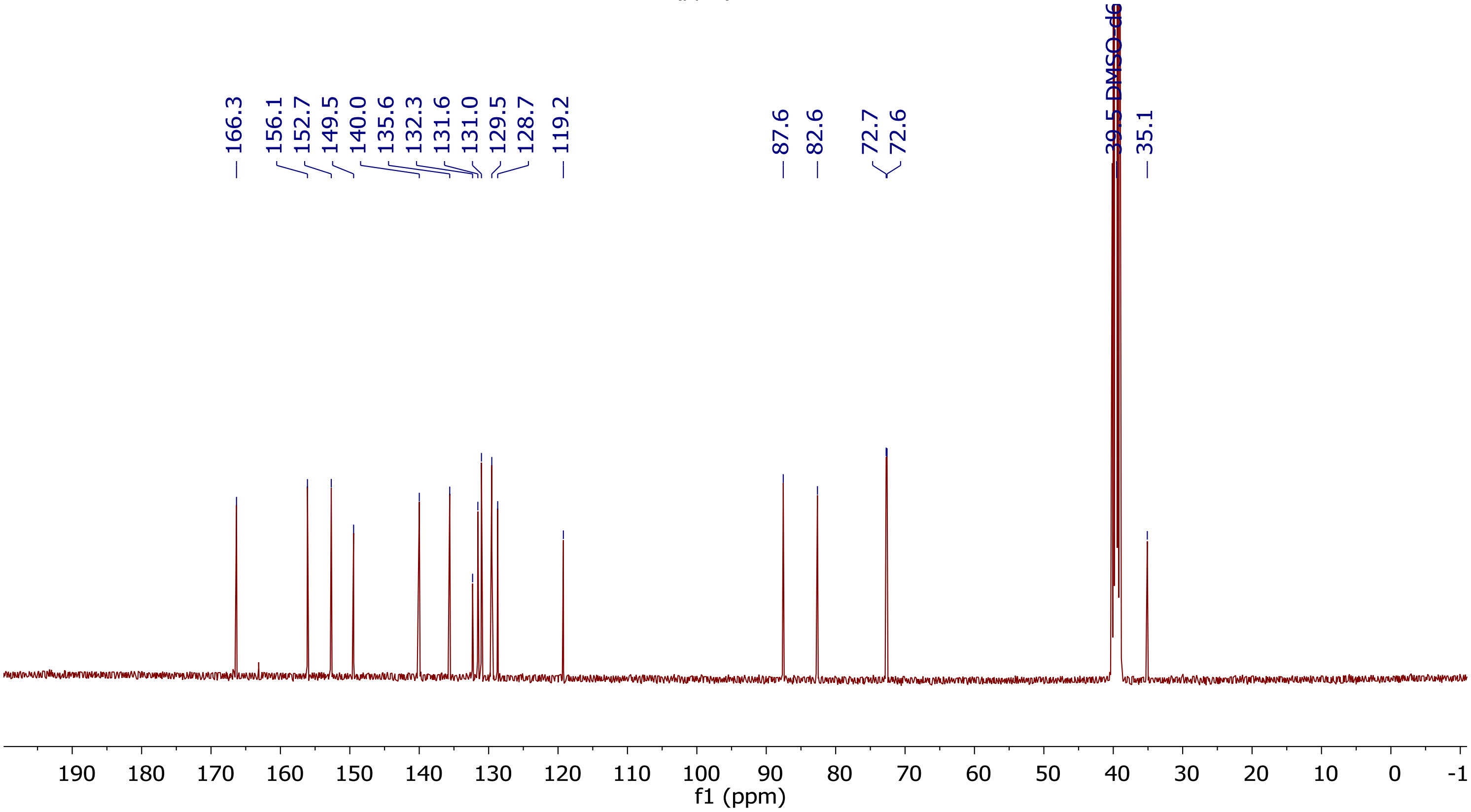
166.3
156.1
152.7
149.5
140.0
135.6
132.3
131.6
131.0
129.5
128.7
119.2
87.6
82.6
72.7
72.6
39.5 DMSO-d6
35.1
190 180 170 160 150 140 130 120 110 100 90 80 70 60 50 40 30 20 10 0
f1 (ppm)

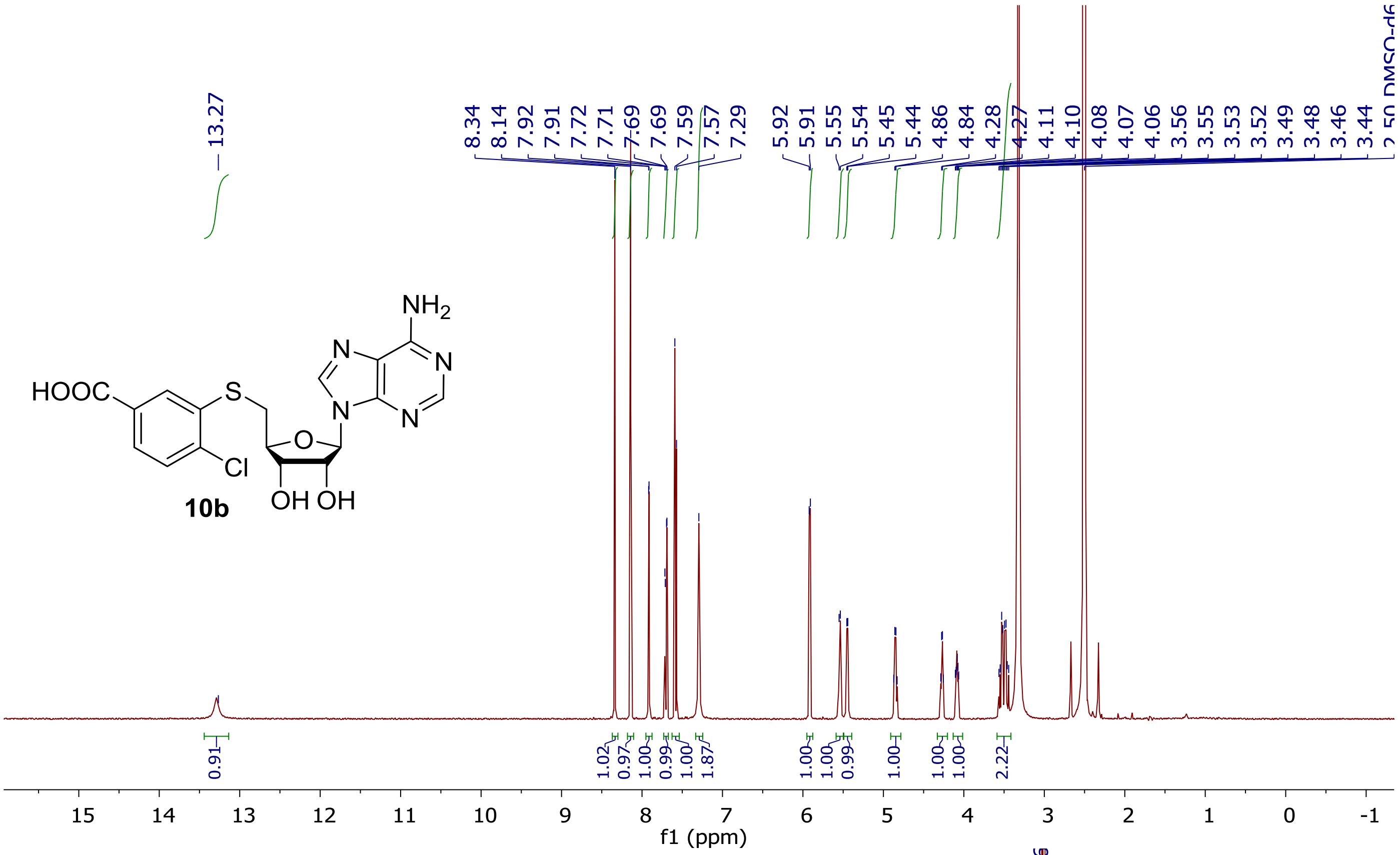

13.27
8.34 8.14 7.92 7.91 7.72 7.71 7.69 7.69 7.59 7.57 7.29
5.92 5.91 5.55 5.54 5.45 5.44 4.86 4.84 4.28 4.27 4.11 4.10 4.08 4.07 4.06 3.56 3.55 3.53 3.52 3.49 3.48 3.46 3.44
2.50 DMSO-d6
NH2
N
HOOC
S
O
Cl
OH OH
10b
0.91
1.02 0.97 1.00 0.99 1.00 1.87
1.00 1.00 0.99 1.00 1.00 1.00 2.22
15 14 13 12 11 10 9 8 7 6 5 4 3 2 1 0 -1
f1 (ppm)

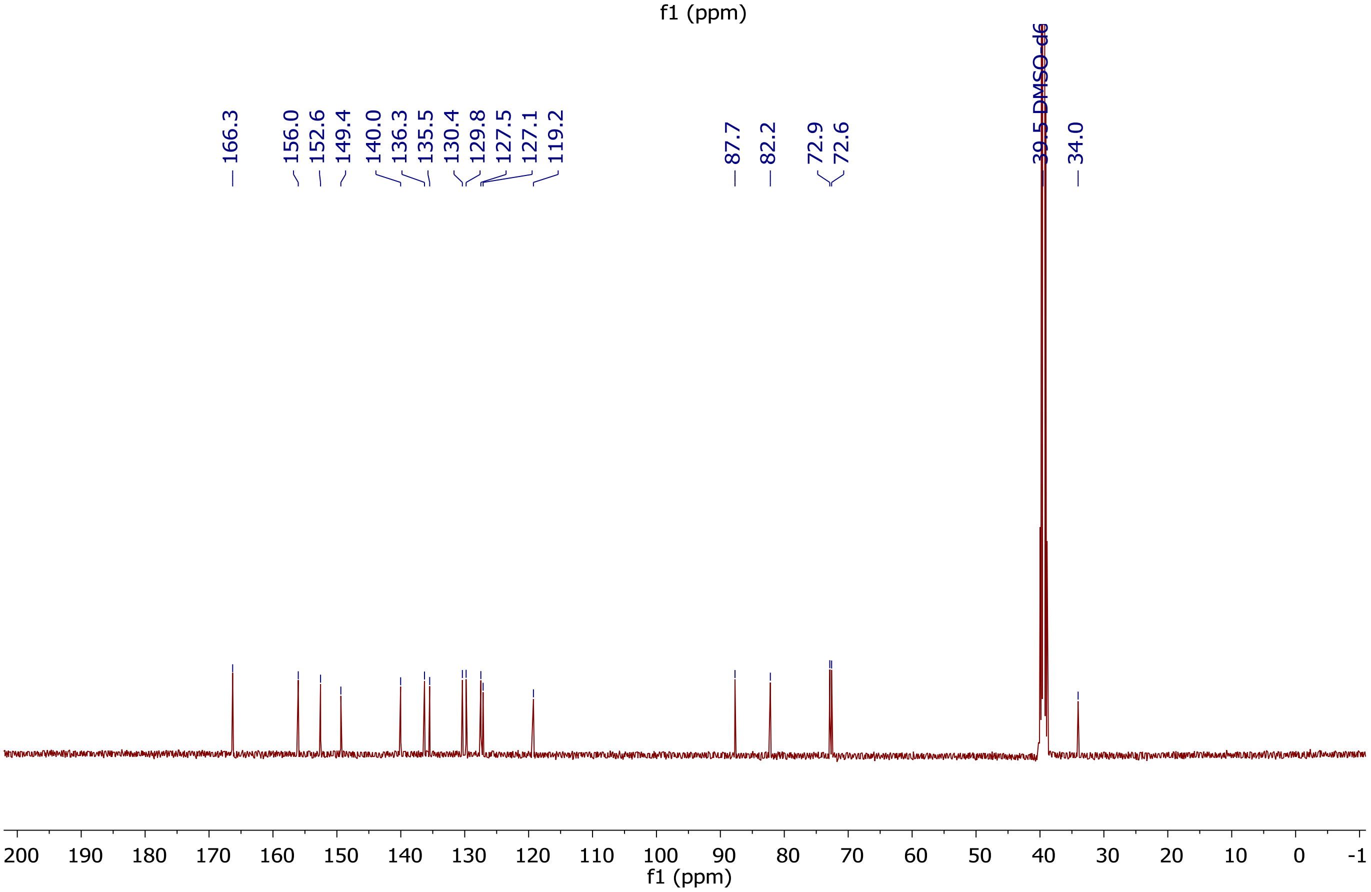

166.3
156.0 152.6 149.4 140.0 136.3 135.5 130.4 129.8 127.5 127.1 119.2
87.7
82.2
72.9 72.6
39.5 DMSO-d6
34.0
200 190 180 170 160 150 140 130 120 110 100 90 80 70 60 50 40 30 20 10 0 -1
f1 (ppm)

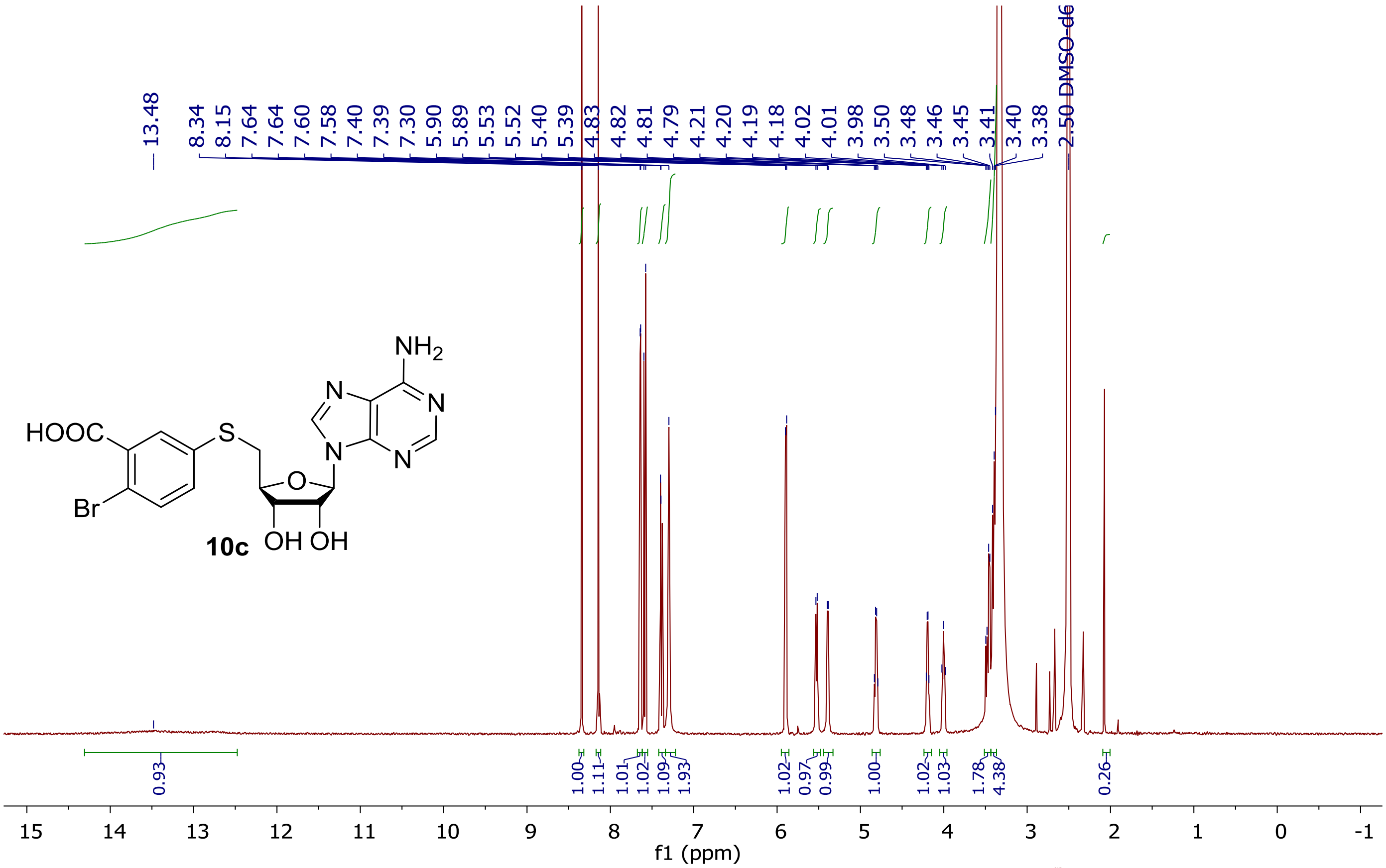

KFSL5-OBV-169.10.fid

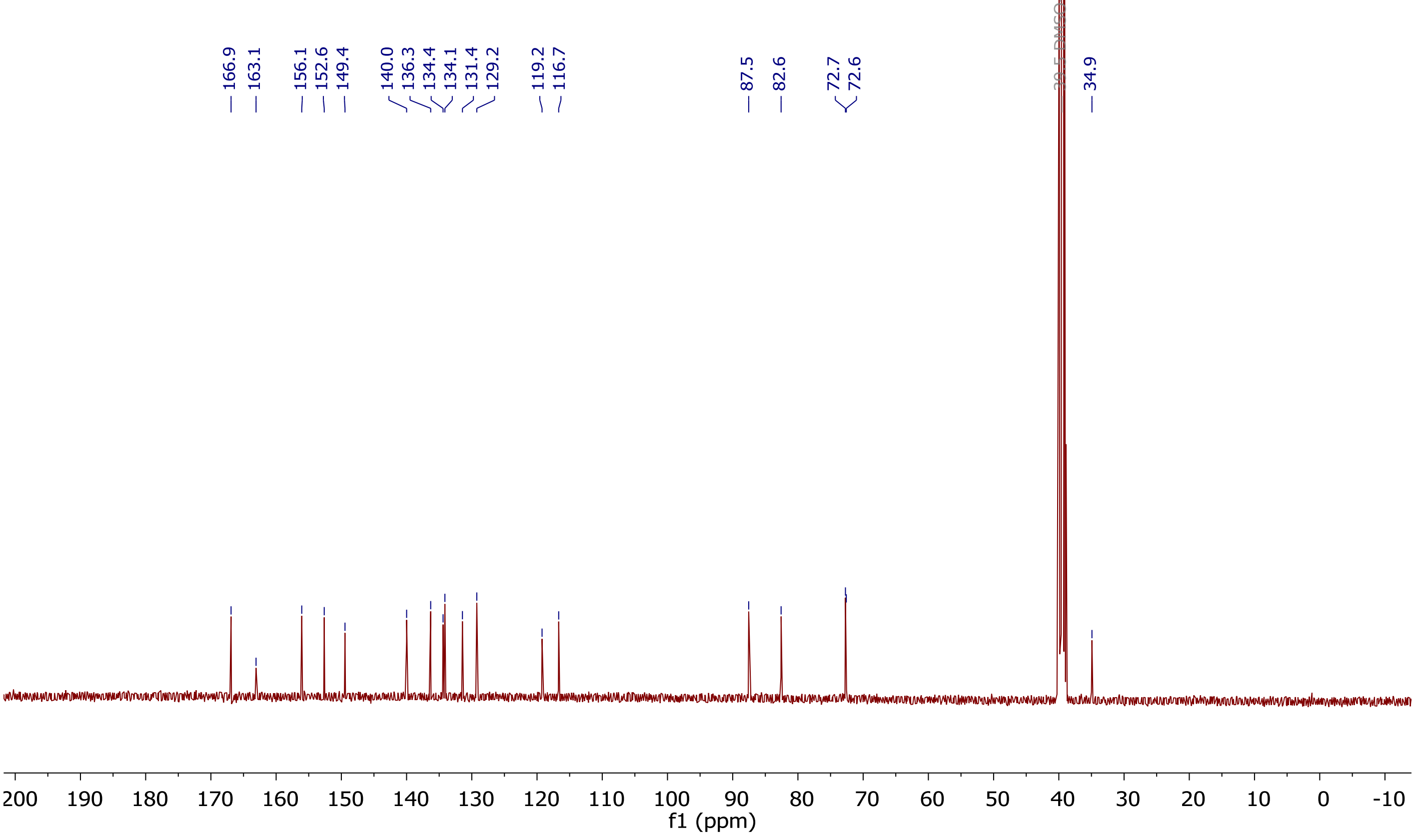

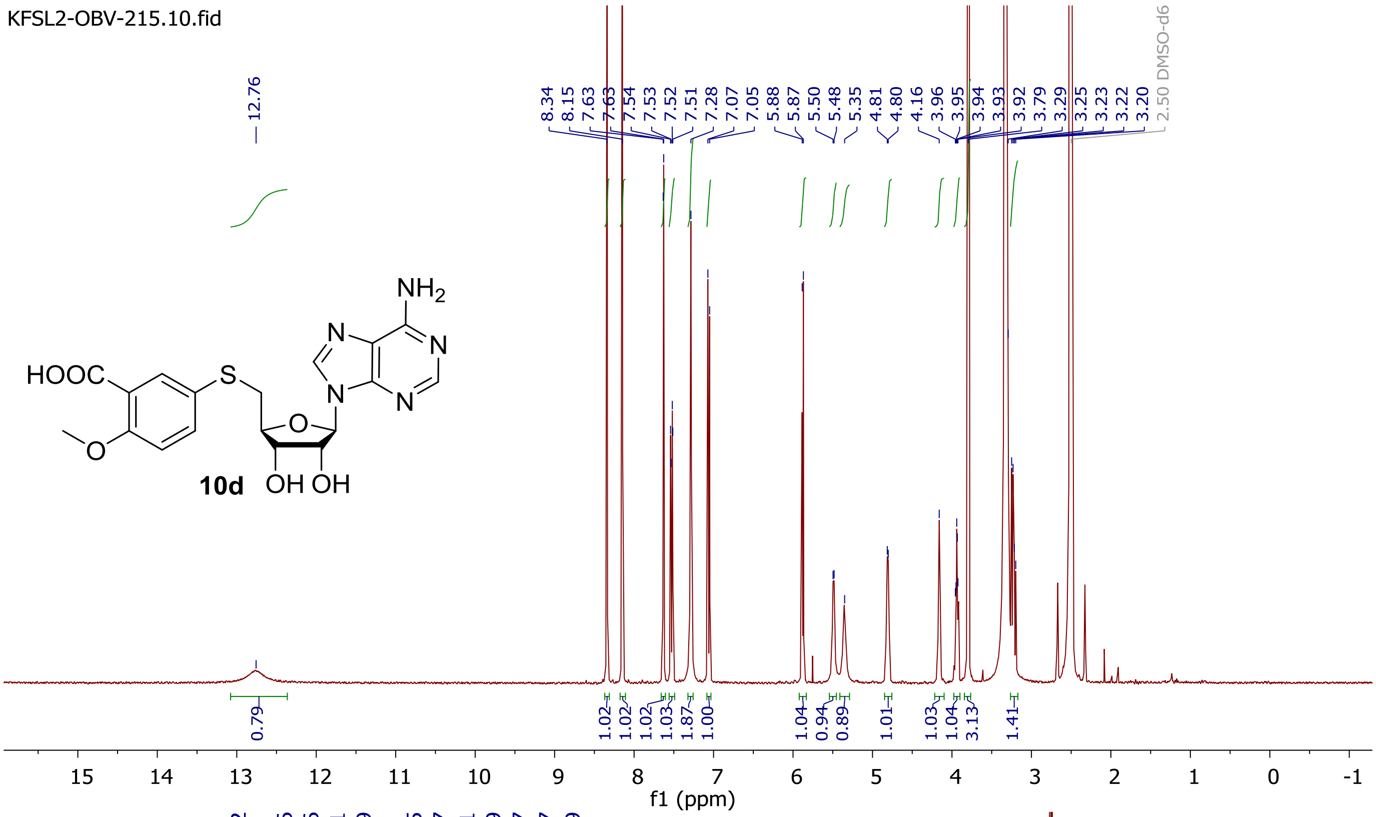
KFSL2-OBV-215.10.fid
12.76
8.34 8.15 7.63 7.63 7.54 7.53 7.52 7.51 7.28 7.07 7.05 5.88 5.87 5.50 5.48 5.35 4.81 4.80 4.16 3.96 3.95 3.94 3.93 3.92 3.79 3.29 3.25 3.23 3.22 3.20
2.50 DMSO-d6
NH2
N
N
N
N
HOOC
S
O
O
10d
OH OH
0.79
1.02 1.02 1.02 1.03 1.87 1.00
1.04 0.94 0.89
1.01
1.03 1.04 3.13
1.41
15 14 13 12 11 10 9 8 7 6 5 4 3 2 1 0 -1
f1 (ppm)

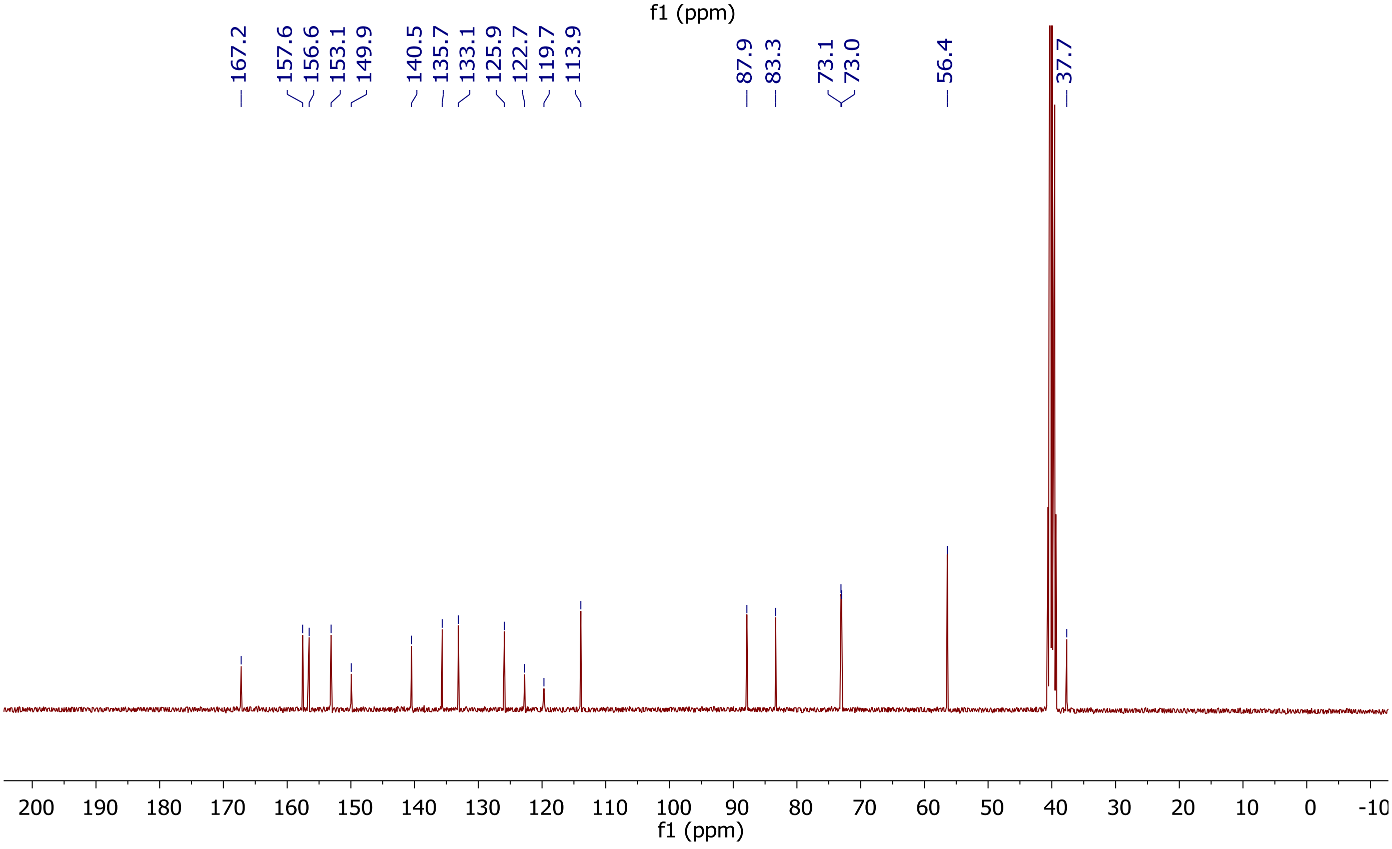
167.2
157.6 156.6 153.1 149.9
140.5 135.7 133.1 125.9 122.7 119.7 113.9
87.9 83.3
73.1 73.0
56.4
37.7
200 190 180 170 160 150 140 130 120 110 100 90 80 70 60 50 40 30 20 10 0 -10
f1 (ppm)

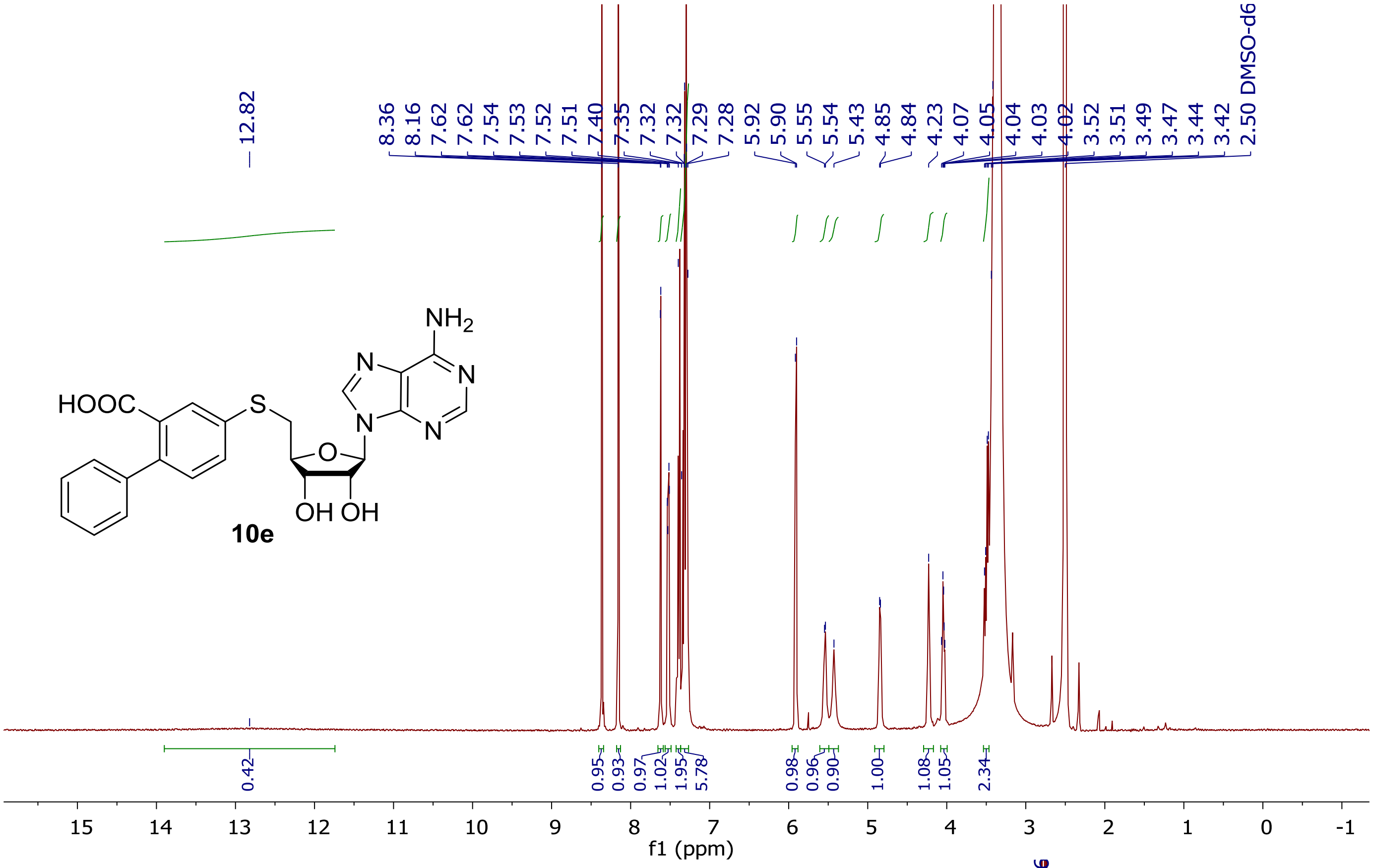
12.82
8.36 8.16 7.62 7.62 7.54 7.53 7.52 7.51 7.40 7.35 7.32 7.32 7.29 7.28 5.92 5.90 5.55 5.54 5.43 4.85 4.84 4.23 4.07 4.05 4.04 4.03 4.02 3.52 3.51 3.49 3.47 3.44 3.42 2.50 DMSO-d6
HOOC
S
O
N
NH2
OH OH
10e
0.42
0.95 0.93 0.97 1.02 1.95 5.78 0.98 0.96 0.90 1.00 1.08 1.05 2.34
15 14 13 12 11 10 9 8 7 6 5 4 3 2 1 0 -1
f1 (ppm)

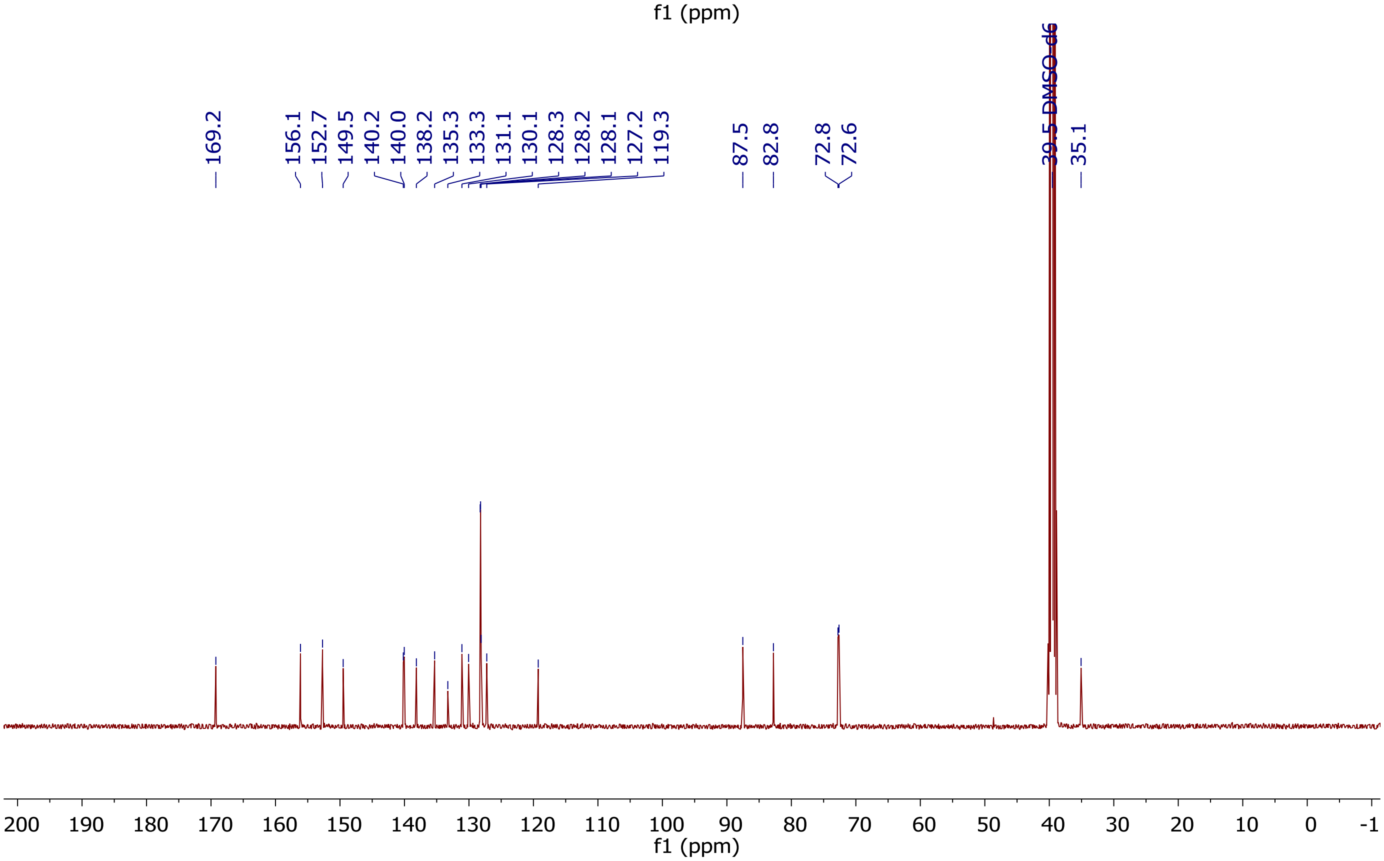
169.2
156.1 152.7 149.5 140.2 140.0 138.2 135.3 133.3 131.1 130.1 128.3 128.2 128.1 127.2 119.3
87.5 82.8 72.8 72.6
39.5 DMSO-d6
35.1
200 190 180 170 160 150 140 130 120 110 100 90 80 70 60 50 40 30 20 10 0 -1
f1 (ppm)

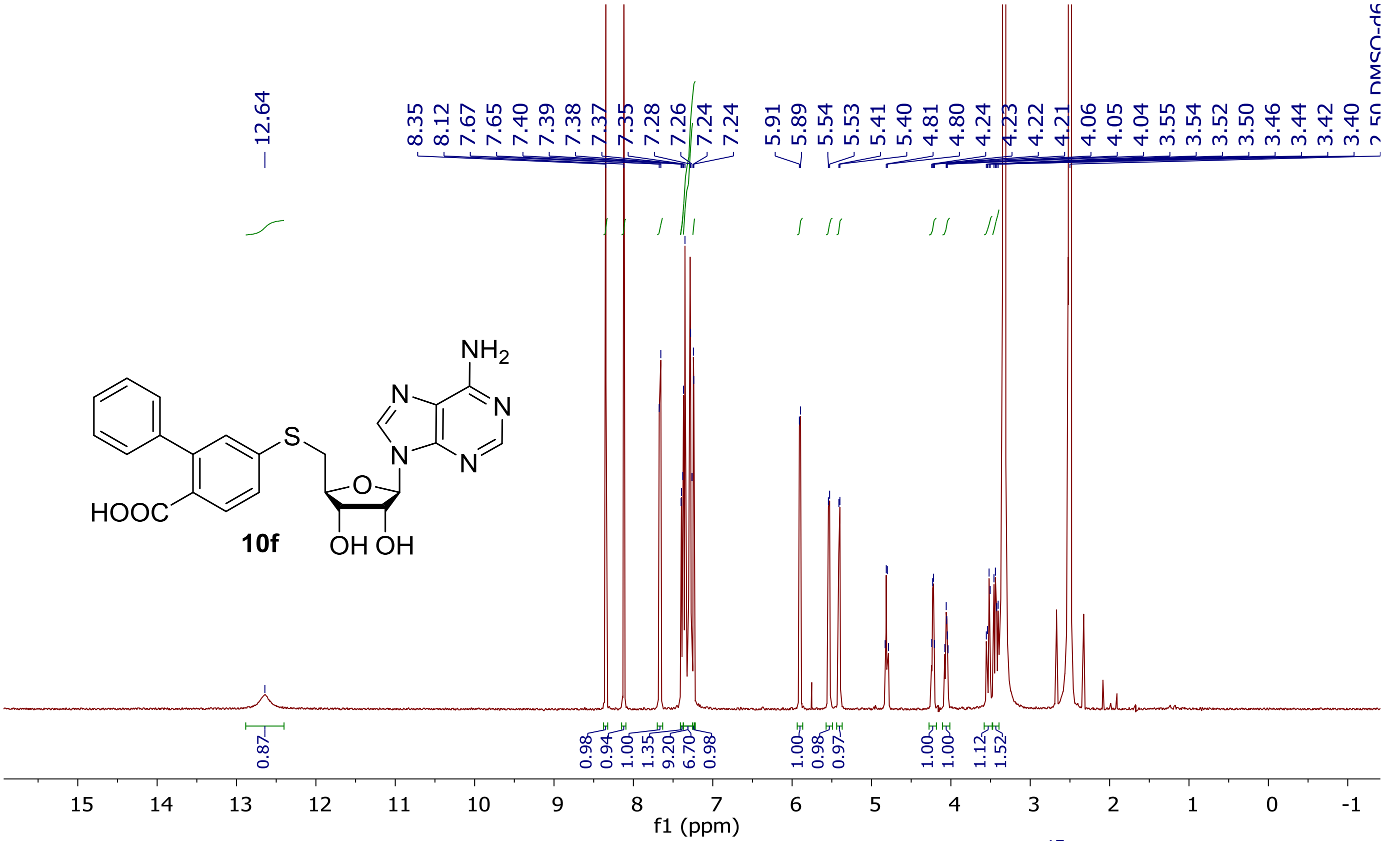
12.64
8.35 8.12 7.67 7.65 7.40 7.39 7.38 7.37 7.35 7.28 7.26 7.24 7.24
5.91 5.89 5.54 5.53 5.41 5.40 4.81 4.80 4.24 4.23 4.22 4.21 4.06 4.05 4.04 3.55 3.54 3.52 3.50 3.46 3.44 3.42 3.40 2.50 DMSO-d6
NH2
N
N
N
N
S
O
HOOC
OH OH
10f
0.87
0.98 0.94 1.00 1.35 9.20 6.70 0.98
1.00 0.98 0.97
1.00 1.00
1.12 1.52
15 14 13 12 11 10 9 8 7 6 5 4 3 2 1 0 -1
f1 (ppm)

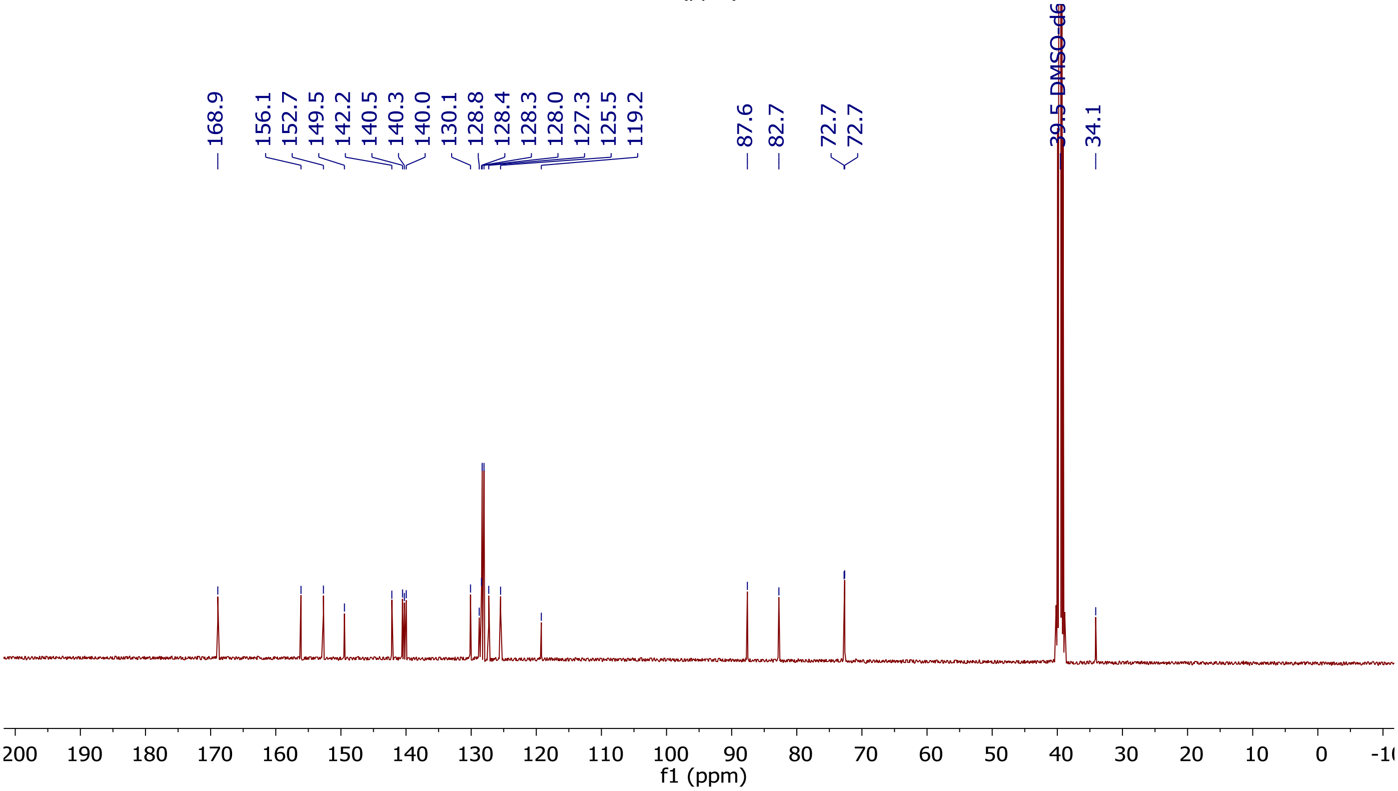
168.9
156.1 152.7 149.5 142.2 140.5 140.3 140.0 130.1 128.8 128.4 128.3 128.0 127.3 125.5 119.2
87.6
82.7
72.7 72.7
39.5 DMSO-d6
34.1
200 190 180 170 160 150 140 130 120 110 100 90 80 70 60 50 40 30 20 10 0
f1 (ppm)
